# HERMES: Holographic Equivariant neuRal network model for Mutational Effect and Stability prediction

**DOI:** 10.1101/2024.07.09.602403

**Published:** 2024-07-13

**Authors:** Gian Marco Visani, Michael N. Pun, William Galvin, Eric Daniel, Kevin Borisiak, Utheri Wagura, Armita Nourmohammad

**Affiliations:** 1 Department of Computer Science and Engineering, University of Washington, Seattle, USA; 2 Department of Physics, University of Washington, 3910 15th Avenue Northeast, Seattle, WA 98195, USA; 3 Department of Physics, Massachusetts Institute of Technology, 182 Memorial Dr, Cambridge, MA 02139; 4 Department of Applied Mathematics, University of Washington, Seattle, USA; 5 Fred Hutchinson cancer Research Center, 1100 Fairview ave N, Seattle, WA 98109, USA

## Abstract

Predicting the stability and fitness effects of amino acid mutations in proteins is a cornerstone of biological discovery and engineering. Various experimental techniques have been developed to measure mutational effects, providing us with extensive datasets across a diverse range of proteins. By training on these data, traditional computational modeling and more recent machine learning approaches have advanced significantly in predicting mutational effects. Here, we introduce HERMES, a 3D rotationally equivariant structure-based neural network model for mutational effect and stability prediction. Pre-trained to predict amino acid propensity from its surrounding 3D structure, HERMES can be fine-tuned for mutational effects using our open-source code. We present a suite of HERMES models, pre-trained with different strategies, and fine-tuned to predict the stability effect of mutations. Benchmarking against other models shows that HERMES often outperforms or matches their performance in predicting mutational effect on stability, binding, and fitness. HERMES offers versatile tools for evaluating mutational effects and can be fine-tuned for specific predictive objectives.

## I. INTRODUCTION

Understanding the effects of amino acid mutations on a protein’s function is a hallmark of biological discovery and engineering. Identifying disease-causing mutations [3, 4], enhancing enzymes’ catalytic activity [5, 6], forecasting viral escape [7–9], and engineering high-affinity antibodies [10], are just some of the areas of study that rely on accurate modeling of mutational effects.

Effects on protein stability are likely the most studied, as sufficient stability is usually a prerequisite of the protein’s successful carrying of its function [11]. Understanding the impact of mutations on the protein’s *binding affinity* to its partner is also crucial, as most functions are mediated by binding events. The effects on both stability and binding can be accurately measured experimentally, for example via thermal or chemical denaturation assays [12], by surface plasmon resonance [13]. However, these processes are laborious and time-intensive and do not scale up to high numbers of mutations. In recent years, Deep Mutational Scanning (DMS) emerged as a technique to simultaneously measure the effects of as many as 1 million mutations of a single protein, using various assays that usually directly depend on thermal stability and binding affinity, but often model more complex cellular activity [14–16].

Computational modeling of mutational effects remain an attractive alternative, due to its still much lower cost and required time. Methods based on molecular dynamics simulations are accurate but have limited benefits in terms of time and accessibility [17], whereas energy-function-based methods such as FoldX [18] and Rosetta [19] are well-established and still widely used.

Recently, machine learning models have challenged the dominance of energy-function-based models, most notably in protein folding [20], but also in stability and binding mutation effect prediction [4, 21]. In particular, machine learning models offer great improvements in speed. Several supervised models have been developed, though they often suffer from overfitting to the all-too-often limited and biased training data [22–25]. Self-supervised learning has emerged as a more robust alternative: instead of relying on experimental data, these models are usually trained to predict masked amino-acid labels conditioned on structural and/or sequence information, thus learning a probability distribution over amino-acids at a particular site conditioned on its context. The key insight has been that these learned distributions can be used to approximate mutational effects in zero-shot [1, 4, 21, 26–29]. However, zero-shot predictive power heavily depends upon the training data distribution and context used, and performance is often worse than that of energy-function-based models [21, 27].

Among the recently-developed self-supervised models is Holographic Convolutional Neural Network (HCNN) [1]. HCNN is a 3D rotation equivariant neural network [30] trained to predict amino-acids propensities at individual sites, using as input the atomic composition of the structural neighborhood within 10 Åof a focal residue. It was shown that HCNN appears to learn an effective physical potential of local atomic environments in proteins, and that it shows high-accuracy zero-shot predictions stability and binding effect of mutations in a few proteins.

Concurrent to HCNN was the development of RaSP [4], which proposed to construct representations of masked atomic environments from the output embeddings of a 3DCNN trained - like HCNN - to predict amino-acid propensities, and to use these representations as one of the inputs to a small model trained to predict stability effects (∆∆*G*) computed with Rosetta. RaSP was shown to be able to to predict stability effects at the same level of accuracy of Rosetta, but orders of magnitude faster, enabling a large-scale analysis of missense-variants.

Building upon HCNN and inspired by RaSP, we propose HERMES, a 3D rotation equivariant neural network with a more efficient architecture than HCNN, pre-trained on amino-acid propensity, and easy to fine-tune on custom experimental or computationally-derived mutational effects using our open-source code. We develop a suite of models pre-trained with different strategies, and fine-tune them for predicting the stability effects of mutations computed with Rosetta. We thoroughly benchmark HERMES models against other zero-shot and fine-tuned machine learning models in predicting mutational effects on stability, binding affinity, and various DMS assays. Our contributions are summarized as follows:

1. We updated the architecture of HCNN to be ~2.75x faster and with better amino acid classification performance.
2. We developed a fast and effective procedure to fine-tune pre-trained models on mutational effects. Our procedure leaves the interface of the pre-trained model unvaried, making it possible to seamlessly swap models in downstream pipelines.
3. We show a thorough benchmarking on stability, binding affinity, and DMS assays, and provide our evaluation data and code.
4. We provide easy-to-use code for extracting amino acid likelihoods and embeddings of site-centered atomic environments, which can be used from our github repository ^1^ or, more simply, from a google colab notebook ^2^.
5. We provide code and a procedure for fine-tuning our pre-trained models on arbitrary mutation effect data, available in our github repository.

## II. METHODS

### A. Development of HERMES

**FIG. 1.**
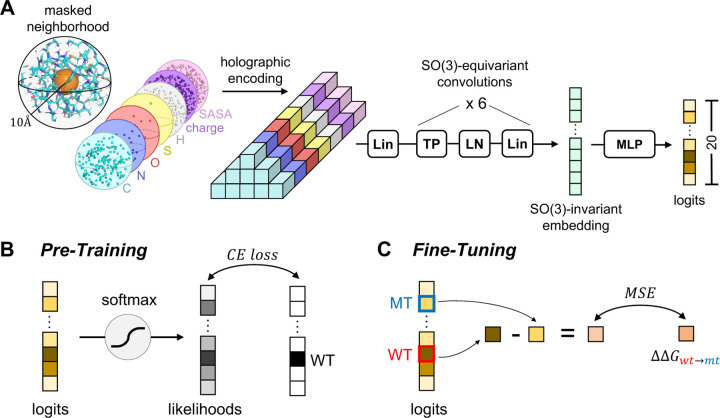
Schematic of HERMES’ architecture and training procedures. **(A)** Architecture of the updated Holographic Convolutional Neural Network (HCNN), which is the backbone of Hermes. The use of an efficient Tensor Product (TP) operation and foregoing of invariant skip connections makes this architecture 2.75x faster and with better performance on the pre-training task than the architecture in [1]; we refer the reader to [2] for details of the architecture. **(B)** Pre-training procedure. We train HCNN to predict the identity of the central neighborhood’s amino-acid, whose atoms have been masked. **(C)** Fine-tuning procedure for regressing over mutation effects. As shown in [1] and in this work, after pre-training the quantity log(*p*_*wt*_*/p*_*mt*_) correlates with experimental ∆∆*G*_*wt*→*mt*_ values of stability - and more broadly with mutation scores where a lower score indicates higher mutant fitness. Thus, we simply fine-tune HCNN to make the quantity log(*p*_*wt*_*/p*_*mt*_) regress over mutation scores. The result is a model whose implementation exactly matches the pre-trained model’s, making it trivial to substitute them for each other in downstream pipelines.

HERMES models are trained in two steps (Figure 1). First, following [1], we train an improved version of Holographic Convolutional Neural Network (HCNN) models to predict the amino acid type of a focal residue from its surrounding structural neighborhood. Specifically, we remove (mask) all atoms associated with the focal residue and predict the identity of its amino acid from the the other atoms within the structural neighborhood of radius 10 Å, centered at the focal residue’s C*α* (Figure 1B). Second, inspired by RaSP [4], we develop a procedure to fine-tune HERMES on mutation effects, specifically focusing on stability effects measured as ∆∆*G*.

#### HERMES architecture.

We build upon improvements to the HCNN architecture presented in [2], leading to a model that is ~2.75x faster, more accurate, with comparable number of parameters (~3.5M) as the architecture in [1] (Figure 1A). In short, atomic neighborhoods - i.e., featurized point clouds - are first projected onto the orthonormal Zernike Fourier Basis, centered at the (masked) central residue’s C*α*. We term the resulting Fourier encoding of the data an *holographic encoding*, as it presents a superposition of 3D spherical holograms [1]. Then, the resulting *holograms* are fed to a stack of SO(3)-Equivariant layers, which convert the holograms to an SO(3)-equivariant embedding - i.e. a representation that is invariant to 3D rotations about the center of the initial holographic projection. Finally, the embedding is fed to an MLP to generate the desired predictions. We refer the reader to [2] for details of the architecture, and for a mathematical introduction to building SO(3)-equivariant models in Fourier space. We implement HERMES using e3nn [31].

#### Pre-training data.

Following [1], we pre-train HERMES on neighborhoods from protein chains in ProteinNet’s CASP12 set with 30% similarity cutoff, and featurize atomic neighborhoods using atom type - including computationally-added hydrogens - partial charge, and Solvent Accessible Surface Area (SASA).

#### Preprocessing of protein structures.

Two classes of models using different types of pre-processing for the training data are presented in HERMES: pre-processing with (i) PyRosetta [1, 32], and (ii) BioPython [33] and other open source tools, adapting code from [4]. We refer to these models as HERMES PR and HERMES BP, respectively. To generate our open source pre-processed training data, we use the following procedure: we use OpenMM [34] to fix the PDB files, add missing residues and substitute non-canonical residues for their canonical counterparts; we use the reduce program [35] to add hydrogens; we take partial charges from the AMBER99sb force field [36]; we use BioPython to compute SASA [33]. Both preprocessings procedures keep atoms belonging to non-protein residues and ions, unlike RaSP [4]. Notably, our PyRosetta preprocessings does *not* replace non-canonical residues. HERMES BP facilitates non-academic applications, due to restricted licensing of PyRosetta.

#### Increasing robustness with ensembling and noise.

Each HCNN is an ensemble of 10 individually-trained model instances. Furthermore, we trained versions of HCNN after adding Gaussian noise to the 3D coordinates, with standard deviation 0.5 Å, and different random seeds for each of the 10 model instances.

#### Predicting mutation effects on protein fitness with HERMES.

HERMES, like HCNN, can be seen as a generative model of amino-acid labels for a residue, conditioned on the atomic environment surrounding the residue (what we call the residue’s *neighborhood* ). Prior work [1, 26, 27] has shown that conditional generative models of amino-acid labels can be successfully used to infer mutation effects on protein fitness in a zero-shot fashion. Specifically, the log-likelihood ratio between the probability of observing the mutant amino-acid at a give residue $i\;(aa_{i}=\text{mt})$ conditioned on its surrounding neighborhood $X_{i}$, which we denote by $p\left(aa_{i}=\text{mt}|X_{i}^{\left(\text{mt}\right)}\right)$, and that of the wildtype amino-acid $p\left(aa_{i}=\text{wt}|X_{i}^{\left(\text{mt}\right)}\right)$ can well approximate the fitness difference between the mutant and wildtype protein:

(1) $$F_{\text{mt}}-F_{\text{wt}}\propto \log\frac{p\left(aa_{i}=\text{mt}|X_{i}^{\left(\text{mt}\right)}\right)}{p\left(aa_{i}=\text{wt}|X_{i}^{\left(\text{wt}\right)}\right)}$$

where the superscript on $X_{i}$ indicates the structure from which the atomic neighborhood is extracted from, and the distinction made between the two neighborhoods in Eq. 1 emphasizes the possible reorganization and relaxation of the surrounding atoms following the substitution of the focal residue. When using HERMES to infer mutation effects in practice, the only structure available is usually that of the wildtype, providing an approximate estimate for fitness difference (denoted by $\hat{\cdot }$),

(2) $${\hat{F}}_{\text{mt}}-F_{\text{wt}}\propto \log\frac{p\left(aa_{i}=\text{mt}|X_{i}^{\left(\text{wt}\right)}\right)}{p\left(aa_{i}=\text{wt}|X_{i}^{\left(\text{wt}\right)}\right)}$$

We should emphasize that the presence of the mutant amino-acid and subsequent energy minimization can lead to a substantially distinct neighborhood compared to that of the wild type. However, access to experimentally-determined structures for a large number of mutants is usually limited. Computational programs like Rosetta [32] can be used to “relax” the wildtype protein after applying the mutation *in-silico*, but the procedure can be inaccurate and is impractical at large scales. We test the use of mutant structures - both experimentally and *in-silico* determined- on a few datasets.

While Eq. 1 is specific to a single residue i, all methods presented and benchmarked in this work handle predictions of multiple mutations by simply adding the individual mutations’ log-likelihood ratios, thereby assuming independence between the mutations. Although this model can capture some correlations between the nearby (in structure) residues due to their shared neighborhoods, it does not fully account for all the epistatic interactions throughout a protein, and further developments are needed to accurately model epistasis.

Using the appropriate conditioning ($X_{i}$ in Eq. 1) is likely the most important factor in modeling a given type of fitness. For example, if attempting to predict binding affinity between a protein and its binding partner, it is logical that including the atoms of the binding partner in the neighborhood of the focal residue would be a prerequisite for successful modeling.

#### Fine-tuning on stability effect of mutations.

Inspired by RaSP [4], we develop a procedure to fine-tune HERMES on mutation effects. RaSP was developed by training an additional regression model for the stability effect of mutations, taking as inputs embeddings from a pre-trained 3DCNN and the identities of wildtype and mutant amino-acids. Unlike RaSP, we directly fine-tune the model itself to make the the predicted log-likelihood ratio in Equation 2 regress over mutation effects on stability. Importantly, our fine-tuning procedure preserves the interface to the pre-trained model, thus enabling the two models to be seamlessly swapped in downstream pipelines.

To greatly speed-up convergence, as a first step of fine-tuning we rescale the weight matrix and bias vector of the network’s output layer so the mean and variance of the output logits become the same as that of the training scores. This step requires one initial pass through the training data to get the mean and variance, but it makes the model outputs immediately be in the same distribution as the scores, thus avoiding epochs of fine-tuning just devoted to rescaling the model outputs. In this work, we fine-tune the models on the same stability ∆∆*G* values used by RaSP, which are computed with Rosetta for 35 training proteins (also we follow the same Fermi function (sigmoid) transform, as detailed in [4]). Using the re-scaling initialization outlined above, a batch size of 128 and learning rate of 5*e*^−4^, we find that the fine-tuning converges on this data within 1–2 epochs only. We provide easy-to-use code to fine-tune our pre-trained models on arbitrary mutation effect data. Importantly, as ∆∆*G* is an energy, with lower values indicating more stable structures, *we implement the fine-tuning with the physics convention that lower target values indicate higher stability and fitness, i.e., the negative of Eq. 2 is fit to the data.* In practice, to use the fine-tuning code, just make sure that lower means higher fitness, which can be done by simply flipping the sign of all the target values.

We pre-train 4 baseline HERMES models, with combinations of different protein pre-processings - PyRosetta (HERMES PR) vs. BioPython (HERMES BP) - and varying levels of Gaussian noise injected to the training data - 0.00 vs. 0.50 Å. We then fine-tune each model on Rosetta computed ∆∆*G* values, resulting in eight total HERMES models (baseline and fine-tuned).

### B. Alternative methods

We compare both the baseline and the fine-tuned HERMES models with the following models that are commonly used to predict the effect of mutations.

#### RaSP [4].

This is the method most similar to ours, developed concurrently to the first version of HCNN [1]. Similar to HERMES, RaSP is trained in two steps: first, a neural network - specifically a 3DCNN - is pre-trained to predict masked amino-acids from their local atomic environment (i.e. ”neighborhood”). Then, a small fully-connected neural network with a single output is trained to regress over mutation effects, using as input neighborhoods’ embeddings from the 3DCNN, the one-hot encodings of wildtype and mutant amino-acids, and the wildtype and mutant amino-acids’ frequencies in the pre-training data. RaSP is fine-tuned on the stability effect of mutations ∆∆*G*, computationally determined with Rosetta [32], which we also use to fine-tune HERMES. We do not reproduce results of RaSP in this work, and instead show the values reported in the paper.

#### ProteinMPNN [28].

ProteinMPNN is a tool for protein inverse-folding. The tool is most commonly used to sample amino-acid sequences conditioned on a protein’s backbone structure, and optionally a partial sequence. As ProteinMPNN also outputs probability distributions of amino-acids for the sites that are to be designed, it can also be used to infer mutation effects by computing the log-likelihood ratio presented in Eq. 1. Like for HERMES, we consider ProteinMPNN models trained with two noise levels: 0.02 Å(virtually no noise) and 0.20 Å. We provide scripts to infer mutation effects built upon a public fork of the ProteinMPNN repository.

#### ESM-1v [27].

This is the Protein Language Model (PLM) of the ESM family trained specifically for improved zero-shot predictions of mutation effects. As the training objective is predicting amino-acids that have been masked from the sequence, mutation effects are also predicted using the log-likelihood ratio (Eq. 1). To our knowledge, this is the strongest representative of PLMs for inferring mutation effects. We show a mix of previously-reported scores, and scores computed using their codebase. For our in-house ESM-1V predictions, wildtype sequences were obtained from the corresponding PDB file and verified against the European Bioinformatics Institute’s PDBe database via their REST API [37]. Mutation effect predictions were computed with ESM’s built-in *wildtype marginal* method; we attempted using the *masked marginal* method but ran into several errors, so we stuck to *wildtype marginal* as it was more reliable, and also had very similar performances in the few instances in which both methods worked.

#### DeepSequence [26].

This is a state-of-the-art model for inferring mutation effects from sequence alone. It uses a variational auto-encoder of full protein sequences to and infers mutation effects via Eq. 1. We only show previously-reported scores.

## III. RESULTS

### A. Model Validation

#### Validation on pre-training task.

We compute Cross-Entropy (CE) loss and classification accuracy on the pre-training task of predicting the (wildtype) amino-acid from its masked neighborhood, on 40 protein crystal structures from the CASP12 ProteinNet Test Set (Table I). Accuracy is computed using the highest-probability amino-acid as prediction, and discarding all other information. As expected, models trained with noisy structures exhibit slightly worse CE and accuracy. Interestingly, fine-tuned models exhibit much worse CE loss, but accompanied by a only slight drop in accuracy. This indicates that fine-tuned HERMES models output probability distributions with much higher entropy, while largerly preserving the original relative ranking of amino-acid preferences. This could, however, just be an artifact of the scale of predictions for fine-tuning, which, for the fine-tuning data we use, is in the [0, 1] range. Indeed, scaling down loagit values naturally increases the entropy of the resulting probability distribution after applying the softmax function.

#### Validation on fine-tuning task.

**FIG. 2.**
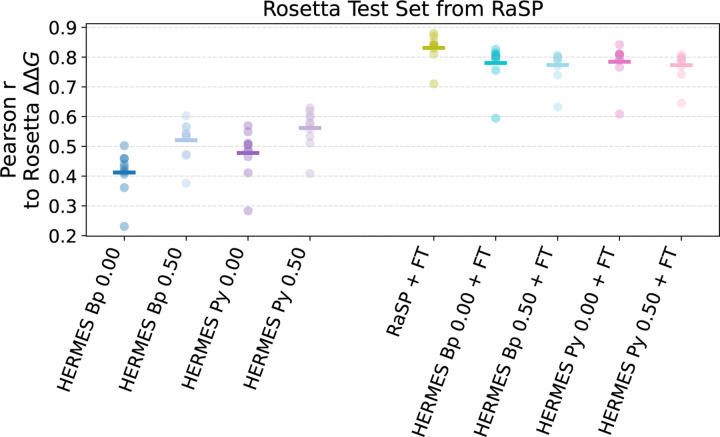
Prediction performance of RaSP and HERMES models on 10 Rosetta ∆∆*G* test proteins from the RaSP codebase [4]. Zero-shot models on the left, models fine-tuned on Rosetta ∆∆*G* on the right. This set serves as the de-facto test set of models fine-tuned with Rosetta ∆∆*G*. Pearson correlation is computed between model predictions and ∆∆*G* computed with Rosetta. Notably, zero-shot HCNN models benefit from both noise and from the use of PyRosetta for preprocessing. Instead, fine-tuned models perform similarly to each other.

Like RaSP, we test the performance of HERMES models on inferring the relative Rosetta-computed ∆∆*G* values on 10 test proteins (Figure 2). HERMES models that have *not* been fine-tuned exhibit a zero-shot performance around 0.4/0.5 Pearson r. Models trained with noise are significantly better and models that use PyRosetta pre-processing are better than those using BioPython. Fine-tuned HERMES models perform around 0.8 Pearson r, close but slightly below the level of RaSP, and with no significant difference across noise levels and preprocessing procedure.

### B. Prediction of stability effects ∆∆*G*

#### Analysis of single amino acid substitutions.

**FIG. 3.**
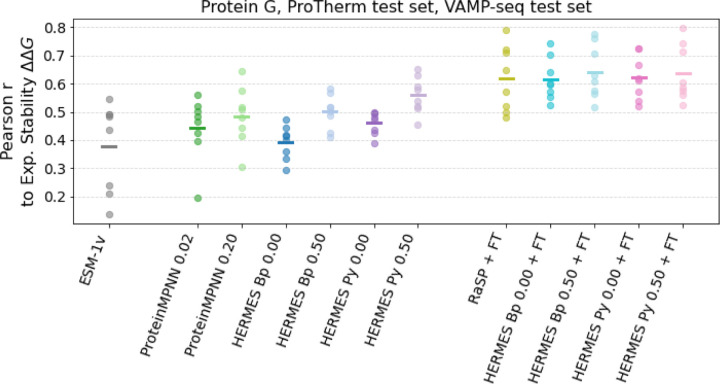
Prediction performance on experimental ∆∆*G* for eight test proteins. Zero-shot models on the left and center, models fine-tuned on Rosetta ∆∆*G* on the right. Each dot represents the value for a protein, and the horizontal bar is the mean. ESM-1v predictions were made using the wildtype marginals method. Similarly to Figure 2, zero-shot models - both HERMES and ProteinMPNN - benefit from training on noisy structures, whereas fine-tuned models all perform comparably. See Table S1 for detailed results.

We evaluate the ability of HERMES to infer experimental ∆∆*G* values for stability effect of mutations, first by considering values for eight proteins - curated by [4] - that present many single amino-acid substitutions. Specifically, we consider four proteins from ProTherm [38], the B1 domain of Protein G [39], and three proteins for which VAMP-seq values are available [40]. Variant Abundance by Massively Parallel sequencing (VAMP-seq) is a type of deep mutational scanning that probes mutation effects on cellular protein abundance, and was found to correlate with *in-vitro* measurements of stability of mutations ∆∆*G*, using different experimental methods [40]. Figure 3 shows the Pearson correlations between the stability effect of mutations ∆∆*G* and predictions using ESM-1v, ProteinMPNN, RaSP, and HERMES models. Zero-shot models perform significantly better when trained with noise, and HERMES models using PyRosetta preprocessing perform better than BioPython. ESM-1v, the only sequence-based model we consider here, performs the worst on average, but it is the best one for one protein from the VAMP-seq set (Table S1). The best zero-shot model is HERMES with 0.50 Å noise and PyRosetta preprocessing, with average Pearson r of 0.56. Models fine-tuned on Rosetta ∆∆*G* values, instead, all perform with a Pearson r larger than 0.60. RaSP and both of the HERMES models *without* noise have an average Pearson correlation of 0.62, whereas both HERMES models with 0.50 Å noise have a slightly better correlation of 0.64; see Table S1 for detailed results.

#### Using experimentally resolved mutant structures for predictions.

As noted in Eq. 1, our predictions should ideally account for the change in the surrounding protein structure neighborhoods due to substitutions in the focal residue. Here, we test the hypothesis as whether access to he true mutant structure could enhance the accuracy of our predictions relative to the approximate approach of using only the wildtype structure (Eq. 2). To do so we will focus on the well-studies protein T4-Lysozyme, for which there exists many measurements for stability effect of mutations ΔΔG together with their corresponding protein structures [41–58]. Table II shows prediction based on ProteinMPNN and HERMES models for these T4-Lysozyme mutants.Interestingly, HERMES zeros-hot models *do not* benefit from the use of the mutant structures, while ProteinMPNN zeros-hot and HERMES fine-tuned models do.

#### Prediction on additional datasets, and anti-symmetry wrt. the direction of mutations.

We further consider two additional datasets collected by [4]. The S669 experimental dataset, which includes 669 mutations across 94 protein structures, and the Ssym+ dataset, which includes 352 mutants across 19 protein structures [59]. In addition, Ssym includes experimentally-determined structures of all of the 352 mutants. These structures can be used to test whether our model correctly predicts the anti-symmetry condition, whereby the ∆∆*G* of a given mutation must be equal to that of its *reverse* mutation with opposite sign. If for example the *direct* mutation is G→A, then the reverse is A→G.


(3) $$\Delta \Delta G_{wt\to mt}=-\Delta \Delta G_{mt\to wt}$$

For S669 and Ssym-direct, we report two orthogonal computations of Pearson correlation: “Per Structure” correlations are computed by averaging correlations across proteins with at least 10 mutations; “Overall” correlations are instead computed across all mutations among all proteins. We consider “Per Structure” correlation as it is more closely related to practical use when designing highly-stable proteins by mutating from a starting protein. HERMES PRwith 0.50 Å noise and fine-tuning is consistently the best-performing model (Table S2). Interestingly, on S669 the Per Structure correlations are consistently higher than Overall correlations, and the opposite is true for Ssym-direct.

**FIG. 4.**
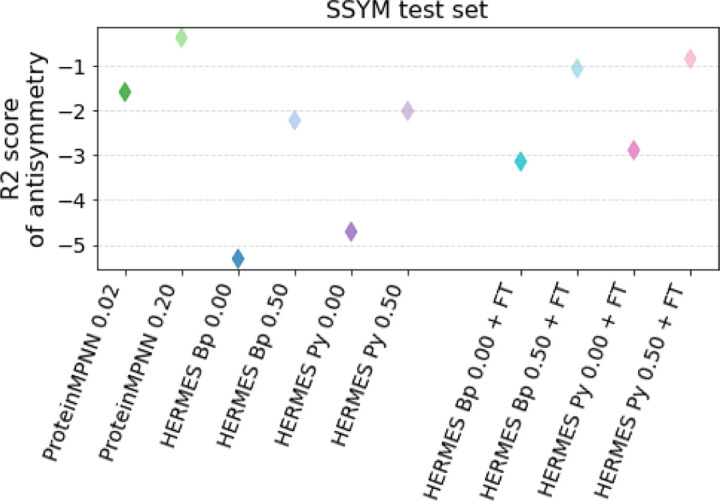
Anti-symmetry-respecting score for various models (higher is better). We compute the *R*^2^ between the predicted forward mutations and the predicted negative of reverse mutations for the Ssym dataset, which contains 352 variants from 19 structures (forward) and a structure for each of the reverse variants. We speculate that the higher performance of ProteinMPNN is due to it focusing less - and thus overfitting less - on specific atomic details of the environment (ProteinMPNN does not model side-chains, HERMES does).

We evaluate the respecting of the anti-symmetry condition in two ways. First, we consider the overall Pearson correlation on Ssym-direct vs. Ssym-reverse: a model that respects the anti-symmetry condition should perform equally well on both. We find that both ProteinMPNN and HERMES models exhibit a significantly smaller gap between the two correlations than RaSP, especially after fine-tuning ({0.64 ; 0.46} vs. {0.58 ; 0.18} on {direct ; reverse} for HERMES PR 0.50 + FT vs. RaSP + FT, Table S2). Second, we compute the *R*^2^ score between a model’s predictions on the *direct* dataset, and the negative of its predictions on the *reverse* dataset (Figure 4). We consistently find that models trained with noise have better anti-symmetry score, that fine-tuning improves the anti-symmetry score of HERMES models, and that ProteinMPNN models have the best anti-symmetry scores, even though its predictions are zero-shot and of lower overall accuracy. We speculate that this is because ProteinMPNN only considers the backbone atoms and amino-acid labels, unlike HERMES, which includes all atoms in the structure. Consequently, ProteinMPNN has a reduced risk of overfitting on spurious details of the atomic environments. Interestingly, we note that the scores on the *reverse* dataset are consistently under-shooting (Figure S1).

### C. Predicting mutational effect on binding, a.k.a. $\Delta \Delta G^{\text{binding}}$

The ΔΔG value associated with a binding event is slightly different from ΔΔG of stability, which is associated with a protein’s *folding* event. Specifically, for the case of stability, the free energy of folding $\Delta G^{\text{stability}}$ for both the wildtype and the mutant is computed from:

(4) $$\Delta G^{\text{stability}}=G^{\text{folded}}-G^{\text{unfolded}}$$

resulting in the stability effect of a mutation, $\Delta \Delta G_{\text{stability}}=\Delta G_{\text{mt}}^{\text{stability}}-\Delta G_{\text{wt}}^{\text{stability}}$. On the other hand, the free energy associate with binding follows,

(5) $$\Delta G^{\text{binding}}=G^{\text{bound}}-G^{\text{unbound}}$$

resulting in the binding effect if a mutation $\Delta \Delta G^{\text{binding}}=\Delta G_{\text{mt}}^{\text{binding}}-\Delta G_{\text{wt}}^{\text{binding}}$. Crucially, the *unbound* proteins are usually considered to be folded. In fact, $\Delta G^{\text{binding}}$ is usually experimentally determined starting from the measurements of the dissociation constant $K_{d}$:

(6) $$\Delta G^{\text{binding}}=RT\log K_{d}$$

where *T* is usually taken to be room temperature, as most experiments are performed at that temperature, and *R* = 8.314 J K^−1^mol^−1^ is the gas constant. Here, we present our predictions for the binding effect of mutations for a number of datasets.

#### SKEMPI v2.0 dataset [60].

This dataset, to our knowledge, is the most comprehensive dataset comprising the effect of mutations on the binding affinity of protein-protein interactions, with the associated crystal structures of the wildtype’s bound conformation. After filtering duplicate experiments, the dataset includes: 5,713 $\Delta \Delta G^{\text{binding}}$ values across 331 structures, of which 4,106 are single-point mutations across 308 structures. Further filtering for mutations that belong to structures with at least 10 mutations in the dataset, 116 structures remain with 5,025 total mutations; By restricting to only single-point mutations, we arrive at 93 structures and 3,485 mutations. We consider both “Per Structure” and “Overall” correlations, and will focus on single-point mutations. For multi-point mutations, we use an additive model and neglect epistasis.

**FIG. 5.**
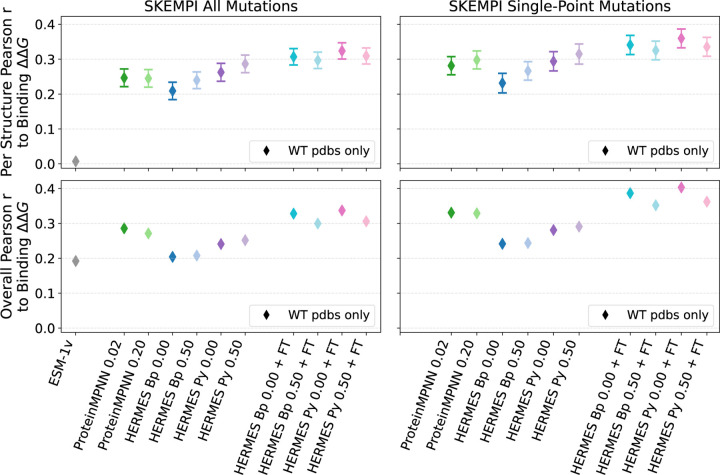
Performance of models at recovering Binding ∆∆*G* from the SKEMPI 2.0 dataset. “Per Structure” correlations are computed by averaging correlations across proteins with at least 10 mutations; ”Overall” correlations are instead computed across all mutations among all proteins. Performance of ESM-1v was taken from [21]. Error bars show the standard error. We do not show error bars for ESM-1v as we show previously-reported scores.

The best-performing model we consider is HERMES PR0.00 with fine-tuning, with Per Structure Pearson correlation of 0.32 ± 0.07 and Overall correlation of 0.34 (Figure 5 and Table S3). Training with noise does not seem to consistently provide a boost in performance. Fine-tuning on Rosetta stability ∆∆*G* values, instead, does. Notably, the predictive power of all models - even zero-shot models - is significantly lower here than it is for protein stability, indicating that the pre-training and fine-tuning strategies proposed here are more specific to modeling of protein stability than binding; perhaps a fine-tuning procedure specific to protein-protein interactions would provide benefit for this task.

We do not expect ESM-1v to have strong predictive power as its training on single-chain sequences does not easily transfer to reasoning over multi-chain interactions between protein. Structure based models using local atomic neighborhoods are agnostic to their provenance, as a neighborhood at the interface of two proteins (two portions of a single chain) are in principle indistinguishable from neighborhoods at the interface between two distinct chains (i.e., domains within a protein). Indeed, ESM-1v cannot predict the “per structure” binding effect of mutations (Per Structure correlation of 0.01), but it surprisingly has some predictive power, though less than all other models, across proteins (Overall correlation of 0.19). Finally, all models are better at predicting ΔΔG of single-point mutations, and seem to lack accuracy in the epistatic effects of multiple mutations.

#### ATLAS dataset of TCR-pMHC interactions [61].

**FIG. 6.**
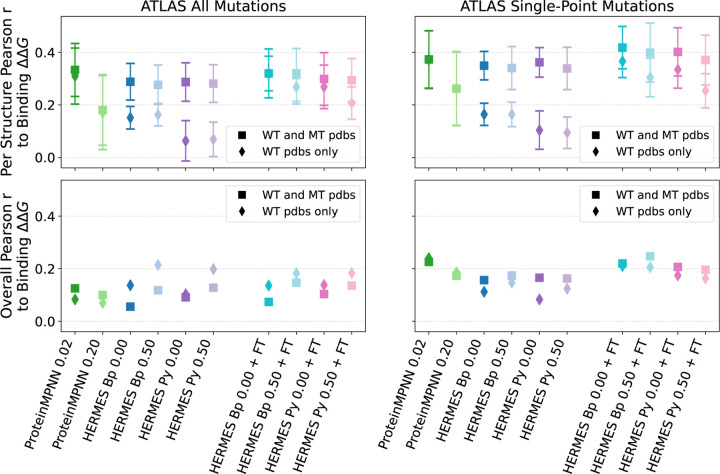
Performance of models at recovering Binding ∆∆*G* from the ATLAS dataset. “Per Structure” correlations are computed by averaging correlations across proteins with at least 10 mutations; “Overall” correlations are instead computed across all mutations among all proteins. We show both predictions made using the wildtype structure only (Equation 2) and with using mutant structures (Equation 1). Error bars show the standard error.

Similar to SKEMPI, this dataset gather the effect of mutations on the binding affinity of protein-protein interactions, with the associated crystal structures of the wildtype’s bound conformation. There are two main differences between ATLAS with SKEMPI: 1) ATLAS focuses on TCR-pMHC complexes, thoroughly annotating on which region of the complex the mutation is taking place, and 2) it provides mutant structures as well, where most are computationally modeled. After filtering duplicate experiments, the dataset includes: 372 ∆∆*G*^binding^ values across 39 structures, of which 261 are single-point mutations across 36 structures. Further filtering for mutations belonging to structures that have at least 10 mutations in the dataset, results in 8 remaining structures with 266 total mutations; 7 structures and 180 mutations if considering only single-point mutations. Therefore, this dataset is consistently smaller than SKEMPI. Pearson correlations for predictions of binding affinities in ATLAS are shown in Figure 6, Table S4 and Table S5. Not many consistent patterns can be identified, probably due to the small sample sizes. HERMES models seem to benefit from fine-tuning, and proteinMPNN does surprisingly well, outperforming even fine-tuned HERMES models in some settings.

### D. Prediction of Deep Mutational Scanning assays

We evaluate model performance on 25 out of the 41 Deep Mutational Scanning (DMS) studies collected by [26] and considered by [27]. To simplify the analysis, we consider only the 37 studies containing single-point mutations only. For these, only the proteins’ sequences were available to us a priori. Starting from the sequences, we augmented the dataset with both experimental structures that we identified in the RCSB website ^3^ and AlphaFold2 structures, either from the AlphaFold database ^4^, or folded using the AlphaFold2 [20] google colab with default parameters. Keeping only studies with at least one high-quality structure, we were left with 25 studies, many of which with only the AlphaFold-generated structure. Some proteins have multiple experimental structures, as in each structure they are bound to different a different and it was not obvious from the study of origin which ligand was more appropriate. We provide structures and detailed notes for each study on our github repository.

**FIG. 7.**
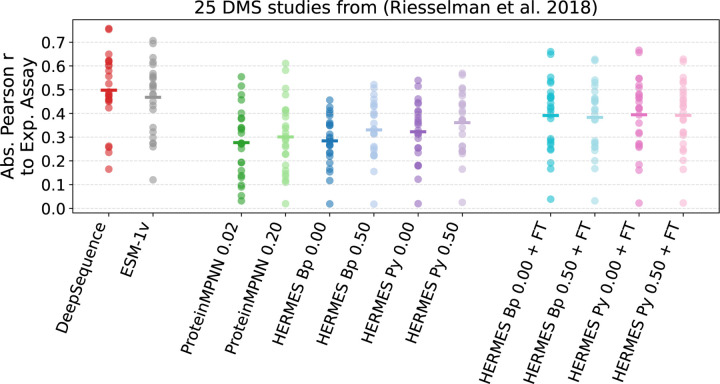
Performance of models at recovering values of DMS experimental assays from [26]. Each point is a study (single protein), and horizontal bars are mean values. Fine-tuning HERMES models on Rosetta stability ∆∆*G* values improves performance, but it does not enable them to reach the levels of state-of-the-art sequence-based models DeepSequence and ESM-1v.

In Figure 7 we show absolute Pearson correlations between model predictions and expreiments for the 25 studies, selected as described above. We use absolute Pearson correlation for simplicity, as assays may have either positive or negative sign associated with higher fitness. Patterns are similar to those we found for the stability effect of mutations ∆∆*G*: training with noise improves zero-shot models, and so does pre-processing with PyRosetta. Models fine-tuned on Rosetta stability ∆∆*G* see their performance improved. However, the best structure-based model (HERMES PR 0.50 + FT with mean Pearson r of 0.40) still performs significantly worse, on average, compared to the state-of-the-art sequence-based models (DeepSequence [26] with 0.50, and ESM-1v [27] with 0.47).

## IV. DISCUSSION

Here, we presented HERMES, an efficient deep learning method for inferring the effects of mutations on protein function, conditioned on the local atomic environment surrounding the mutated residue. HERMES is pre-trained to model amino-acid preferences in protein structures, and can be optionally fine-tuned on arbitrary mutation effects datasets. We provide HERMES models pre-trained on a large non-reduntant chunk of the protein structure universe, as well as the same models fine-tuned on stability effects (∆∆*G*) computed with Rosetta. We thoroughly benchmark HERMES against other state-of-the-art models, showing robust performance on a wide variety of proteins and functions: stability effects, binding affinity, and several deep mutational scanning assays. We open-source our code and data used for experiments, where we provide easy-to-use scripts to run HERMES models on desired protein structures and mutation effects, as well as code to fine-tune our pre-trained HERMES models on the user’s own mutation effect data.

## Supplementary Material

Supplement 1

## Figures and Tables

**TABLE I.** Performance of HERMES models on wildtype amino-acid classification on 40 CASP12 test proteins. As expected, models trained with noise have worse Cross Entropy (CE) Loss and Accuracy. Interestingly, models fine-tuned on Rosetta Stability ∆∆*G* values retain most of their accuracy (the highest-probability amino-acid is usually still the wildtype) but have significantly worse CE Loss, indicating a significant increase in the entropy of predictions.

	CE Loss	Accuracy
*HERMES BP 0.00*	0.69	0.76
*HERMES BP 0.50*	1.01	0.66
*HERMES PR 0.00*	0.78	0.73
*HERMES PR 0.50*	1.06	0.64
*HERMES BP 0.00 + FT*	2.68	0.71
*HERMES BP 0.50 + FT*	2.70	0.59
*HERMES PR 0.00 + FT*	2.69	0.69
*HERMES PR 0.50 + FT*	2.70	0.58

**TABLE II.** Pearson correlation with T4 Lysozyme stability ∆∆*G* values with and without the use of mutant crystal structures. Interestingly, HERMES fine-tuned models benefit from the use of mutant structures, whereas HERMES zero-shot models do not. ProteinMPNN zero-shot instead benefits from mutant structures. Note that these values are computed using 33 mutations.

	*WT only*	*WT and MT*
*ProteinMPNN 0.02*	0.65	**0.80**
*ProteinMPNN 0.20*	0.69	**0.77**
*HERMES BP 0.00*	**0.73**	0.69
*HERMES BP 0.50*	**0.79**	0.75
*HERMES PR 0.00*	**0.71**	0.70
*HERMES PR 0.50*	**0.82**	0.75
*HERMES BP 0.00 + FT*	0.79	**0.84**
*HERMES BP 0.50 + FT*	0.83	**0.87**
*HERMES PR 0.00 + FT*	0.80	**0.84**
*HERMES PR 0.50 + FT*	0.84	**0.85**

## Footnotes

1
https://github.com/StatPhysBio/protein_holography-web

2
https://colab.research.google.com/drive/1JQxkXeGZJYYcPNglN3rYUYiOuUOkwJPL

3
https://www.rcsb.org/

4
https://alphafold.ebi.ac.uk/
