# HERMES: Holographic Equivariant neuRal network model for Mutational Effect and Stability prediction

**DOI:** 10.1101/2024.07.09.602403

**Published:** 2024-10-02

**Authors:** Gian Marco Visani, Michael N. Pun, William Galvin, Eric Daniel, Kevin Borisiak, Utheri Wagura, Armita Nourmohammad

**Affiliations:** Paul G. Allen School of Computer Science and Engineering, University of Washington; Department of Physics, University of Washington; Paul G. Allen School of Computer Science and Engineering, University of Washington; Paul G. Allen School of Computer Science and Engineering, University of Washington; Department of Physics, University of Washington; Department of Physics, MIT; Department of Physics, Applied Math, and CSE, University of Washington, Fred Hutch Cancer Research Center, Seattle, WA

## Abstract

Predicting the stability and fitness effects of amino-acid mutations in proteins is a cornerstone of biological discovery and engineering. Various experimental techniques have been developed to measure mutational effects, providing us with extensive datasets across a diverse range of proteins. By training on these data, machine learning approaches have advanced significantly in predicting mutational effects. Here, we introduce HERMES, a 3D rotationally equivariant structure-based neural network model for mutation effect prediction. Pre-trained to predict amino-acid propensities from their surrounding 3D structure atomic environments, HERMES can be efficiently fine-tuned to predict mutational effects, thanks to its symmetry-aware parameterization of the output space. Benchmarking against other models demonstrates that HERMES often outperforms or matches their performance in predicting mutation effects on stability, binding, and fitness, using either computationally or experimentally resolved protein structures. HERMES offers a versatile suit of tools for evaluating mutation effects and can be easily fine-tuned for specific predictive objectives using our open-source code.

## 1 Introduction and Related Work

Understanding the effects of amino acid mutations on a protein’s function is a hallmark of biological discovery and engineering. Identifying disease-causing mutations [1, 2], enhancing enzymes’ catalytic activity [3, 4], forecasting viral escape [5, 6, 7], and engineering high-affinity antibodies [8], are just some of the areas of study that rely on accurate modeling of mutational effects. Effects on protein stability are likely the most studied, as sufficient stability is usually a prerequisite of the protein’s successful carrying of its function [9]. Understanding the impact of mutations on the protein’s *binding affinity* to its partner is also crucial, as most functions are mediated by binding events. These effects can be accurately measured experimentally, for example via thermal or chemical denaturation assays [10], by surface plasmon resonance [11], and, more recently, by Deep Mutational Scanning (DMS) [12, 13, 14]. These experiments are laborious and with limited throughput.

Computational modeling of mutational effects remains an attractive alternative to costly experiments. Methods based on molecular dynamics simulations are accurate for short-time (nano seconds) protein responses but are limited in predicting substantial changes in protein often inflicted by amino acid mutations [15]. Models using physical energy functions such as FoldX [16] and Rosetta [17] are well-established and remain widely used for predicting the stability effect of mutations, though they often lack accuracy and are slow [2]. Recently, machine learning models have shown substantial progress in this domain. Sequence-based [18, 19] or structure-based [20, 21, 22, 2, 23, 24], approaches are used to predict the propensity of amino acids, and by extension, the effect of mutations in a protein [18, 19, 20]. These pre-trained models serve as robust baselines, upon which additional fine-tuning on smaller protein stability datasets can significantly enhance the accuracy of predictions for mutational effects [2, 22].

Here, we introduce HERMES, which is built upon a self-supervised structure-based model H-CNN [20], and fine-tuned to predict mutational effects in proteins. Similar to H-CNN, HERMES has a 3D rotationally equivariant architecture, but with an improved performance. During pre-training, HERMES is trained to predict a residue’s amino acid identity from its surrounding atomic neighborhood within a 10 Å radius in the 3D structure. To fine-tune HERMES for mutational effects, we take the pre-trained model’s logits corresponding to the amino acid pair of interest, and train a model to match the experimental data for the functional difference between them. With our parametrization, HERMES automatically respects the permutational anti-symmetry in the mutational effects, which other models achieve through data augmentation [22]. We extensively benchmarked HERMES across various datasets, demonstrating state-of-the-art performance in predicting stability and highly competitive results in predicting binding effect of mutations, even when using computationally resolved protein structures. Our code is open source at https://github.com/StatPhysBio/hermes/tree/main, and allows users to both run the models presented in this paper, and easily fine-tune HERMES on their data.

## 2 Methods

**Figure 1:**
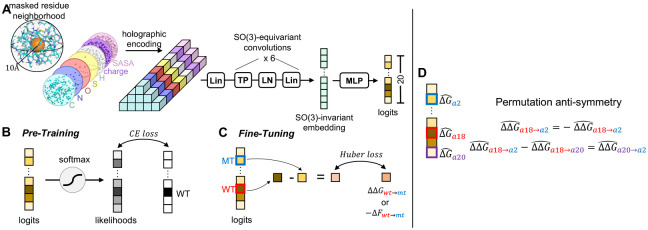
Schematic of HERMES. **(A)** Model architecture. We refer the reader to [25, 26] for details. **(B)** Pre-training procedure. We train HERMES to predict the identity of the central neighborhood’s amino-acid, whose atoms have been masked. **(C)** Fine-tuning procedure over mutation effects. We simply fine-tune HERMES to make the difference of logits for two amino-acids regress over the corresponding mutation’s score. **(D)** Our fine-tuning procedure makes the 20 logits values effectively converge to predicted Δ*G* (or, more broadly, *F*) values, up to a site-specific constant. This ensures that permutation anti-symmetry is respected without the need for data augmentation. This symmetry is however only approximate, as the output is conditioned on a neighborhood bearing the signature of the wildtype amino-acid.

HERMES is trained in two steps (Figure 1). First, following [20], we train an improved version of the model Holographic Convolutional Neural Network (H-CNN) to predict the identity of an amino acid from its surrounding structural neighborhood. Specifically, we remove (mask) all atoms associated with the focal residue and predict its identity using all atoms within 10 Åof the the focal residue’s C-*α* (Figure 1B). Second, we develop a procedure to fine-tune HERMES on mutation effects Δ*F* in general, with a specific focus on predicting the stability effect of mutations ΔΔ*G* (Figure 1).

### Preprocessing of protein structures.

To pre-process the protein structure data, we devise two distinct pipelines, relying either on (i) Pyrosetta [27], or (ii) Biopython [28] and other open source tools with the code adapted from [2]; see Section A.1.1 for details. The Pyrosetta pipeline is considerably faster, but requires a license, whereas the Biopython pipeline is open-source. We train models using both pipelines. Pipelines used at inference and training must match. Differences in results between the two pipelines are minimal (see Figs. S2, S5 and Table S1); for our main analyses, we report only the results using Pyrosetta.

### HERMES architecture and pre-training.

Similar to H-CNN, HERMES has a 3D rotationally equivariant architecture, with comparable number of parameters (~3.5M), but with a ~2.75× improved speed in its forward pass, and a higher accuracy (Figure 1A). In short, atomic neighborhoods - i.e., featurized point clouds - are first projected onto the orthonormal Zernike Fourier basis, centered at the (masked) central residue’s C-*α*. We term the resulting Fourier encoding of the data an *holographic encoding*, as it presents a superposition of 3D spherical holograms [20]. Then, the resulting *holograms* are fed to a stack of SO(3)-Equivariant layers, which convert the holograms to an SO(3)-invariant embedding - i.e. a representation that is invariant to 3D rotations about the center of the initial holographic projection. These embeddings are then passed through an MLP to generate the desired predictions. Each HERMES model is an ensemble of 10 individually-trained architectures. We trained versions of HERMES after adding Gaussian noise to the 3D coordinates, with standard deviation 0.5 Å, and different random seeds for each of the 10 models. We refer the reader to [26, 25] for further details on the architecture, and the mathematical introduction to SO(3)-equivariant models in Fourier space. We implement HERMES using e3nn [29].

We pre-train HERMES on neighborhoods from protein chains in ProteinNet’s CASP12 set with 30% similarity cutoff [30], and featurize atomic neighborhoods using atom type - including computationally-added hydrogens - partial charge, and Solvent Accessible Surface Area.

### Predicting fitness effect of mutations with HERMES.

HERMES can be seen as a generative model for amino-acid labels for a residue, conditioned on the atomic environment surrounding the residue. Conditional generative models of amino-acid labels are shown to successfully make zero-shot predictions for mutational effects [18, 19, 20]. The log-likelihood difference between the original amino acid (often wildtype) *aa*_0_ and the mutant *aa*_1_ at a given residue *i*, conditioned on the surrounding neighborhood *X*_*i*_, can well approximate mutational effects

(1) $$F_{1}-F_{0}\propto \text{log}\;P\left(aa_{1}|X_{i}^{\left(1\right)}\right)-\text{log}\;P\left(aa_{0}|X_{i}^{\left(0\right)}\right)$$

The superscripts on *X*_*i*_ indicate the structure from which the atomic neighborhood is extracted, highlighting that mutations at a residue can reorganize the surrounding structural neighborhood. Computational tools like Rosetta [27] can be used to relax the structural neighborhoods subject to mutations, when the mutant structure is not available [20]. However, this procedure can be inaccurate and computationally expensive. For practical use of HERMES, we only consider the use of a single (often the original wildtype) structure to predict all possible variant effects, approximating eq. 1 by

(2) $${\hat{F}}_{1}-{\hat{F}}_{0}\propto \text{log}\;P\left(aa_{1}|X_{i}^{\left(0\right)}\right)-\text{log}\;P\left(aa_{0}|X_{i}^{\left(0\right)}\right)=L_{\text{mt}|X_{i}^{\left(0\right)}}-L_{\text{wt}|X_{i}^{\left(0\right)}}$$

where $L_{aa|X_{i}^{\left(0\right)}}$ is the logit associated with the indicated amino acid, conditioned on the surrounding neighborhood for the initial amino acid *aa*_0_ (e.g. wildtype). A similar approach was taken by [22].

### Fine-tuning on mutation effects.

We fine-tune pre-trained HERMES models on mutation effects, similar to prior work [2, 22]. However, unlike those works which train a separate regression head using as input embeddings from the pre-trained model, we simply fine-tune the model itself to make the predicted logit differences in eq. 2 regress over mutation effects (Figure 1C); see Section A.1.2 for details. We fine-tune HERMES on several datasets, as reported in the Results section.

### Permutational anti-symmetry for mutation effects.

The thermodynamic changes in the stability of a protein ΔΔ*G*_*aa*0→*aa*1_ by a mutation *aa*_0_ → *aa*_1_ is simply equal to the difference between the free energy of the mutant structure Δ*G*_*aa*1_ and that of the original structure Δ*G*_*aa*0_. Thus, the back mutation should have the opposite effect on the stability ΔΔ*G*_*aa*1→*aa*0_ = −ΔΔ*G*_*aa*0→*aa*1_; a similar property occurs when considering triplets of amino acids ΔΔ*G*_*aa*1→*aa*0_ = ΔΔ*G*_*aa*2→*aa*0_ −ΔΔ*G*_*aa*2→*aa*1_. The same anti-symmetric effects are present for the effect of mutations on protein fitness. HERMES is parametrized to automatically account for the anti-symmetric nature of mutations by learning a score for each of the 20 amino acids at a given site, which is associated to their fitness or thermodynamic free energy contribution, up to a site specific constant (Figure 1D). This is in contrast to other popular methods for mutation effect predictions [22, 2]. For example, Stability-Oracle only achieves this anti-symmetric property through data augmentation, by training on all the 380 possible amino acid pairs at each site, resulting in dataset augmented from 117k to 2.2M examples [22].

## 3 Predicting stability effect of mutations

We evaluate the performance of HERMES on datasets used by RaSP [2] and Stability-Oracle [22]. RaSP was fine-tuned on stability effects computed with Rosetta [27] for 35 protein structures, then tested on Rosetta-computed stability effects for 10 other proteins, as well as on experimentally determined stability effects; we indicate models fine-tuned on this data by “+ Ros”. Stability-Oracle was trained on a curated dataset of experimentally measured stability effects termed “cDNA117K”, and tested on a dataset termed “T2837”; we indicate models fine-tuned on this data by “+ cDNA117K”.

**Figure 2:**
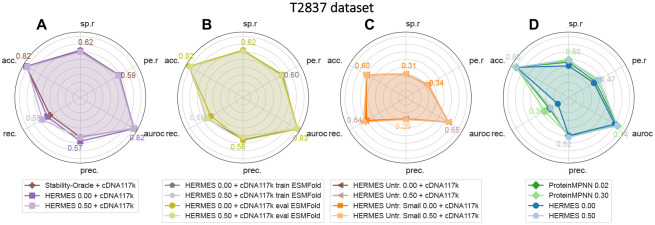
Predicting stability effect of mutations in T2837 dataset. The Pearson correlation (pe.r), Spearmann correlation (sp.r), accuracy (acc.), recall (rec.), precision (prec.), and AUROC are shown for different models. (A) When fine-tuned on the same dataset, HERMES models perform equivalently to the Stability-Oracle [22]. (B) HERMES performance does not change even when trained on ESMFold-predicted structures and evaluated on crystal structures, and vice-versa. (C) Non-pre-trained HERMES models perform the worst, and reducing their size from 3.5M to 50k parameters does not improve performance. (D) Models without fine-tuning show decent performance. Adding noise to structures during training consistently enhances their performance, though this effect is not observed in fine-tuned models.

HERMES achieves state-of-the-art performance compared to RaSP (Figs. S1,S2) and Stability-Oracle (Figs. 2A,S4A,S3), using the same fine-tuning data, and without any data augmentation. Moreover, HERMES’ predictions are robust to the use of structures computationally resolved by ESMFold [31] at either training or testing time (Figure 2B). Our results also indicate that pre-training on the wildtype amino-acid classification task provides significant help for downstream stability predictions. Notably, models that are pre-trained only, without any fine-tuning, perform significantly better than models trained solely on mutation effects without pre-training (Figs. 2C, 2D). In fact, we could not prevent overfitting in the non-pre-trained HERMES model, even after significantly reducing the model size from 3.5M to 50k parameters (Figure 2C).

Note that HERMES uses only the starting structure to predict mutational effects (eq. 2), and thus, its prediction are only approximately permutation anti-symmetric with respect to mutations. Specifically, the predicted effect of a forward mutation, using the initial structure, is only approximately negative of the effect of the reverse mutation, using the final structure. To assess the extent of deviation from anti-symmetry resulting from our approximation, we use the Ssym dataset, which includes measurements for the stability effect of 352 mutations across 19 different proteins structures, together with the experimentally-determined structures of all of the 352 mutants[32].

**Figure 3:**
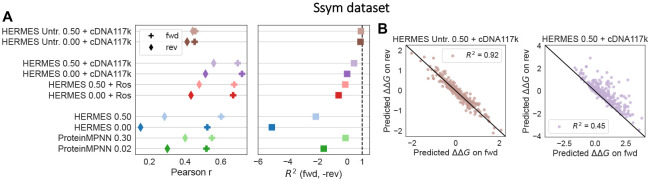
Permutational anti-symmetry of stability effect of mutations. **(A)** Pearson correlation between the measured stability effects of mutations from the Ssym dataset and the predictions on the forward and reverse mutations are shown (left). The effects of reverse mutations are computed using the mutant structures. The *R*^2^ between the forward and (negative) reverse predictions is shown, with higher values indicating more respect for Permutational anti-symmetry (right). **(B)** Models with pre-training (right) tend to predict a larger magnitude of stability effect for forward mutations compared to reverse mutations, compared to non-pre-trained models (left).

We find that HERMES models, as well as ProteinMPNN, consistently predict the stability effect of mutations in the “forward” direction (from wildtype) more accurately than in the reverse direction (Figure 3A). Although none of the Ssym structures were included in our training data, we hypothesize that this effect arises from our models being pre-trained to classify amino acids in wild-type structures, some of which may be homologues to the Ssym structures. Indeed, we observe that removing the pre-training step lessens the discrepancy between forward and reverse predictions, though this comes at the cost of reduced accuracies for both cases (Figure 3A; brown points). Moreover, HERMES models with pre-training tend to predict a larger magnitude of stability effect for forward mutations compared to reverse mutations, further underscoring the bias of these models toward wildtype structures (Figure 3B). Adding noise during training partly mitigates the bias, as it can reduce the model’s wildtype preferences (Table S1).

## 4 Predicting binding effect of mutations

We tested the accuracy of HERMES on predicting the binding effect of mutations on the SKEMPI v2.0 dataset [33], which, to our knowledge, is the most comprehensive dataset comprising mutationl effects on protein-protein binding interactions, with the associated crystal structures of the wildtype’s bound complex. We evaluate pre-trained-only ProteinMPNN and HERMES models, as well as HERMES models fine-tuned on stability changes, on predicting binding affinity changes on the wild-type bound structures. Furthermore, for single-point-mutations only, we fine-tune HERMES models on SKEMPI itself using a 3-fold cross-validation scheme, thus ensuring that every point of SKEMPI is evaluated upon. Using structural homology, we provide three splitting strategies with increasing levels of difficulty; see Section A.1.5 for details.

**Figure 4:**
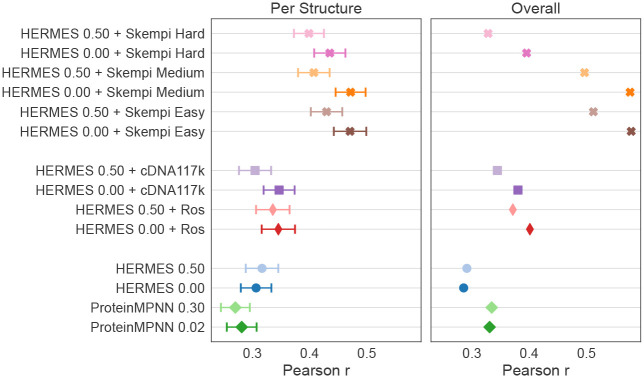
Single-point mutational effects on binding affinity from SKEMPI. Averaged “per-structure” and “overall” Pearson correlations between the predicted binding effect of mutations and the measurements from the SKEMPI data are shown, for non-fine-tuned models (bottom), models fine-tuned for stability prediction (middle), and models fine-tuned on the SKEMPI data (top).

Following [21], we report the accuracy of our predictions both across mutations within each structure individually (“per-structure” correlations), and across mutations pooled from all structure (“overall” correlations). Note that “per-structure” accuracy is particularly relevant when optimizing the binding of a specific protein to its target. As shown in Fig. 4 and Table S2, pre-trained-only models demonstrate some predictive power, and fine-tuning on stability effects enhances the accuracy of binding effect predictions, confirming that transfer learning can be leveraged between the two tasks. Fine-tuning directly on the SKEMPI dataset offers even greater improvements, achieving state-of-the-art performance for “Per-Structure” analysis and competitive results for “Overall” analysis (Table S2).

## 5 Discussion

Here, we presented HERMES, an efficient deep learning method for inferring the effects of mutations on protein function, conditioned on the local atomic environment surrounding the mutated residue. HERMES is pre-trained to model amino-acid preferences in protein structures, and can be optionally fine-tuned on arbitrary mutation effects datasets. We provide HERMES models pre-trained on a large non-reduntant chunk of the protein structure universe, as well as the same models fine-tuned on stability and binding effects of mutations. We thoroughly benchmark HERMES against other state-of-the-art models, showing robust performance on a wide variety of proteins and functions: stability effects, binding affinity, and several deep mutational scanning assays. We open-source our code and data used for experiments, where we provide easy-to-use scripts to run HERMES models on desired protein structures and mutation effects, as well as code to fine-tune our pre-trained HERMES models on the user’s own mutation effect data.

## Supplementary Material

1
