# HERMES: Holographic Equivariant neuRal network model for Mutational Effect and Stability prediction

**DOI:** 10.1101/2024.07.09.602403

**Published:** 2026-01-15

**Authors:** Gian Marco Visani, William Galvin, Zac Jones, Michael N. Pun, Eric Daniel, Kevin Borisiak, Utheri Wagura, Armita Nourmohammad

**Affiliations:** 1 Paul G. Allen School of Computer Science and Engineering, University of Washington, Seattle, WA, USA.; 2 Department of Biochemistry, University of Washington, Seattle, WA, USA.; 3 Institute for Protein Design, Seattle, WA, USA.; 4 Department of Physics, University of Washington, Seattle, WA, USA.; 5 Department of Physics, Massachusetts Institute of Technology, Cambridge, MA, USA.; 6 Department of Applied Mathematics, University of Washington, Seattle, WA, USA.; 7 Fred Hutchinson Cancer Center, Seattle, WA, USA.; 8 Yale Center for Systems and Engineering Immunology, Yale UniversityNew Haven, CT, USA.; 9 Departments of Immunobiology and Biomedical Engineering, Yale University, New Haven, CT, USA.

**Keywords:** Machine Learning, Protein Design, Mutation Effect Prediction, Thermodynamic Stability

## Abstract

Accurately predicting how amino acid substitutions alter protein function is a central challenge in biology, with applications from interpreting disease variants to engineering vaccines and therapeutic proteins. We introduce HERMES, a family of fast, structure-based models that predict mutational effects from the local three-dimensional atomic environment around each residue. Pre-trained on the masked amino-acid prediction task, HERMES shows strong zero-shot performance for predicting changes in thermodynamic stability and protein–protein binding affinity. We find that this pre-training induces a bias toward substitutions with similar size to the wild-type. To address this, we develop an amortized fine-tuning strategy that incorporates packing flexibility, substantially reducing size-based bias while preserving sensitivity to mutational effects. We demonstrate that HERMES can then be fine-tuned on experimental measurements without adding parameters or relying on costly data augmentation, achieving performance competitive with state-of-the-art stability predictors. Finally, we show that HERMES identifies antigen-stabilizing mutations across multiple viral envelope proteins, enabling computationally efficient, structure-guided vaccine design. Together, these results establish HERMES as a practical and accurate framework for structure-based mutational effect prediction.

## Introduction

Understanding the effects of amino acid substitutions on protein function is fundamental to biological discovery and engineering, with applications spanning disease variant identification [1, 2], enzyme optimization and engineering [3, 4], viral escape prediction [5–7], antibody engineering [8], and vaccine antigen stabilization [9–13].

Mutational effects on thermodynamic stability and binding affinity are particularly well-studied, as stability is typically prerequisite for function [14] and most biological processes are mediated by binding events. While these effects can be measured experimentally via denaturation assays [15], surface plasmon resonance [16], or deep mutational scanning [17–19], such experiments remain laborious despite recent throughput improvements [20].

Computational approaches offer an alternative. Molecular dynamics simulations can accurately capture short-time (nano seconds) responses, but are limited in predicting substantial conformational changes often induced by mutations [21]. Physics-based energy functions, including FoldX [22] and Rosetta [23], remain widely used to predict mutation-induced stability changes [10], yet are often slow and inaccurate [2]. In recent years, machine learning models have shown considerable progress in this area: models trained to predict amino acid propensities at a given residue from surrounding sequence [24, 25] or structural context [2, 26–30] can approximate mutational effects across phenotypes.

Recent work improves these pre-trained baselines by modifying the pre-training objective [31, 32], and by fine-tuning to predict phenotype-specific mutational effects [2, 27, 30, 33], with thermodynamic stability as a frequent target for structure-based models [2, 27, 30]. Notable examples include RaSP [2], which fine-tunes a 3D convolutional neural network (CNN) on Rosetta-computed ΔΔG values [34]; Stability-Oracle [27], a graph transformer fine-tuned on experimental stability measurement from the Megascale dataset [20]; and ThermoMPNN [30], which fine-tunes the inverse folding model ProteinMPNN [35] on a different subset of Megascale.

Here, we present HERMES, a family of structure-based models built upon our previous H-CNN architecture [26]. Like H-CNN, HERMES employs a 3D rotationally equivariant, all-atom CNN architecture, but incorporates implementation improvements yielding a ∼ 2.75× speedup and an adaptable architecture enabling fine-tuning for arbitrary downstream tasks. We first pre-train HERMES on an inverse folding objective (i.e., predicting a residue’s amino acid identity from its surrounding atomic neighborhood within a 10 Å radius), then fine-tune for predicting mutational effects. The HERMES family comprises three model variants that differ in their treatment of structural context: HERMES-*fixed*, which holds the local environment static during prediction; HERMES-*relaxed*, which enables local structure relaxation through explicit side-chain repacking; and HERMES-*amortized*, which implicitly encodes the structural flexibility associated with different substitutions through amortization. We systematically analyze the biases of these models with respect to the physicochemical properties of mutating residues and characterize their utility across different tasks.

On thermodynamic stability benchmarks, we show that fine-tuned HERMES models match or exceed state-of-the-art performance. However, we identify a “wild-type preference bias” introduced by the pre-training objective that is only partially eliminated by fine-tuning. We also demonstrate HERMES’ utility for structure-based vaccine design, where stabilizing viral envelope glycoproteins in their metastable pre-fusion conformation is essential for presenting neutralizing antibody epitopes [9]. Identifying stabilizing mutations traditionally requires domain expertise and costly experimental iteration. While computational approaches using Rosetta [10, 13] or machine learning (e.g., ReCAP [11]) have emerged, HERMES offers significant computational efficiency over Rosetta and, unlike ReCAP, is publicly available. When evaluated on 33 known stabilizing mutations across 5 viral envelopes, HERMES ranks 19 within the top 3 predicted substitutions at each position, with particularly strong performance on mutations that stabilize independently without synergistic interactions. Lastly, we demonstrate that HERMES can be fine-tuned to predict mutational effects on protein-protein binding affinity, benchmarking competitively against existing models.

Our code is open source at https://github.com/StatPhysBio/hermes/tree/main, and allows users to both run the models presented in this paper, and easily fine-tune HERMES models on their data.

### Model

#### HERMES architecture and pre-training on masked amino acid classification.

**Figure 1:**
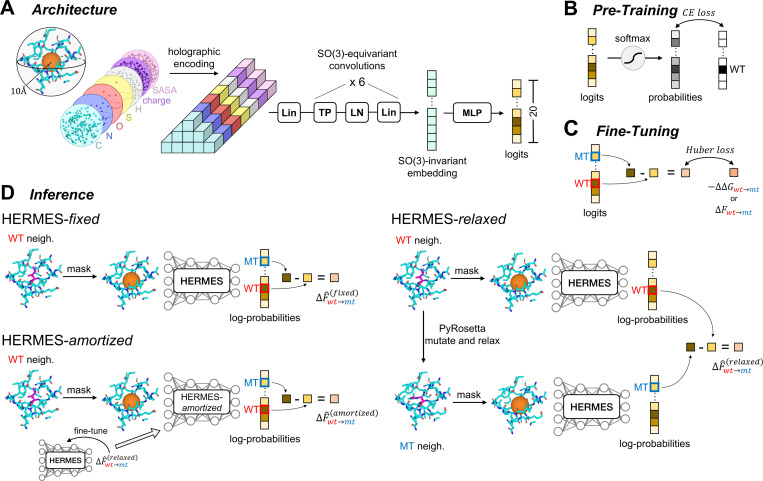
Overview of HERMES. **(A)** Model architecture: HERMES takes as input an all-atom structural neighborhood (10 Å radius) around a masked focal residue. Each atom is represented by its 3D coordinates, element type, partial charge, and solvent-accessible surface area (SASA). The neighborhood is projected onto a Zernike Fourier basis (spherical hologram) and processed by rotation (SO(3)) equivariant layers to produce a rotation-invariant embedding, which is mapped to 20 amino-acid-specific logits (Methods).**(B)** Pre-training: models are trained to predict the identity of the masked focal residue from its surrounding atomic neighborhood; logits in (A) are converted to amino-acid propensities (probabilities) via a softmax. **(C)** Fine-tuning for mutational effects: model weights are optimized to regress the logit difference between mutant and wild-type amino acids to the corresponding experimental mutational effect. **(D)** Inference protocols. HERMES-*fixed* scores a substitution as the difference between the the mutant and wild-type amino-acids logits from a single forward pass, conditioned on the masked wild-type neighborhood Xwt. HERMES-*relaxed* conditions the mutant term on an approximate mutant neighborhood ${\hat{X}}_{\text{wt}\to \text{mt}}$ generated in-silico by introducing the mutation on the wild-type structure and locally relaxing the structure with Rosetta [34]. HERMES-*amortized* distills the relaxed protocol by fine-tuning on HERMES-*relaxed* predictions, enabling fast fixed-style inference while retaining relaxation-aware behavior.

HERMES is a 3D, rotationally equivariant convolutional neural network that predicts the propensity of the 20 different canonical amino acids at a masked (removed) focal residue, given its local atomic neighborhood within the protein structure (Fig. 1A). Building on our prior atomistic model H-CNN [26], HERMES achieves faster inference and supports task-specific fine-tuning (Methods). Atomic neighborhoods are featurized by atom type (including added hydrogens), partial charge, and solvent-accessible surface area, and then projected onto an orthonormal Zernike Fourier basis centered at the masked C-*α* to form a *holographic encoding*. SO(3)-equivariant layers of the HERMES neural network map this encoding to a rotation-invariant embedding, which a final MLP converts into amino-acid propensities; additional details on Fourier-space SO(3) models are provided in the Methods and in [36, 37].

For pre-training, we train HERMES to recover the identity of the masked residues on ProteinNet’s CASP12 set, filtered at 30% sequence identity [38] (Fig. 1B). Each model is an ensemble of 10 independently trained networks (3.5M parameters each), with predictions averaged at inference. To improve robustness for zero-shot applications, we additionally train models with 0.50 Å coordinate noise. Pre-training performance is summarized in Table S1.

#### Zero-shot prediction of mutational effects with pre-trained HERMES.

The pre-trained HERMES model outputs $P\left(aa|X_{i,aa}\right)$, the probability of assigning amino acid *aa* at residue i, given the surrounding atomic environment (neighborhood) $X_{i,aa}$ in the structure. Crucially, $X_{i,aa}$ depends on the *aa*: the neighborhood geometry has the fingerprint of the masked amino acid during pre-training.

Conditional models are widely used for zero-shot prediction of mutational effects on protein function (e.g., [24–26]). We approximate the effect of wild-type to mutant (wt → mt) substitution at residue i by the log-likelihood ratio between the wild-type and the mutant amino acids, conditioned on the respective local atomic neighborhoods $X_{i,\text{wt}}$ and $X_{i,\text{mt}}$ (omitting the residue index i for clarity):

(1) $$\text{Δ}{\hat{F}}_{\text{wt}\to \text{mt}}\propto \log P\left(\text{mt}|X_{\text{mt}}\right)-\log P\left(\text{wt}|X_{\text{wt}}\right)$$

Here, the neighborhood X depends on the identity of the original amino acid (wt or mt), suggesting the potential need for having access to mutant structures for such predictions. The $\hat{.}$ (hat) indicates the model-predicted mutational effects, as opposed to experimental measurements (no hat).

When a mutant structure is unavailable, neighborhoods can be relaxed *in silico* (e.g., by Rosetta [34]), though this procedure is computationally expensive. A common alternative is to score all mutations using a single (typically wild-type) structure [26, 27, 35]. Here, we consider both of these protocols: **HERMES-*fixed*** evaluates mutational effects using the wild-type structure only (for both mt and wt propensities), while **HERMES-*relaxed*** evaluates the propensity of the mutant amino acid in the Rosetta-relaxed mt neighborhood ${\hat{X}}_{\text{wt}\to \text{mt}}$ starting from the available wt structure (Fig. 1D); see Methods for details. The resulting estimates for mutational effects from these two approaches follow,

(2) $$\begin{array}{l} \text{Δ}{\hat{F}}_{\text{wt}\to \text{mt}}^{\left(\text{HERMES}-fixed\right)}\propto \log P\left(\text{mt}|X_{\text{wt}}\right)-\log P\left(\text{wt}|X_{\text{wt}}\right)\\ \text{Δ}{\hat{F}}_{\text{wt}\to \text{mt}}^{\left(\text{HERMES}-relaxed\right)}\propto \log P\left(\text{mt}|{\hat{X}}_{\text{wt}\to \text{mt}}\right)-\log P\left(\text{wt}|X_{\text{wt}}\right). \end{array}$$

#### Fine-tuning HERMES to predict protein function.

Context-conditioned amino-acid likelihoods provide useful zero-shot proxies for mutational effects across diverse functions [24–26], but supervised models for specific functions can perform better in practice. Building on prior work [2, 27], we fine-tune HERMES models directly on mutational effect data. Unlike approaches that train a separate regression head [2, 27, 30]), we update the model end-to-end so that its *predicted scores* themselves align with measurements. Specifically, as shown in Fig. 1C, we make the predicted HERMES-*fixed $\text{Δ}{\hat{F}}_{\text{wt}\to \text{mt}}^{\left(\text{HERMES}-fixed\right)}$* (eq. 2) regress over the experimentally measured mutational effects $\text{Δ}F_{\text{wt}\to \text{mt}}$ by minimizing a robust Huber loss,

(3) $$L=\mathrm{HuberLoss}\left(\text{Δ}{\hat{F}}_{\text{wt}\to \text{mt}}^{\left(\text{HERMES}-fixed\right)},\text{Δ}F_{\text{wt}\to \text{mt}}\right)$$

which stabilizes training under outliers while calibrating predictions to the function of interest; see Methods for details.

#### Hermes-*amortized* to encode structural flexibility in HERMES-*fixed* by amortization.

Because the structural-relaxation step in HERMES-*relaxed* is computationally costly (∼ 66 times slower than HERMES-*fixed* on a single CPU / A40 GPU, Table S2), we distill HERMES-*relaxed* predictions into the model via a fine-tuning procedure. Concretely, we fine-tune the model so that the mutational-effect predictions produced with the fast HERMES-*fixed* protocol regress to the corresponding HERMES-*relaxed* predictions via Eq. 3 (Fig. 1D). We perform this fine-tuning on a small subset of neighborhoods extracted from the pre-training proteins (∼15k neighborhoods, 0.5% of the total). The resulting amortized model HERMES-*amortized* runs at HERMES-*fixed* speed (effectively the same protocol, just different model weights) yet closely matches HERMES-*relaxed* performance (Fig. S1).

## Results

### Predicting mutational effects on thermodynamic fold-stability

The chief task we evaluated the HERMES models on was to predict mutational effects on thermodynamic folding stability. Thermodynamic folding stability is defined as the change in Gibbs Free ΔG energy upon folding. Thus, the effect of a mutation wt → mt on folding stability is denoted by $\text{ΔΔ}G_{\text{wt}\to \text{mt}}=\text{Δ}G_{\text{mt}}-\text{Δ}G_{\text{wt}}$.

#### Training and test Data.

To enable a direct comparison with recent structure-based stability predictors, we fine-tuned and evaluated HERMES on the same benchmark splits used by RaSP [2], Stability-Oracle [27], and ThermoMPNN [30]. Specifically, we (i) trained on the RaSP Rosetta-derived stability dataset and tested on the experimental benchmark used in RaSP, (ii) used Stability-Oracle’s curated cDNA117k training set and T2837 test set; training on 117k ΔΔG values from ref. [20] filtered by enforcing ≤ 30% sequence identity to the test set, and (iii) trained on ThermoMPNN’s Megascale *train* split and evaluated on its Megascale *test* split, both from ref. [20]; training on 216k and testing on 28k mutation effects with 25% sequence-identity cutoff. Because these splits are defined by the original studies, our results are directly comparable across methods. We additionally note that the Megascale *train* split is not de-duplicated against T2837, and six of the T2837 proteins (∼ 5%) have > 90% sequence-similar homologs in the Megascale training set; see Methods for more details on these training and test sets.

#### Noise and side-chain relaxation improve zero-shot model predictions.

**Figure 2:**
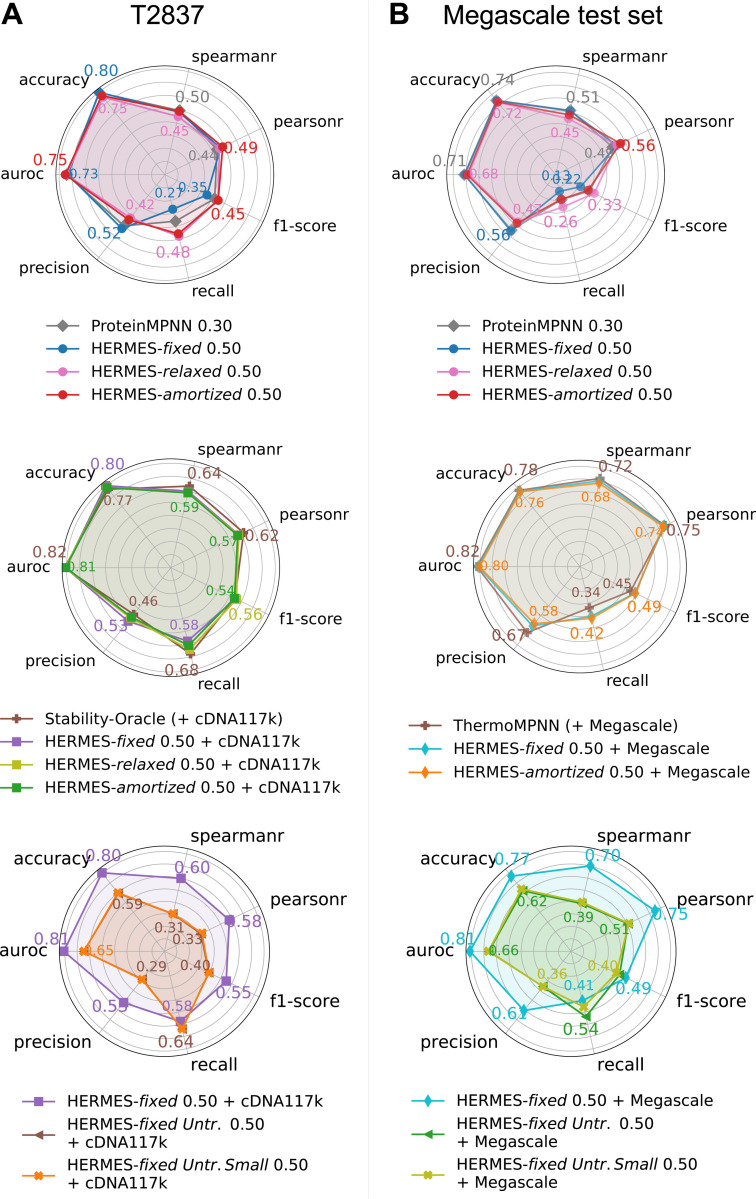
Predicting mutational effects on thermodynamic folding stability. Stabilizing-versus-destabilizing classification metrics are computed using ΔΔG<0 (experimental) and Δlogp>0 (predicted) as cutoffs for stabilizing mutations. **(A)** T2837 results: zero-shot models (top) and models fine-tuned or only trained on cDNA117k (middle and bottom). **(B)** Megascale test set results: zero-shot models (top) and models fine-tuned or only trained on the Megascale training set (middle and bottom). Model names indicate the architecture, the coordinate-noise amplitude used, and when applicable, the fine-tuning dataset (listed after “+”); *Untr.* is short for *Untrained*, indicating models that had no pre-training and were instead only trained on stability effects. Only models trained with coordinate noise are shown; the noise amplitude is indicated within each model name as standard deviation in Å units. Results for models trained without noise are provided in Fig. S2.

We first evaluated the *zero-shot* HERMES models (no fine-tuning on experimental data) on the T2837 and Megascale test sets (Fig. 2, S2). Pre-training on structures with Gaussian coordinate noise (0.5 Å s.d.) improves performance, consistent with prior work [26, 35]. Relative to HERMES-*fixed*, the packing-aware HERMES-*relaxed* achieves significantly higher recall (0.48 vs. 0.27; p-value < 0.01), with only a slight loss in precision, leading to a higher overall F1-score. HERMES-*amortized* performs similarly to HERMES-*relaxed*: on T2837, and on Megascale, it shows slightly lower recall and F1 while still outperforming HERMES-*fixed*; the p-values for the significance of these performance differences are reported in Fig. S5.

**Figure 3:**
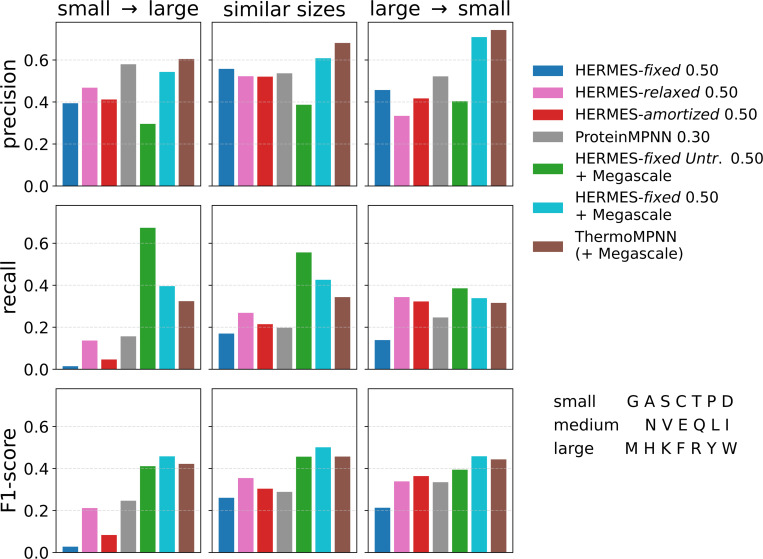
Impact of amino acid size changes on predicting mutational effects on protein stability. Amino acids are grouped into three size classes based on van der Waals volume (as listed), and predictions are stratified by wild-type and mutant size classes; “similar sizes” denote substitutions within the same class. Stabilizing-versus-destabilizing classification metrics (rows) are then computed and shown for each stratum (columns), using ΔΔG<0 (experimental) and Δlogp>0 (predicted) as cutoffs for stabilizing mutations. P-values for pairwise model comparisons are shown in Fig. S3, and p-values comparing each model’s performance on small→large vs. large→small substitutions are shown in Fig. S4. Model names indicate the architecture, the coordinate-noise amplitude used during pre-training as standard deviation in Å units, and when applicable, the fine-tuning dataset (listed after “+”).

To test whether packing awareness preferentially improves predictions for substitutions that perturb local packing, we stratified mutations by residue size. Wild-type and mutant residues were assigned to small, medium, or large classes using normalized van der Waals volumes [39], and we evaluated stabilizing-versus-destabilizing classification performance within each wt → mt size-transition group (Fig. 3).

Switching from HERMES-*fixed* to HERMES-*relaxed* yielded the largest recall gains in identifying stabilizing substitutions between residues with substantially different sizes. This improvement was most pronounced for large→small substitutions: HERMES-*fixed* is constrained by the rigid wild-type cavity, preventing it from recognizing that mutations that create voids could be stabilizing, whereas HERMES-*relaxed* can repack neighbors to fill the space and stabilize the smaller side chain. While precision changes varied across size categories, F1-score improved in all categories, with the largest gains occurring for small→large substitutions, where relaxation can reorganize the local environment to accommodate bulkier side chains that would otherwise clash. Interestingly, HERMES-*amortized*, which learned packing implicitly through fine-tuning, showed comparable improvements for large→small substitutions but much weaker gains for small→large substitutions. This could suggest that stabilizing small→large substitutions may be underrepresented in the fine-tuning training set; the p-values associated with the significance of performance differences between models within each size-transition category, and within models across different size-transition categories are reported in Figs. S4, S5.

For comparison, we evaluated ProteinMPNN [35] on the same tasks. ProteinMPNN outperformed HERMES-*fixed*, and performed on par with HERMES-*relaxed* on both small→large and large→small mutations (Fig. 3, S3). We attribute this performance to ProteinMPNN’s input representation: by conditioning only on backbone coordinates and residue identities, ProteinMPNN is less constrained by the explicit wild-type side-chain geometry compared to the all-atom HERMES-*fixed* model. This architectural choice enables implicit reasoning about side-chain flexibility, achieving results comparable to using explicit relaxations in HERMES-*relaxed*. Notably, ProteinMPNN performed better than HERMES-*amortized* on small→large substitutions.

#### Fine-tuned HERMES models achieve state-of-the-art performance for stability effect prediction.

When fine-tuning on the respective stability effect datasets, HERMES outperformed RaSP (Fig. S6), and matched the performance of Stability-Oracle (Fig. 2A, S7) and ThermoMPNN (Fig. 2B). These results underscore both the effectiveness of the HERMES architecture and the importance of fine-tuning data for test-time accuracy. HERMES was also robust to using ESMFold-resolved structures [40] for either fine-tuning or inference (Fig. S8), supporting practical use cases where only computationally predicted structures are available.

Removing pre-training on wild-type amino-acid classification significantly degraded performance: HERMES models trained *only* for stability prediction performed poorly when trained on either cDNA117k or Megascale, even after reducing capacity from 3.5M parameters to 50k to mitigate overfitting (Fig. S2). A similar failure mode was reported for ThermoMPNN on the Megascale training data [30].

Finally, fine-tuning on stability effects largely eliminated size-dependent bias, yielding comparable recall and F1 scores for small→large and large→small substitutions (Figs. 3, S4).

#### Biophysical interpretation of HERMES predictions for mutational stability effects.

We sought to quantify how much models’ mutation preferences could be explained by amino-acid physicochemical properties. Specifically, we asked which properties are shared by amino-acid pairs that the model treats as interchangeable, i.e., substitutions with $\text{Δ}\hat{F}\approx 0$ on average.

To do this, we constructed model-averaged substitution matrices $M^{\text{model}}$ whose (α,β) entry represents how neutrally the model treats exchanging amino acids α and β. For each pair (α,β), we computed the model-predicted mean *absolute* effect, $M_{\alpha ,\beta }^{\text{model}}=\langle |\text{Δ}{\hat{F}}_{\alpha ,\beta }^{\text{(model)}}|\rangle$, where 〈⋅〉 denotes averages over all α→β and β→α substitutions in the Megascale test set. This procedure yields a symmetric, non-negative 20×20 matrix for each model, in which values approaching zero indicate greater predicted interchangeability. For comparison, we constructed an analogous matrix using experimentally measured |ΔΔG| values from the Megascale test set $\left(M^{\left|\text{ΔΔ}G\right|}\right)$, and include the BLOSUM62 substitution matrix as an additional reference.

Following ref. [41], we also constructed symmetric, nonnegative matrices of *absolute amino-acid property differences*. Specifically, we considered 50 quantitative properties spanning (i) hydrophobic, (ii) electronic, and (iii) steric categories (Table S3), yielding 50 property-specific matrices $M^{\text{prop}}$ with the (α,β) entry: $M_{\alpha ,\beta }^{\text{prop}}=\left|{\mathrm{prop}}_{\alpha }-{\mathrm{prop}}_{\beta }\right|$. Smaller values indicate greater similarity between the two amino acids with respect to the specified property.

**Figure 4:**
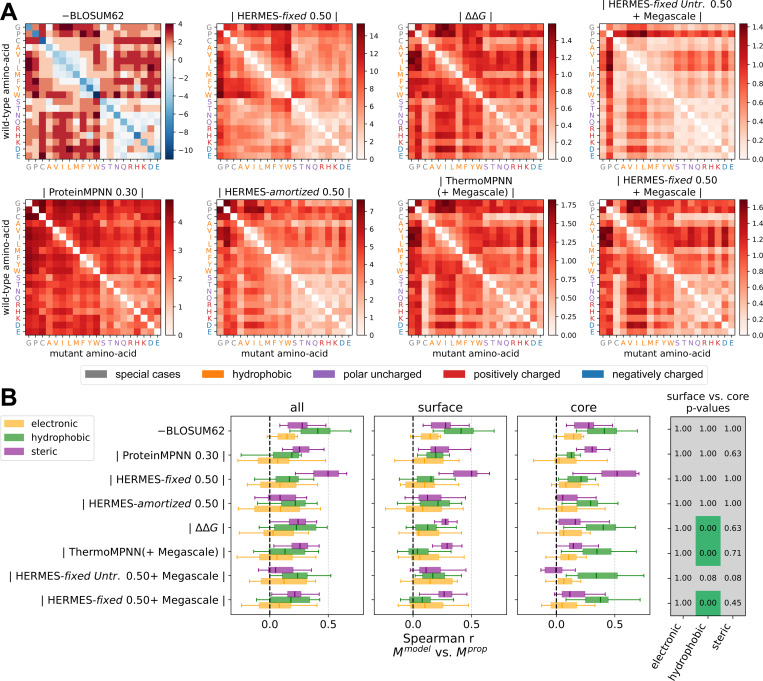
Uncovering mutation preferences via model-averaged substitution matrices. **(A)** Heatmaps of model-averaged substitution matrices $M^{\text{model}}$ computed by averaging over the Megascale test set, shown alongside BLOSUM62 and and the experimental matrix of mean |ΔΔG| values across mutations. Spearman correlations between matrices are reported in Fig. S10. Core- and surface-restricted matrices (core: SASA < 1*Å*^2^; surface: SASA > 3*Å*^2^) are shown in Fig. S11 and S12. **(B)** For each model and site subset (all, surface, core), boxplots summarize Spearman correlations between $M^{\text{model}}$ and property-difference matrices $M^{\text{prop}}$, grouped by property class (color). P-values from two-tailed t-tests comparing the surface vs. core correlations within each property class are shown on the right. P-values for between-model comparisons of property-class correlations are shown in Fig. S9.

To quantify which biophysical properties each model tends to preserve, we computed the Spearman correlation between the model-averaged substitution matrix $M^{\text{model}}$ and each of property-specific amino acid distance matrix $M^{\text{prop}}$ (Fig. 4B). Consistent with the size-stratified analysis (Fig. 3), HERMES-*fixed* predicted amino-acid interchangeability align most strongly with steric properties, particularly average buried-residue volume (Spearman r=0.66) and normalized van der Waals volume (Spearman r=0.64). This correlations was significantly stronger than for the other models (p-values in Fig. S9). In contrast, BLOSUM62, derived from evolutionary substitution frequencies, preferentially preserves hydrophobic properties. When restricting $M^{\text{model}}$ to core residues (SASA < 1 Å^2^), the interchangeability matrix associated with stability-fine tuned models (e.g. $M^{\text{HERMES}-fixed0.50\;+\;\text{Megascale}}$) showed stronger alignment with hydrophobic properties, to levels comparable to BLOSUM62 and consistent with the experimental matrix $M^{\left|\text{ΔΔ}G\right|}$. Zero-shot models instead did not show a comparable increase in hydrophobic preservation when restricting to the core (Fig. 4B).

#### Reversibility and path-independence of HERMES predictions with respect to mutations.

Mutational effects are, in principle, reversible: the effect of substituting a residue from amino acid α to β should be equal in magnitude and opposite in sign to the reverse change, i.e., $\text{Δ}F_{\alpha \to \beta }=-\text{Δ}F_{\beta \to \alpha }$. Moreover, equilibrium quantities such as protein stability free energy are state functions. As such, the net effect of a multi-step substitution depends only on the initial and final amino acids, not on the mutational path. For a path α→β→γ, this implies: $\text{Δ}F_{\alpha \to \gamma }=\text{Δ}F_{\alpha \to \beta }+\text{Δ}F_{\beta \to \gamma }$.

HERMES predicts mutation effects as differences in amino-acid–specific log-probabilities (logits) $\log p\left(aa|X_{aa}\right)$ (both zero-shot and after fine-tuning). As log-probability differences under a fixed structural context, these predictions satisfy reversibility by construction:

(4) $$\text{Δ}{\hat{F}}_{\alpha \to \beta }^{\left(\text{model}\right)}=\log P\left(\beta |X_{\beta }\right)-\log P\left(\alpha |X_{\alpha }\right)=-\text{Δ}{\hat{F}}_{\beta \to \alpha }^{\left(\text{model}\right)}$$

where we set $X_{\alpha }=X_{\beta }$ for HERMES-*fixed* and HERMES-*amortized*, and $X_{\beta }={\hat{X}}_{\alpha \to \beta }$ for HERMES-*relaxed*. Path-independence follows similarly. In contrast, other structural models such as Stability-Oracle [27] require explicit 19× data augmentation (termed “thermodynamic permutation augmentation” in ref. [27]) to enforce these properties.

Alternatively, reversibility can be assessed in a structure-conditioned manner [2, 30], where the effects of forward α→β and reverse β→α substitutions are computed using the outgoing structural contexts, i.e., α→β is conditioned on $X_{\alpha }$ and β→α on $X_{\beta }$ (Methods). Under this transformation, we do not automatically expect a “structure-conditioned reversibility”, as forward and reverse transitions are conditioned on different structures. However, a well-balanced model should have near-reversibility in this setting, making this a stringent test of model bias. We measured structure-conditioned reversibility on the Ssym dataset, which contains wild-type and single-mutant structures for 352 mutations across 19 proteins [42]. For each mutation, we computed a “forward” effect (wt → mt, conditioned on the wild-type structure) and a “reverse” effect (mt → wt, conditioned on the mutant structure).

**Figure 5:**
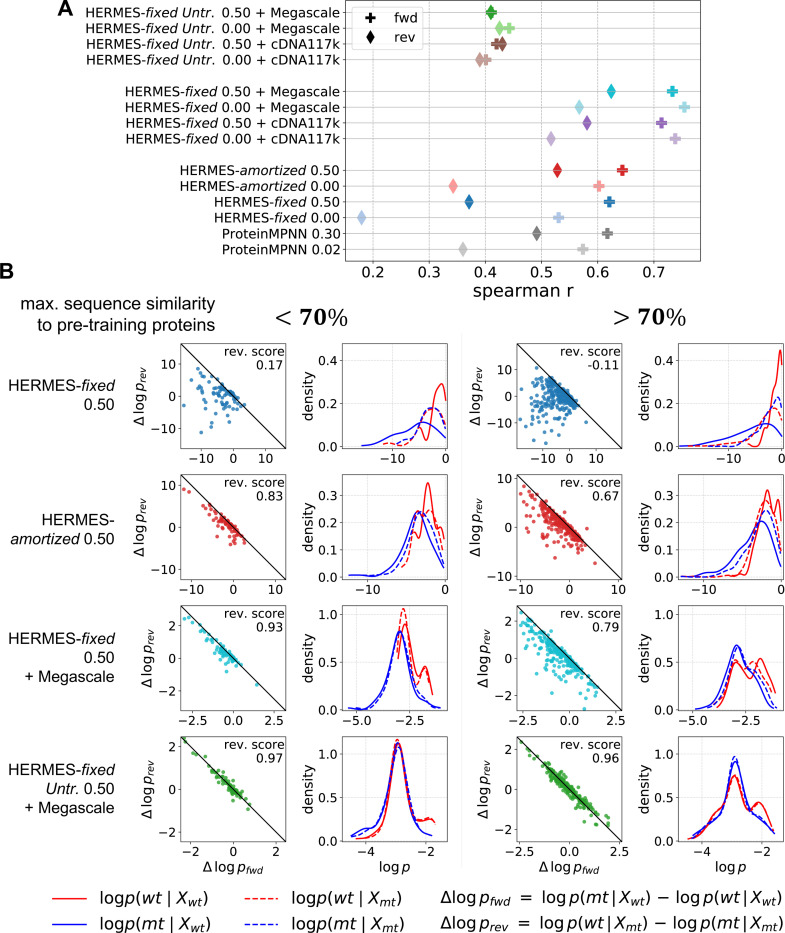
Identifying model biases with structure-conditioned reversibility **(A)** Spearman correlations between experimental stability changes (ΔΔG and model predictions on the Ssym dataset are shown. For each model, we report correlations for forward substitutions ($\text{Δ}\log p_{fwd}$ vs. ΔΔG) and the reverse substitutions ($\text{Δ}\log p_{rev}$ vs. −ΔΔG). **(B)** Ssym proteins are stratified by their maximum sequence identity to pre-training proteins (≥ 70% vs. < 70%). For each split (columns) and four representative HERMES variants (rows), the left panel shows the scatter plots for $\text{Δ}\log p_{fwd}$ vs. $\text{Δ}\log p_{rev}$; an unbiased model should exhibit strong anti-correlation, summarized by the reversibility score (rev.; higher is more reversible; Eq. 8). Reversibility is highest for the model not pre-trained on wild-type amino-acid classification (green; bottom row) and is consistently higher for the low-similarity subset. Fig. S13 shows the reversibility scores across all models in (A). In each column, the right panel shows the distributions of the log-probabilities that make up $\text{Δ}\log p_{fwd}=\log p\left(\text{mt}|X_{\text{wt}}\right)-\log p\left(\text{wt}|X_{\text{wt}}\right)$ (solid lines) and $\text{Δ}\log p_{\text{rev}}=\log p\left(\text{wt}|X_{\text{mt}}\right)-\log p\left(\text{mt}|X_{\text{wt}}\right)$ (dashed lines). All models except for the one that was *not* pre-trained on wild-type amino-acid classification (last row) exhibit elevated $\log p\left(\text{wt}|X_{\text{wt}}\right)$.

We found that zero-shot HERMES models and ProteinMPNN predict stability effects more accurately for forward substitutions (wt → mt) than for the reverse direction, consistent with previous observations [2, 30] (Fig. 5A). Adding coordinate noise during pre-training and fine-tuning on stability effects both reduced this forward–reverse disparity, but did not eliminate it. Models trained *only* on stability effects showed little or no disparity, albeit with substantially lower overall accuracy. Across all pre-trained models, the predicted stability effects of forward mutations from the wild-type tend to have larger magnitudes than those of the corresponding reverse mutations (Fig. 5B, scatterplots). This bias stems from the elevated log-probabilities assigned to wild-type amino acids in wild-type structure neighborhoods $\log p\left(\text{wt}|X_{\text{wt}}\right)$ (Fig. 5B, density plots), suggesting a wild-type preference that may reflect pre-training memorization. Consistently, stratifying Ssym proteins by sequence similarity to the pre-training set (Methods) yielded a modest reduction in wild-type preference for lower-similarity proteins (Fig. 5B). A more detailed characterization of this bias is left to future work.

### Antigen stabilization with HERMES for vaccine design

Structure-based vaccine design seeks to increase vaccine efficacy by stabilizing viral envelope glycoproteins (hereafter, “antigens”) in their metastable pre-fusion conformation. Stabilization enriches presentation of neutralization-relevant epitopes and can bias elicited immune responses toward protective specificities [9–13].

To test whether HERMES can identify antigen-stabilizing mutations, we benchmarked model performances on 33 previously reported antigen-stabilizing mutations drawn from five viral antigens: Influenza HA [12] (3 mutations), RSV-F [9, 43] (7 mutations), hMPV-F [10, 11, 13] (11 mutations), DENV-E [13] (8 mutations), and SARS-CoV-2 spike protein [44] (4 mutations). We scored mutations using zero-shot and stability-fine-tuned variants of ProteinMPNN and HERMES. Importantly, none of the stabilized variants in this benchmark appeared in the training data of any HERMES model, either during pre-training or during stability fine-tuning.

**Figure 6:**
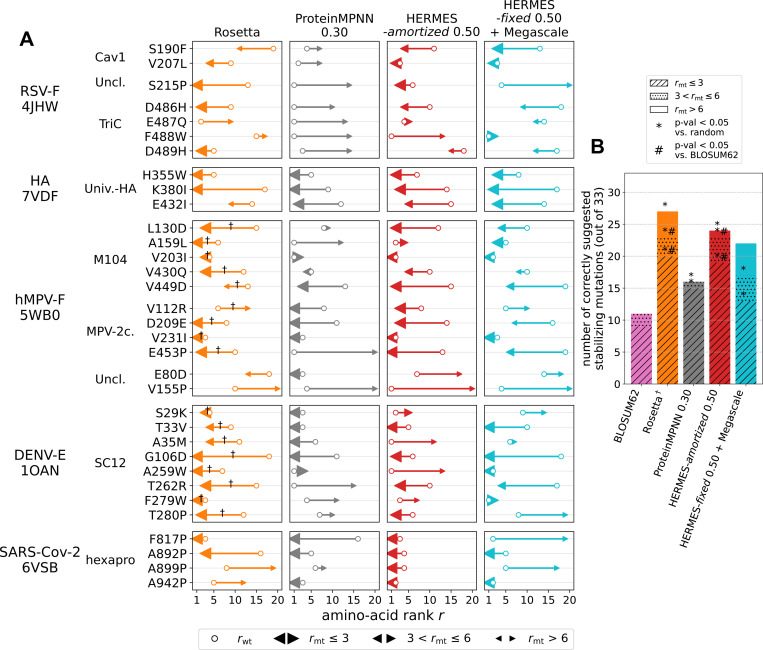
Predicting antigen-stabilizing mutations with HERMES. **(A)** Model recall is evaluated on 33 previously reported antigen-stabilizing mutations (rows) across five viral antigens. For each antigen, the PDB structure used for scoring is indicated. Mutations are specified as wild-type→mutant substitutions at the annotated site. Four models (columns) are compared, as labeled above each column; see Table S4 for a more extensive comparison of models on this task. Arrows depict the change in predicted rank from the wild type (open circle) to the stabilizing mutant (arrow tip); arrow size indicates whether the mutant ranks in the top 3 (large), ranks 4–6 (medium), or ranks > 6 (small). The † symbols mark mutations originally proposed as stabilizing by Rosetta. “Univ.-HA” stands for “Universal-HA”; “MPV-2c.” stands for MPV-2cREKR; “Uncl.” stands for “Uncleaved Prefusion-Closed”. **(B)** Counts of correctly prioritized antigen-stabilizing mutations $\left(r_{\text{mt}}<r_{\text{wt}}\right)$, stratified by predicted rank group: strongly suggested $\left(r_{\text{mt}}\leq 3\right)$, moderately suggested $\left(3<r_{\text{mt}}\leq 6\right)$, or weakly suggested $\left(6\leq r_{\text{mt}}\right)$ are shown for each model. For comparison, BLOSUM62 is used to rank substitutions into the $\left(r_{\text{mt}}\leq 3\right)$ or $\left(3<r_{\text{mt}}\leq 6\right)$ groups; under this scheme the wild-type residue is always ranked first. Statistical enrichment is assessed by comparing the number of strong/moderate recalls to a random-ranking baseline and to BLOSUM62 (denoted by (*) and (#), respectively, when binomial tests corrected p-value< 0.05; see Methods). We note that all structures were considered in their native multimeric state, generating symmetric partners with PyMOL when necessary.

To quantify performance, we emulated a simple model-guided selection workflow in which a practitioner considers substitutions at a site in descending order of model scores. For each known stabilizing mutation, we record its rank $r_{\text{mt}}$ among all 20 amino acids at that position and compare it to the wild-type rank $r_{\text{wt}}$. We posit that a practitioner would select a particular mutant for experimental validation if (1) its rank $r_{\text{mt}}$ is better (lower) than that of the wild-type $r_{\text{wt}}$, and (2) its rank is among the top-scoring candidates (lower end). We categorize each predicted-stabilizing mutation (i.e., with $r_{\text{mt}}<r_{\text{wt}}$) as strongly suggested $\left(r_{\text{mt}}\leq 3\right)$, moderately suggested $\left(3<r_{\text{mt}}\leq 6\right)$, or weakly suggested $\left(r_{\text{mt}}>6\right)$ (Fig. 6 and Table S4). This scheme would group mutations with similar physico-chemical properties into the same rank class. Consequently, models are generally not penalized for ranking a biophysically similar alternative above a known stabilizing mutant, making our performance metrics robust to fine-grained rank differences in different models (see Fig. S14 and SI for a more detail discussion on antigen-stabilizing mutations).

**Figure 7:**
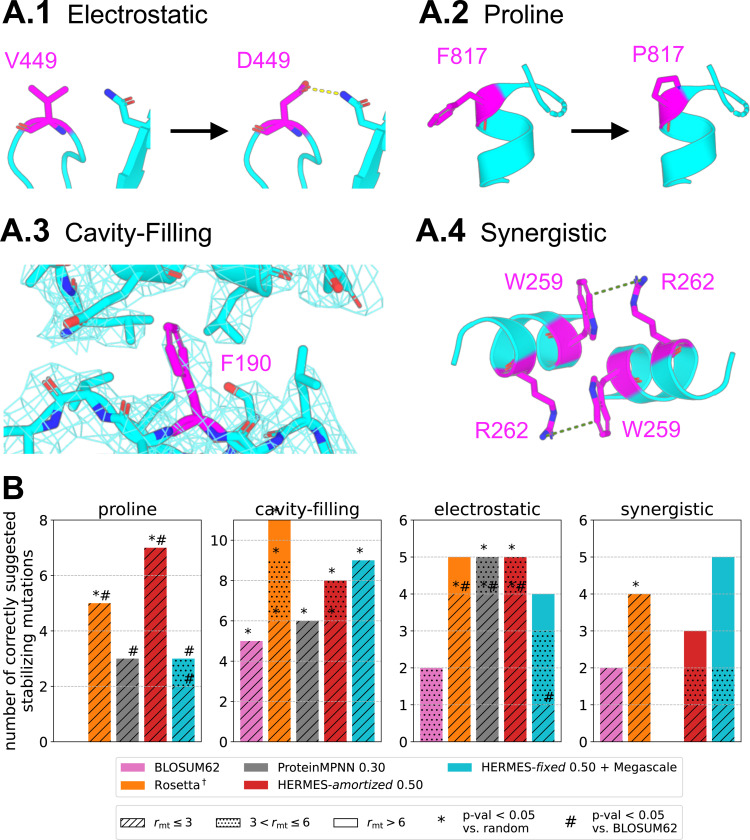
Model ability to recover different mutation patterns that stabilize antigens. **(A)** Representative examples of mutation types commonly seen in antigen stabilization [45], and analyzed in this study. The mutated residue(s) are shown in magenta. **(A.1)** *Electrostatic mutation* in hMPV-F: wild-type structure (PDB ID 5WB0, left), in-silico mutant generated with PyMOL’s mutagenesis wizard; right. The introduced Aspartic acid forms an electrostatic interaction with a nearby Asparagine (dashed yellow line). **(A.2)** *Proline mutation* at the N-terminal of a *α*-helix cap in SARS-CoV-2 spike: wild-type structure (PDB ID 6VSB; left), in-silico mutant generated with PyMOL’s mutagenesis wizard; right. **(A.3)** *Cavity-filling mutation* in RSV-F: mutant structure (PDB ID 4MMS). A bulky hydrophobic substitution packs a previously underfilled region: the 2*Fo*-*Fc* electron density map is shown as a thin mesh. **(A.4)** *Synergistic dimer-stabilizing mutations* in DENV-E: A259W and T262R in the dimer mutant structure (PDB ID 6WY1). introduced in both chains, create a stabilizing cation-*π* interaction (dashed green lines). **(B)** Number of stabilizing mutations recovered by each model, stratified by mutation class, reported as in Fig. 6B.

Figs. 6, 7 and Table S4 summarize predictions across the 33 stabilizing mutations we studied. Among the machine-learning methods, HERMES-*amortized* performs best: it assigns a better (lower) rank than wild-type to 24 stabilizing mutations, including 19 classified as strongly suggested $\left(r_{\text{mt}}\leq 3\right)$. Notably, stability fine-tuning does not consistently improve performance over the corresponding zero-shot ProteinMPNN and HERMES-*amortized* on this task (Table S4).

For comparison, we also scored each mutant with Rosetta [34] (Methods). Rosetta recovers stabilizing mutations on par with HERMES-*amortized* (Fig. 6 and Table S4); however, 17 variants in this benchmark were originally selected (by the references that discovered the variants) using Rosetta-based screening, which partially biases this baseline in Rosetta’s favor. Moreover, Rosetta inference is substantially more computationally intensive than the machine learning models, limiting its practicality for predicting the stability effects in high-throughput saturation mutagenesis (Table S5).

To assess statistical enrichment of model predictions over chance, we compared the predicted number of stabilizing mutations in the strong/moderate categories to a random-ranking baseline using a binomial test (Methods); p-values are reported in Fig. S15. All models except for HERMES-*fixed* and ThermoMPNN significantly outperform random ranking in recovering stabilizing mutations with strong or moderate confidence (Table S4). As an additional baseline, we tested whether the BLOSUM62 (B62) substitution matrix can prioritize stabilizing mutations. BLOSUM62 recovers significantly fewer stabilizing mutations in the strong/moderate categories than HERMES-*amortized* (defined as $r_{\text{mt}}^{B62}\leq 3$ and $r_{\text{mt}}^{B62}\leq 6$, noting that $r_{\text{wt}}^{B62}=1$ always; results shown in Fig. 6, and binomial test p-values reported in Fig. S15), underscoring the value of incorporating structural context when predicting stabilizing substitutions.

Practitioners commonly classify mutations in different types based upon the mechanism by which they stabilize pre-fusion antigens [45]. We consider four types, examples of which are shown in Fig. 7A. (i) *Electrostatic mutations*, which introduce polar or charged amino acids in environments where they can engage in stabilizing electrostatic interactions (Fig. 7A.1); (ii) *Proline mutations*, which are often used to impede post-fusion helix formation and stabilize pre-fusion conformations, due to proline’s unique lack of amide hydrogens which prevents it from taking part in stable *α*-helices, except at the N-terminal end of a helix cap (Fig. 7A.2); (iii) *Cavity-filling mutations*, which stabilize a particular conformation by filling cavities located within the hydrophobic core of an antigen by inserting a larger hydrophobic side-chain (Fig. 7A.3); (iv) *Synergistic mutations*, which involve applying multiple mutations so that they positively interact through a variety of mechanisms (Fig. 7A.4).

Based on these categories, we manually annotated the 33 antigen-stabilizing mutations (Table S6), and stratified model performance by mutational types (Fig. 7B). Among the models tested, HERMES-*amortized* most reliably recovers stabilizing proline mutations (7 out of 8; Fig. 7B), highlighting its potential utility for proposing pre-fusion stabilizing prolines. More broadly, all models recover proline and electrostatic mutations at higher rates than expected under BLOSUM62, whereas cavity-filling mutations show weaker gains. A plausible explanation is that cavity-filling changes typically replace a smaller hydrophobic residue with a larger one that preserves core hydrophobicity. Because such substitutions are common in natural sequence variation, they are partly captured by a sequence-derived matrix like BLOSUM62. In contrast, proline and charged substitutions are strongly context-dependent, with effects that hinge on local geometry and environment, and therefore benefit more from structure-aware modeling; see SI for a more extensive discussion.

Lastly, the synergistic RSV-F TriC mutations are difficult to recover for almost all methods (Fig. 6). This is expected for the three “acid patch neutralization” substitutions D486H, D489H, and E487Q [9], which were experimentally stabilizing only in combination (i.e., synergy) with F488W [9]. These four residues are tightly clustered in both intra- and inter-chain space, consistent with a synergistic (epistatic) mechanism in which the substitutions act synergistically to stabilize the trimer. A second example of synergy is the DENV-E pair A259W and T262R, which together introduce a favorable cation–*π* interaction (Fig. 7A.4). Because our evaluation scheme scores mutations in a site-independent manner, the models are not able to capture such multi-residue dependencies. Specifically, HERMES scores mutations at a single site under the assumption that all other amino-acid identities remain fixed, and therefore, cannot recover stabilization mechanisms that arise only from specific combinations of mutations. ProteinMPNN, ThermoMPNN, and Rosetta are likewise evaluated here under the same site-independent protocol for a fair comparison, although they can in principle be applied to score multi-residue variants jointly; see SI for a more extensive discussion.

Beyond the inability to capture epistatic effects, several additional limitations of our approach should be noted. First, to emulate a practitioner’s workflow we evaluate mutations using only their relative ranks; however, the absolute magnitudes of model scores carry additional information and could enable more robust decision-making when prioritizing antigen-stabilizing mutations. Second, because the original studies rarely tested comprehensive alternative substitutions at the same sites, we cannot evaluate the models’ ability to propose different stabilizing mutations that were not assayed experimentally. Third, experimental validation frequently involved multi-mutation constructs, complicating attribution of observed stabilization to any single residue. Finally, the modest number of curated stabilizing mutations limits statistical power and the strength of conclusions in our analyses. Despite these caveats, our results suggest that HERMES can serve as a practical screening tool for rational library design, prioritizing candidate antigen-stabilizing mutations for more efficient experimental validations.

### Predicting binding effect of mutations

Predicting the effects of mutations on binding affinity is a central step in target-specific protein design. In prior work, we demonstrated the utility of HERMES for predicting how mutations in short peptide antigens alter binding to T-cell receptors in the context of MHC complexes [53]. We further leveraged this capability for de novo design of peptide antigens intended to elicit specific T-cell responses [53]. Here, we evaluate HERMES on the single-point mutation effects from the SKEMPI v2.0 dataset [51], which spans a substantially broader range of protein–protein interactions and binding-affinity perturbations.

In a zero-shot setting, HERMES exhibits measurable predictive signal (HERMES-*fixed* 0.50; Spearman’s ρ=0.286). ProteinMPNN achieves comparable overall performance, whereas physics-based approaches (Rosetta [34] and FoldX [22]) perform modestly better (Spearman’s ρ≈0.35). In contrast to our results for stability prediction, HERMES-*fixed* models correlate more strongly with binding effects than HERMES-*amortized* models. Interestingly, fine-tuning for stability (HERMES-*fixed* 0.00+ cDNA117k) yields a slight improvement in binding-effect prediction. A detailed comparison across models is provided in Table 1.

Next, we fine-tuned HERMES directly on SKEMPI using 3-fold cross-validation. To control for information leakage under structural similarity, we introduced three homology-aware splitting strategies of increasing difficulty; the most stringent split prevents complexes from the same interaction “class” (e.g., antibody–antigen or TCR–pMHC) from appearing in different folds; see Methods for details. Fine-tuned HERMES models achieve substantially stronger correlations, including under the *Difficult* split (Table 1).

Finally, we compared HERMES to recent state-of-the-art methods, RDE-Network and MIF-Network [33], DDGPred [48], ESM-IF [49] and Pythia-PPI [50]. These baselines were also trained on SKEMPI using 3- or 5-fold cross-validation under protocols analogous, but not identical, to our *easy* split. HERMES fine-tuned on SKEMPI-Easy performs competitively with RDE-Network and MIF-Network, but trails Pythia-PPI (Table 1). Notably, RDE-Network and MIF-Network are fine-tuned on SKEMPI ΔΔG labels in a manner similar to our approach, whereas Pythia-PPI incorporates additional training procedures that further boost performance (Methods). These procedures are, in principle, applicable to HERMES as well, but we leave a systematic evaluation of such extensions, and of HERMES as a general framework for binding-affinity prediction, to future work.

## Discussion

We introduced HERMES, a family of fast, structure-based machine learning models for predicting the effects of mutations on protein function. HERMES leverages SO(3)-equivariant neural networks operating on local atomic neighborhoods in a protein structure to predict amino acid propensities, whose differences can be used to predict mutational effects. This formulation yields a computationally efficient predictor that is naturally suited to high-throughput settings. HERMES captures mutational impacts on diverse phenotypes, including thermodynamic stability and protein–protein binding affinity, and it provides a practical tool for antigen stabilization in vaccine design.

A central finding is that encoding packing flexibility is essential for accurate predictions across mutations involving amino acids of different sizes. HERMES-*fixed*, a lightweight protocol that evaluates mutations in the rigid wild-type structure, exhibits strong bias toward size-conserving substitutions. Explicitly modeling relaxation via Rosetta [34], (HERMES-*relaxed* protocol) resolves this bias but incurs substantial computational cost. Our amortized approach offers an effective compromise: by fine-tuning on relaxed predictions for just 0.5% of pre-training data, HERMES-*amortized* learns implicit packing flexibility that can be leveraged for predictions at HERMES-*fixed* speed. This capability proves particularly valuable for antigen stabilization, where HERMES-*amortized* recovers over half of the verified stabilizing mutations in our benchmark and shows strong performance on proline substitutions (87.5% recovery), which stabilize proteins by restricting backbone conformational freedom.

Beyond zero-shot use, HERMES can be fine-tuned directly on experimental labels using a simple end-to-end procedure. Notably, both the native and the fine-tuned models use the difference of amino acid scores for predicting mutational effects, which yields an inherent reversibility with respect to mutation order–a thermodynamic consistency property that is often enforced via explicit 19× data augmentation [27].

With fine-tuning, HERMES achieves competitive accuracy on thermodynamic stability benchmarks when trained on the same data as prior methods [2, 27, 30]. Interestingly, stability fine-tuning does not consistently transfer to antigen stabilization and can even degrade performance. One plausible explanation is dataset mismatch: the high-throughput stability datasets used for fine-tuning [20] are enriched for relatively small, compact domains, whereas vaccine antigens are often larger, multi-domain, and conformationally heterogeneous, with stabilizing mutations frequently targeting quaternary contacts, glycoprotein-specific features, or prefusion-state constraints. Resolving this discrepancy will likely require broader supervision that better reflects antigen structure and design objectives, or task-aligned fine-tuning data.

We also demonstrate that pre-training on wild-type amino acid classification remains necessary for strong performance; models trained only on currently-available experimental stability data fail. Notably, pre-trained models exhibit “wild-type preference,” assigning elevated probabilities to wild-type residues in wild-type structural contexts. This bias correlates with sequence similarity to pre-training proteins, suggesting partial memorization rather than purely generalizable learning. Fine-tuning partially ameliorates but does not eliminate this effect, representing a fundamental trade-off in current training paradigms. More robust pre-training objectives, stronger regularization, or explicit debiasing strategies may be required to fully address this issue.

A key application we highlight is structure-based vaccine design, where practitioners seek to stabilize substitutions that preserve a desired prefusion conformation of a vaccine antigen to elicit immune responses targeting relevant epitopes. For this task, a useful model should propose stabilizing mutation candidates across different sites of an antigen. On the limited available data comprising 33 verified antigen-stabilizing mutations across five viruses, HERMES performs well in recovering these mutations. Moreover, the strict locality of the model makes it straightforward and fast to apply to large antigens and assemblies that can pose challenges for architectures modeling global interactions in proteins. Our results show that local all-atom environments modeled by HERMES can encode multiple stabilization mechanisms, including cavity filling, backbone rigidification via proline, and favorable electrostatic remodeling, while also underscoring limitations for mechanisms that depend on long-range coupling, or multi-site epistasis.

We further evaluated HERMES on predicting changes in protein–protein binding affinity using the SKEMPI dataset [51]. In the zero-shot setting, HERMES shows measurable predictive signal, and supervised fine-tuning on SKEMPI substantially improves performance. Recent state-of-the-art approaches for binding prediction like Pythia-PPI [50] indicate that design choices such as self-distillation can substantially improve performance [50]; we expect HERMES would similarly benefit from such approaches, representing a clear next step.

Several directions could extend HERMES’ capabilities. Developing larger, more diverse training datasets, particularly for antigen stabilization and binding, would likely improve performance, as our results indicate that training data dominates architectural differences in determining benchmark performance. The locality of HERMES appears to be a double-edged sword: local neighborhoods reduce input complexity, remove constraints on protein size, and improve scalability, but necessarily limit the model’s ability to capture long-range epistasis and allosteric coupling. We view HERMES as a hypothesis-generation tool whose predictions are most reliable when mutational effects are driven primarily by short-range interactions and local packing. Productive directions for future work include better characterizing when locality suffices, further reducing pre-training-induced biases, and developing multi-scale approaches that efficiently combine local all-atom predictors with complementary global or multi-site models to capture mechanisms beyond the local neighborhood.

## Methods

### HERMES architecture

The HERMES architecture closely follows our recent developments of SO(3)-equivariant neural network models for protein structures [26, 36, 37]. For completeness, we summarize here the main methodological components and refer the reader to those works for additional details.

The input to HERMES is a point cloud of atoms in a protein structure, which we term a *neighborhood*. Each neighborhood is centered at the C-*α* atom of a focal residue and includes all atoms within a 10 Å radius of this center. Optionally, we add Gaussian noise with standard deviation 0.50 Å to the atomic coordinates to achieve further model robustness. The output of HERMES is a 20-dimensional representation of the neighborhood that is invariant to 3D rotations about its center (i.e., SO(3)-invariant). To obtain SO(3) invariance, HERMES is constructed from SO(3)-equivariant layers that progressively compute higher-level SO(3)-invariant features, which are then passed to a final multilayer perceptron (MLP) with a 20-dimensional output. Conceptually, symmetry awareness in HERMES is achieved through two main components: (i) a (holographic) encoding of the neighborhood into a basis suitable for SO(3)-equivariant operations, and (ii) processing of the input via a stack of SO(3)-equivariant neural network layers to learn an expressive and SO(3)-invariant representation of the neighborhood.

#### Holographic encoding of atomic protein structure neighborhoods.

We first represent the atomic point cloud of each structural neighborhood as a density function obtained by superposing (weighted) Dirac-*δ* functions, indicating the presence of atoms at a given position in space: $\rho (r)=\sum_{i\in \text{points}}\omega_{i}\delta \left.\left(r_{i}-\right.r\right)$; here, $\omega_{i}$ indicates the weight associated with point i at position $r_{i}$, and $r_{i}$ can be decomposed in its constituents spherical components $\left(r_{i},\theta_{i},\varphi_{i}\right)$. We then use 3D Zernike Fourier Transform (ZFT) [26] of the density function to encode the neighborhood into a convenient SO(3) equivariant basis,

(5) $${\hat{Z}}_{\ell m}^{n}=\sum_{i\in \text{points}}\omega_{i}R_{n}^{\ell }\left(r_{i}\right)Y_{\ell m}\left(\theta_{i},\varphi_{i}\right)$$

where $Y_{\ell m}(\theta ,\phi )$ is the spherical harmonics of integer degree ℓ≥0 and integer order m≤|ℓ|, and $R_{\ell }^{n}(r)$ is the radial Zernike polynomial in 3D with integer radial frequency n≥0 and degree ℓ. $R_{\ell }^{n}(r)$ is non-zero only when n−ℓ is even and ≥ 0. We keep coefficients of up to and including ℓ=5, and, for every ℓ, we keep the first 11 non-zero radial frequencies.

Notably, the spherical harmonics that describe the angular component of ZFT arise from the irreducible representations of the 3D rotation group SO(3), and form a convenient basis under rotation in 3D (see the Appendix of [37] for a formal mathematical introduction). Zernike projections in spherical Fourier space can be understood as a superposition of spherical holograms of an input point cloud, and thus, we term this operation an *holographic encoding* of the data [26, 36]. The resulting holograms are primarily indexed by the degree ℓ: different values of ℓ encode components that transform in specific ways under 3D rotations. For example, the ℓ=0 component is rotation-invariant.

Following [26, 36], we incorporate atom-level features by partitioning the holographic encoding into multiple *channels* (Fig. 1A). Specifically, we use separate channels for C, N, O, S, computationally added hydrogens, partial charge, and solvent-accessible surface area (SASA). Partial charge and SASA are defined for all atoms, and their values are incorporated through the weights $\omega_{i}$.

#### SO(3)-equivariant neural network architecture.

The neighborhood holograms are then processed by a stack of SO(3)-equivariant layers (Fig. 1A). The final SO(3)-invariant representation is obtained by reading out the ℓ=0 component of the last layer and passing it through an MLP to produce a 20-dimensional embedding. We employ three types of SO(3)-equivariant building blocks, inspired by the H-CNN architecture [26] and also detailed in ref. [37]: (i) SO(3)-equivariant linear layers (Lin), which mix channels while preserving the SO(3) transformation properties; (ii) tensor-product nonlinearities (TP), which couple different irreducible components; and (iii) SO(3)-equivariant layer normalization (LN).

All SO(3)-equivariant components are implemented using e3nn primitives [54]. Within the same framework, we re-implemented the H-CNN architecture described in [26] for comparison. HERMES achieves a forward pass that is approximately 2.75× faster than H-CNN, while using a similar number of parameters (∼ 3.5M).

### Pre-processing of protein structure data

To pre-process protein structure data, we developed two distinct pipelines based on either (i) PyRosetta [34] or (ii) Biopython [55] together with other open-source tools, with code adapted from [2]. The PyRosetta-based pipeline is considerably faster but requires a license, whereas the Biopython-based pipeline is fully open source. We train models using data generated by both pipelines, with the constraint that the Pre-processing pipeline used at inference must match that used during training. Performance differences between the two pipelines are minor (Fig. S6 and S16, and Table S1); unless otherwise stated, we report results obtained with the PyRosetta pipeline, which yields slightly better performance and has faster Pre-processing runtime.

For the PyRosetta workflow, we use PyRosetta functionalities for all Pre-processing steps: repairing PDB files by adding missing residues, adding hydrogen atoms, assigning partial atomic charges, computing solvent-accessible surface areas (SASA); we ignore non-canonical amino acids. For the open-source Pre-processing workflow (“Biopython” pipeline), we proceed as follows. First, we use OpenMM [56] to repair PDB files by adding missing residues and substituting non-canonical residues with their canonical counterparts. Hydrogen atoms are then added using the reduce program [57]. Partial atomic charges are assigned from the AMBER99sb force field [58], and SASA are computed using Biopython [55].

Both pre-processing pipelines retain atoms belonging to non-protein residues and ions, in contrast to the RaSP pre-processing procedure [2]. Notably, the PyRosetta-based pipeline does not replace non-canonical residues.

### HERMES pre-training

We pre-trained HERMES using an inverse folding objective, in which the model predicts the identity of a masked focal amino acid (i.e., the native residue in the structure) given its surrounding atomic neighborhood. This training task is analogous to that used for H-CNN [26]. We adopted the same data splits as in H-CNN: 10,957 structures for training, 2,730 for validation, and 212 for testing. These splits are derived from ProteinNet’s [38] 30% sequence-identity clustering of PDB structures available at the time of CASP12.

Model parameters were optimized for 10 epochs using the Adam optimizer [59] with a learning rate of 10^−3^. We selected the model checkpoint with the lowest validation loss at the end of each epoch. A single HERMES model is implemented as an ensemble of 10 independently trained neural network instances, whose predictions are averaged at inference time. Pre-training a single network instance required approximately 40 minutes per epoch on a single NVIDIA A40 GPU.

### Rosetta relaxation of mutant structures for the HERMES-*relaxed* protocol

For the HERMES-*relaxed* protocol, we use PyRosetta [34] to generate and locally refine mutant protein structures. Specifically, the focal residue is first substituted with the desired mutant amino acid, after which all side-chain atoms within 12 Å of the focal residue’s C-*α* atom are relaxed using the FastRelax protocol with the ref2015_cart energy function and a single relaxation cycle.

We performed a targeted ablation study to select the relaxation parameters. These experiments indicate that allowing backbone atoms to relax slightly degrades performance, and that ensembling over multiple independent relaxation runs does not improve accuracy (Fig. S17A), while substantially increasing computational cost (Fig. S17B). Thus, all results for the HERMES-*relaxed* protocol are reported using side-chain–only relaxation with a single FastRelax cycle.

### Training HERMES-*amortized* to implicitly learn about relaxation

We fine-tuned HERMES to align HERMES-*fixed* predictions to those of HERMES-*relaxed*, on a subset of the pre-training protein sites. Specifically, we uniformly sampled 10% of the proteins pre-training training set (1,284), and from those we uniformly sampled 5% of the sites. We similarly sampled 1% of the sites from the proteins of the pre-training validation set, and 5% of the pre-training testing set.

### HERMES fine-tuning for downstream tasks

In all analyses, we fine-tuned HERMES models under a Huber Loss objective with hyperparameter δ=1.0, for 15 epochs using the Adam optimizer [59] with a learning rate of 10^−3^ and a batch size of 128 mutations, selecting the model checkpoint with the lowest validation loss at the end of each epoch. The only exception is the models without pre-training, which are trained for 25 epochs.

By convention, we define model outputs such that higher predicted values correspond to more favorable mutation effects. Accordingly, when fine-tuning on experimental ΔΔG measurements–where lower values indicate greater stability–we trained the model to predict −ΔΔG. This sign convention ensures consistent interpretation of model outputs across tasks and is fixed throughout all reported analyses.

To speed-up convergence during fine-tuning, we first rescale the weight matrix and bias vector of the network’s output layer so that the mean and variance of the output logits match those of the validation scores. This initialization step requires a forward pass through the validation data to estimate the mean and variance, but it makes the model outputs immediately on the same scale as the experimental scores. Thus, fine-tuning avoids spending early epochs merely adjusting output magnitudes.

Overall, fine-tuning is computationally efficient. Training a single neural network instance requires approximately 2.5 minutes per epoch on the cDNA117k dataset (∼117,000 mutations) and about 4 minutes per epoch on the Megascale training dataset (∼217,000 mutations) on a single NVIDIA A40 GPU.

### Fine-tuning datasets

#### Stability effect prediction.

For protein stability prediction, we fine-tuned and evaluated the performance of HERMES on the same datasets of three recent structure-based predictors of mutational stability effects–RaSP [2], Stability-Oracle [27], and ThermoMPNN [30]–so results are comparable. The data from these models are used in following way:

- *RaSP dataset.* We used the RaSP dataset [2] as provided by the authors on their github repository (https://github.com/KULL-Centre/2022ML-ddG-Blaabjerg). As in the original RaSP paper, the target values are Rosetta-computed stability changes $\left(\text{ΔΔ}G\right)$, which are known to be reliable primarily within the range [−7, 1] kcal/mol. To account for this, RaSP applies a sigmoid transformation to the raw ΔΔG values prior to training, effectively saturating the targets outside this interval.
  We adopt the same sigmoid transformation, with one modification: we center the transformed values such that ΔΔG=0 maps to zero after transformation. This centering is required by the HERMES output parameterization, in which the predicted stability change for a mutation to the same amino acid is constrained to be zero (i.e., $\text{ΔΔ}G_{aa_{i}\to aa_{i}}=0$). While this property holds for physical ΔΔG values, it is not preserved by the uncentered sigmoid transform used in RaSP. Our centered transformation therefore ensures consistency between the model’s output space and the physical interpretation of stability changes, and it follows,
(6) $$F(\text{ΔΔ}G)=\frac{1}{1+e^{-\beta (\text{ΔΔ}G-\alpha )}}-\frac{1}{1+e^{\beta \alpha }}$$
- *Stability-Oracle datasets.* Stability-Oracle introduces two curated datasets that we use in this work [27]:
  - cDNA117k (training set): derived from the Megascale cDNA display proteolysis dataset # 1 [20]. The original Megascale dataset reports approximately 850,000 thermodynamic folding stability measurements $\left(\text{Δ}G\right)$ across 354 natural and 188 de novo mini-protein domains (40–72 amino acids in length). Following the Stability-Oracle protocol, mutations are filtered to enforce at most 30% sequence identity with the test set, yielding a reduced set of approximately 117,000 mutation-induced stability changes $\left(\text{ΔΔ}G\right)$, referred to as cDNA117k.
  - T2837 (test set): assembled by combining several commonly used benchmarking datasets of experimentally measured ΔΔG values, including S669 [42], myoglobin [28], ssym [60], and p53 [61]. The resulting test set comprises 2,837 mutations.
    We obtained the cDNA117k and T2837 datasets from the Stability-Oracle [27] github repository (https://github.com/danny305/StabilityOracle/tree/master). At the time of access, the residue indices provided in the dataset did not correspond to the residue numbering in the original PDB files, but instead to an intermediate, post-processed representation that was not documented in sufficient detail to allow straightforward recovery of the original numbering. To ensure consistency with structural data, we manually corrected the residue numbers in the CSV files to match those in the corresponding PDB structures. The corrected versions of these datasets are included in our repository.
- *ThermoMPNN dataset.* ThermoMPNN also leveraged Megascale [20], but used datasets # 2 and # 3, curated and split by the authors at a 25% sequence-identity cutoff into 216k training and 28k test mutation effects [30]. We used the train, valid, and test splits of the Megascale dataset as curated in ThermoMPNN, and provided on their github repository (https://github.com/Kuhlman-Lab/ThermoMPNN). Throughout the text, we refer to these datasets as the Megascale training (train + valid) and testing sets. We downloaded the structures’ .pdb files from https://zenodo.org/records/7992926. We note that the Megascale training set was not controlled for maximum similarity with the T2837 dataset, and six of the T2837 proteins (∼ 5%) have sequence homologs in the Megascale training set with sequence similarity above 90%.

#### Binding effect prediction.

For predicting mutational effects on binding affinity, we fine-tuned HERMES on the SKEMPI v2.0 dataset [51], using 3-fold cross-validation.

- *SKEMPI dataset:* After removing duplicate experimental entries, SKEMPI v2.0 contains 5,713 measurements of binding free-energy changes $\text{(ΔΔ}G^{\text{binding}}\text{)}$ spanning 331 protein–protein complex structures. We further restrict the dataset to complexes with at least 10 annotated mutations, resulting in 116 structures and 5,025 mutations. Finally, we retain only single-point mutations, yielding 93 structures and 3,485 mutations.
  SKEMPI provides metadata explicitly designed to support leakage-aware evaluation via two fields: *hold-out type* and *hold-out proteins* (see SKEMPI documentation: https://life.bsc.es/pid/skempi2/info/faq_and_help). Briefly, *hold-out type* groups complexes into broad categories (e.g., protease-inhibitor, antibody-antigen, and pMHC-TCR), while *hold-out proteins* lists PDB identifiers and/or hold-out types that should be co-held-out to avoid training on closely related binding sites. Using this information, we define three split strategies:
  - *Easy:* random splitting without using hold-out metadata.
  - *Medium:* we enforce that all entries linked via a mutation’s *hold-out proteins* annotation are assigned to the same split (but we do not additionally group by *hold-out type*).
  - *Difficult:* we enforce that all complexes sharing the same *hold-out type* are assigned to the same split, producing a stringent evaluation of generalization across interaction classes.

In cases where a complex is associated with multiple hold-out types, we assign it to a single type by randomly selecting one of the available labels.

### Baseline models

Here, we describe the baseline models used to benchmark HERMES on mutational effect prediction.

#### ProteinMPNN [35].

ProteinMPNN is an inverse-folding model that samples amino-acid sequences conditioned on a protein backbone (optionally with a partial sequence fixed). Because it also outputs per-site amino-acid probabilities, we used it to score mutational effects via the log-likelihood ratio in Eq. 1. As for HERMES, we evaluate ProteinMPNN models trained with two noise levels: 0.02 Å(virtually no noise) and 0.30 Å. We used, and provide, scripts to infer mutation effects built upon a public fork of the ProteinMPNN repository (https://github.com/gvisani/ProteinMPNN-copy).

#### ThermoMPNN [30].

ThermoMPNN is a thermodynamic stability predictor built on top of Protein-MPNN. For a given structure, it extracts the final ProteinMPNN residue embedding at the site of interest and feeds it to a separate head that predicts per–amino-acid ΔG values, from which ΔΔG is computed. Similar to HERMES, this formulation enforces the permutation symmetry of mutational effects by construction, without requiring data augmentation. For our experiments, we used native functionalities in the ThermoMPNN repository (https://github.com/Kuhlman-Lab/ThermoMPNN).

#### Stability-Oracle [27].

Similar to HERMES, Stability-Oracle is trained in two stages. First, a graph-attention network is pre-trained to predict masked amino acids from their local atomic environment (“neighborhood”). Next, the embeddings from the pre-trained model is used to regress over mutation-effect. Specifically, For a target site on a structure, the masked-neighborhood embedding h is extracted from the pre-trained network and concatenated separately with embeddings of the “from” and “to” amino acids. Each concatenated input is passed through a transformer to produce amino-acid–specific embeddings $e_{aa_{\text{from}}}$ and $e_{aa_{\text{to}}}$, whose difference $\left(e_{aa_{\text{to}}}-e_{aa_{\text{from}}}\right)$ is fed to a two-layer MLP to predict $\text{ΔΔ}G_{aa_{\text{from}}\to aa_{\text{to}}}$. This construction is permutation-symmetric up to the final MLP, since each $e_{aa}$ is computed independently; symmetry is broken only by the MLP, and would have been preserved by a bias-free linear layer. The original method enforces symmetry during training via 19× data augmentation. We report performance scores calculated using predictions provided in the Stability-Oracale’s repository (https://github.com/danny305/StabilityOracle).

#### RaSP [2].

Similar to HERMES, RaSP is trained in two steps. First, a 3D CNN is pre-trained to predict masked amino acids from their local atomic environment (“neighborhood”). Then, a small fully-connected neural network with a single output is trained to regress over mutation effects, using as input neighborhoods’ embeddings from the 3DCNN, the one-hot encodings of wildtype and mutant amino-acids, and the wildtype and mutant amino-acids’ frequencies in the pre-training data. RaSP is fine-tuned on the stability effect of mutations ΔΔG, computationally determined with Rosetta [34]. We report performance scores calculated using predictions provided in the authors’ repository (https://github.com/KULL-Centre/_2022_ML-ddG-Blaabjerg).

#### RDE-Network [33].

RDE stands for Rotamer Density Estimator. This model consists of first a graph neural network encoder that is trained via a normalizing flow objective to predict distributions of side-chain conformations. Then, a prediction head is added, and it is trained on $\text{ΔΔ}G_{\text{binding}}$ effects from the SKEMPI dataset. We report performance scores as provided in ref. [33].

#### MIF-Network [33].

This model’s architecture is the same as the encoder in RDE-Network. It is first pre-trained on the task of wild-type amino acid classification. Then, a prediction head is added to the encoder, and it is trained on $\text{ΔΔ}G_{\text{binding}}$ effects from the SKEMPI dataset [51] in the same manner as RDE-Network. We report performance scores as provided in Ref. [33].

#### Pythia-PPI [50].

Pythia-PPI uses a graph neural network encoder pre-trained for wild-type amino acid classification, followed by a ΔΔG prediction module with two heads: one for $\text{ΔΔ}G_{\text{stability}}$ and one for $\text{ΔΔ}G_{\text{binding}}$. The model is fine-tuned jointly on stability labels from FireProtDB [52] and binding labels from SKEMPI [51], using a validation selected 20:80 stability:binding mixing ratio. The resulting checkpoint (“Vanilla Pythia-PPI”) is then further trained via self-distillation by fine-tuning on its own predictions over all SKEMPI complex structures to obtain the final model (Pythia-PPI). We should note that these two procedures (i.e., joint fine-tuning on stability and binding with a validation selected mixing ratio and self-distillation) could also be applied to HERMES to potentially improve performance. We report performance scores as provided in ref. [50].

### ESMFold for computational modeling of protein structures

For the analysis in Fig. S8, we used the ESM Metagenomic Atlas API to fold each sequence individually (https://esmatlas.com/resources?action=fold).

### Structure-conditioned reversibility analysis

In Figs. 5 and S13, we characterized structure-conditioned reversibility on the SSym dataset [60], which contains wild-type and single-mutant structures for 352 mutations across 19 proteins [42].

We assess structure-conditioned reversibility by whether the effects of forward α→β and reverse β→α mutations are equal, using the outgoing structural context to compute mutational effects. Specifically, the forward mutational effect $\text{Δ}{\hat{F}}_{\alpha \to \beta }^{\left(\text{model}\right)}$ is computed by conditioning on the structure $X_{\alpha }$, and the reverse effect $\text{Δ}{\hat{F}}_{\beta \to \alpha }^{\left(\text{model}\right)}$ is computed using $X_{\beta }$,

(7) $$\text{Δ}{\hat{F}}_{\alpha \to \beta }^{\left(\text{model}\right)}=\log P\left(\beta |X_{\alpha }\right)-\log P\left(\alpha |X_{\alpha }\right)\;\text{Δ}{\hat{F}}_{\beta \to \alpha }^{\left(\text{model}\right)}=\log P\left(\alpha |X_{\beta }\right)-\log P\left(\beta |X_{\beta }\right)$$

With this definition, we do not expect reversibility, as forward and reverse transitions are conditioned on different structures.

#### Reversibility score.

For the analysis in Figs. 5 and S13 we construct a reversibility score as a mean squared error normalized to be between −1 and 1,

(8) $$\mathrm{rev}.\mathrm{score}=1-\frac{\mathrm{mean}((\Delta \;\log\;p_{fwd+}\Delta \;\log\;p_{rev}{)}^{2})}{\mathrm{mean}\left(\Delta \;\log\;{p_{fwd}}^{2}\right)+\mathrm{mean}\left(\Delta \;\log\;{p_{rev}}^{2}\right)}$$

where $\text{Δ}\log p_{fwd}=\log p\left(\text{mt}|X_{\text{wt}}\right)-\log p\left(\text{wt}|X_{\text{wt}}\right)$ is the forward mutational effect computed by conditioning on the wild type structure, and $\text{Δ}\log p_{\text{rev}}=\log p\left(\text{wt}|X_{\text{mt}}\right)-\log p\left(\text{mt}|X_{\text{mt}}\right)$ is the reverse mutational effect computed by conditioning on the mutants structure. The averages (mean) are computed over the 352 mutations in the SSym dataset, or stratified as needed by the desired criteria. With this definition, a larger value of **rev. score** implies more degree of reversibility between forward and reverse mutations.

#### Sequence similarity calculation between the Ssym dataset and pre-training proteins.

We stratified the data based on their similarity to the training data. To do so, we used BLASTp via NCBI BLAST+ [62] with individual chains in the SSym structures as queries [60], and individual chains in the pre-training set as the database; for each SSym chain, we then considered its maximum similarity to any sequence in the pre-training set. We found that 9 Ssym proteins have similarity below 70% to the pre-training proteins (only 5 are below 50%, none are below 40%), and 10 proteins have similarity above 70% (comprising the two panels in Fig. 5B).

### Statistical comparison of model performances on classification metrics

#### Comparing models’ performances on the same tasks.

Figures 2 and 3 report classification performance (precision, recall, F1, etc.) for predicting the stability effects of mutations across models. Figures S5 and S3 report pairwise significance tests (p-values) for differences in these metrics using permutation tests, with the null hypothesis that two models’ predictions are exchangeable. To compute these p-values, for each model pair, we generated permuted prediction sets by swapping paired prediction between models with probability 0.5, and repeat this procedure for 1000 random seeds. The p-value is the fraction of permutations in which the metric difference between the permuted sets is at least as large (≥) as the observed difference. We correct the computed p-values for multiple comparisons using Holm–Bonferroni across all model pairs within each metric (i.e., within each panel in Figs. S5, S3).

#### Comparing a model’s performance across tasks.

Figure 3 reports classification performance (precision, recall, F1, etc.) for predicting mutation stability effects across mutation size categories (tasks). Figure S4 reports, for each model, pairwise significance tests for differences in these metrics between tasks. We estimate p-values via bootstrap resampling: for each task pair, we repeatedly (1000 seeds) draw bootstrap samples of predictions within each task, recompute the metric difference, and define the p-value as the fraction of bootstrap replicates in which the resampled metric difference is at least as large (≥) as the observed metric difference. We apply Holm–Bonferroni correction across all task pairs within each metric.

#### Significance of the number of retrieved antigen-stabilizing mutations by different models.

In Figures 6, 7 and Table S4, we report, for each model, the number of antigen-stabilizing mutations retrieved (x) out of a total of N=33 experimentally verified such mutations. Retrieval is based on amino-acid ranking: a mutation is counted as retrieved if (i) the mutant amino acid is ranked better than the wild type $\left(r_{\text{mt}}<r_{\text{wt}}\right)$, and (ii) that its rank is below or equal to a certain threshold R, with R=3 for “strongly suggested” and R=6 for “moderately suggested.” We assess significance in the number of retrieved antigen-stabilizing mutations by a given model with a one-sided binomial test: for each model, the p-value is the probability under a null model of recovering at least x successes in N trials with permutation success probability $p_{\text{null}}$, i.e., $p=1-\mathrm{BinomCDF}\left(x-1,N,p_{\text{null}}\right)$. We adjust p-values for multiple testing using Holm–Bonferroni, and report the resulting p-values in Figs. S15 and S15.

To compute the p-values, we consider two null models:

- **Random null.** For each mutation, we sample ranks for the mutant and wild type uniformly without replacement: $r_{\text{mt}}\sim \mathrm{Unif}\{1,\ldots ,20\}$ and $r_{\text{wt}}\sim \mathrm{Unif}\{1,\ldots ,20\}\backslash r_{\text{mt}}$. A mutation is a “success” if $r_{\text{mt}}<r_{\text{wt}}$ and $r_{\text{mt}}\leq R$, which occurs with probability $p_{null}=\frac{1}{19\times 20}\sum_{i=1}^{R}\left(20-i\right)$.
- **BLOSUM62 null.** We simply set $p_{null}=x_{B62}/N$, where $x_{B62}$ is the number of mutations with BLOSUM62 rank $r_{\text{mt}}^{B62}\leq R$. We note that the rank of the wild-type is always 1 for BLOSUM62, so we omit the condition that the rank of the mutant has to be lower than the rank of the wild-type, which is instead applied to all other models, including the random null model.

### Using Rosetta to score antigen-stabilizing mutations

In Figures 6, 7, S14 and Tables S4, S5, we reported the Rosetta scores for the verified antigen-stabilizing mutations. To compute these scores, we used the PyRosetta software [34] to model protein structures, with wild-type structures serving as template. For each target sequence containing a single point mutation, we threaded the mutant sequence onto all chains of the oligomeric template. Each resulting model was then subjected to a high-resolution refinement protocol using Rosetta’s FastRelax application. This protocol involved five cycles of side-chain rotamer repacking followed by gradient-based energy minimization of backbone (ϕ,ψ) and side-chain (χ) torsion angles, guided by the ref2015_cart all-atom energy function. To improve conformational sampling, we repeated the threading-and-relaxation procedure 20 times per mutation. For each mutant, we report the mean Rosetta Energy Unit (REU) score of the five lowest-energy (most favorable) relaxed models.

## Supplementary Material

Supplement 1

## Figures and Tables

**Table 1:** Benchmark on predicting mutational effects on protein-protein binding in SKEMPI. The Spearman/Pearson correlations between model-predicted effects of single point mutations and experimental values from the SKEMPI v2.0 dataset [51] are reported both across mutations within each structure individually (“per-structure” correlations), and over mutations pooled across all complexes (“overall” correlations).

Method	Per-Struct. Pearson	Per-Struct. Spearman	Overall Pearson	Overall Spearman
Rosetta* [46]	0.328	0.299	0.311	0.347
FoldX* [47]	0.391	0.364	0.356	0.351

DDGPred* [48]	0.371	0.343	0.652	0.439
ESM-IF* [49]	0.231	0.209	0.296	0.287
MIF-Net.* [33]	0.395	0.348	0.667	0.480
RDE-Net.* [33]	0.469	0.433	0.642	0.527

Vanilla Pythia PPI^†^ [50]	0.478	0.449	0.709	0.537
Pythia-PPI^†^ [50]	0.565	0.527	0.785	0.637


ProteinMPNN 0.02	0.281	0.282	0.331	0.315
ProteinMPNN 0.30	0.270	0.255	0.334	0.289
HERMES-*fixed* 0.00	0.306	0.287	0.285	0.272
HERMES-*fixed* 0.50	0.317	0.308	0.291	0.286
HERMES-*amortized* 0.00	0.231	0.241	0.290	0.242
HERMES-*amortized* 0.50	0.222	0.239	0.276	0.222
HERMES-*fixed* 0.00 + cDNA117k	0.347	0.331	0.380	0.342
HERMES-*fixed* 0.50 + cDNA117k	0.305	0.294	0.344	0.288

HERMES-*fixed* 0.00 + SKEMPI Easy	0.471	0.433	0.578	0.476
HERMES-*fixed* 0.50 + SKEMPI Easy	0.430	0.389	0.512	0.420
HERMES-*fixed* 0.00 + SKEMPI Medium	0.472	0.430	0.576	0.466
HERMES-*fixed* 0.50 + SKEMPI Medium	0.407	0.368	0.497	0.403
HERMES-*fixed* 0.00 + SKEMPI Difficult	0.435	0.398	0.395	0.380
HERMES-*fixed* 0.50 + SKEMPI Difficult	0.399	0.359	0.328	0.322

Entries marked with * are taken from ref. [33] and, for machine learning-based methods (all except Rosetta and FoldX), were obtained using 3-fold cross-validation over SKEMPI complexes. Entries marked with † are taken from ref. [50], and were obtained via 5-fold coss validation on the SKEMPI complex structures. We evaluate HERMES using three train/test splits defined from SKEMPI metadata, with increasing difficulty: (i) *Easy*, a random split; (ii) *Medium*, which groups sites with similar binding sites (hold-out proteins) into the same split; (iii) *Difficult*, which groups sites from the same held-out protein types (functional classes) into the same split (see Methods for details). The HERMES models with most comparable training procedure to the other machine learning models are those fine-tuned on the *Easy* split, for which we used 3-fold cross-validation with splits defined by PDB complex. Note that, similar to HERMES, the machine learning models in ref. [33] were fine-tuned on SKEMPI $\text{ΔΔ}G_{\text{binding}}$ labels only, whereas Pythia-PPI [50] was trained on a mixture of SKEMPI binding labels and FireProtDB stability labels [52] and further refined via self-distillation.
