# Supplementary material for: HERMES: Holographic Equivariant neuRal network model for Mutational Effect and Stability prediction
Source: bioRxiv. 2026 Jan 15:2024.07.09.602403. Originally published 2024 Jul 13. Preprint. [Version 4] doi: 10.1101/2024.07.09.602403 (PMC11257601; doi:10.1101/2024.07.09.602403)
Supplement: Supplement 1 [file NIHPP2024.07.09.602403v4-supplement-1.pdf]

bioRxiv preprint doi: https://doi.org/10.1101/2024.07.09.602403; this version posted January 15, 2026. The copyright holder for this preprint (which was not certified by peer review) is the author/funder, who has granted bioRxiv a license to display the preprint in perpetuity. It is made available under a CC-BY 4.0 International license.

**Model**	**Accuracy** Pyrosetta pre-processing	**Accuracy** Biopython pre-processing
HERMES-*fixed* 0.00	0.73	0.75
HERMES-*fixed* 0.50	0.64	0.65
HERMES-*amortized* 0.00	0.55	0.47
HERMES-*amortized* 0.50	0.50	0.44
HERMES-*fixed* 0.00 + Ros	0.41	0.40
HERMES-*fixed* 0.50 + Ros	0.38	0.37
HERMES-*fixed* 0.00 + cDNA117k	0.47	0.45
HERMES-*fixed* 0.50 + cDNA117k	0.39	0.38
HERMES-*amortized* 0.00 + cDNA117k	0.37	-
HERMES-*amortized* 0.50 + cDNA117k	0.34	-
HERMES-*fixed* 0.00 + cDNA117k train ESMFold	0.46	0.49
HERMES-*fixed* 0.50 + cDNA117k train ESMFold	0.40	0.40
HERMES-*fixed* Untr. 0.00 + cDNA117k	0.09	-
HERMES-*fixed* Untr. 0.50 + cDNA117k	0.08	-

Table S1 Accuracy of HERMES models on wildtype amino-acid classification on all sites across 40 CASP12 test proteins. Accuracy is defined as proportion of sites for which the wild-type amino acid is predicted with the highest probability among all 20 canonical amino acids. Model names indicate the architecture, the coordinate-noise amplitude used, and when applicable, the fine-tuning dataset (listed after "+"); *Untr.* is short for *Untrained*, indicating models that had no pre-training and were instead only trained on stability effects. Accuracy is reported for the two pre-processing schemes (with PyRosetta and Biopython) used in HERMES.

**Model**	**hh:mm:ss**
HERMES-*fixed* 0.50	00:11:23
HERMES-*amortized* 0.50	00:11:23
HERMES-*relaxed* 0.50	12:13:20

Table S2 Inference speed of HERMES models on the T2837 dataset. Runtimes are reported in hours (hh), minutes (mm), and seconds (ss) for inference on the T2837 dataset, which comprises 2837 mutation effects across 129 proteins. For HERMES-*fixed* and HERMES-*amortized*, the script 'mutation_effect_prediction_with_hermes.py' was used; for HERMES-*relaxed*, the script 'mutation_effect_prediction_with_hermes_with_relaxation.py' was used. Both scripts, along with the dataset 'csv' file, are available in our GitHub repository. All models were executed using a single CPU and a single A40 GPU.

bioRxiv preprint doi: https://doi.org/10.1101/2024.07.09.602403; this version posted January 15, 2026. The copyright holder for this preprint (which was not certified by peer review) is the author/funder, who has granted bioRxiv a license to display the preprint in perpetuity. It is made available under a CC-BY 4.0 International license.

Category	Description
Hydrophobic Property	1. Retention coefficient in TFA<br>2. Free energy of solution in water<br>3. Solvation free energy<br>4. Melting point<br>5. Number of hydrogen-bond donors<br>6. Number of full nonbonding orbitals<br>7. Partition energy<br>8. Hydration number<br>9. Retention coefficient in high performance liquid chromatography (HPLC), pH 7.4<br>10. Retention coefficient in HPLC, pH 2.1<br>11. Partition coefficient in thin-layer chromatography<br>12. Retention coefficient at pH 2<br>13. R_f for 1-N-(4-nitrobenzofurazono)-amino acids in ethyl acetate/pyridine/water<br>14. ΔG of transfer from organic solvent to water<br>15. Hydration potential or free energy of transfer from vapor phase to water<br>16. R_f, salt chromatography<br>17. $\log D$, partition coefficient at pH 7.1 for acetamide derivatives of amino acids in octanol/water<br>18. $\Delta G = RT \log f$, f = fraction buried/accessible amino acids in 22 proteins
Steric Property	19. Average volume of buried residue<br>20. Residue accessible surface area in tripeptide<br>21. Graph shape index<br>22. Normalized van der Waals volume<br>23. STERMIMOL length of the side chain<br>24. STERMIMOL minimum width of the side chain<br>25. STERMIMOL maximum width of the side chain<br>26. Average accessible surface area<br>27. Distance between C_α and centroid of side chain<br>28. Side-chain angle θ<br>29. Side-chain torsion angle ϕ<br>30. Radius of gyration of side chain<br>31. Van der Waals parameter R_0<br>32. Van der Waals parameter ε<br>33. Refractivity<br>34. Value of θ (i)<br>35. Substituent van der Waals volume
Electronic Property	36. αCH chemical shifts<br>37. αNH chemical shifts<br>38. A parameter of charge transfer capability<br>39. A parameter of charge transfer donor capability<br>40. Nuclear magnetic resonance (NMR) chemical shift of α carbon<br>41. Localized electrical effect<br>42. Positive charge<br>43. Negative charge<br>44. Polarity<br>45. Net charge<br>46. Amphipathicity index<br>47. Isoelectric point<br>48. Electron-ion interaction potential values<br>49. $\mathrm{pK}_{\mathrm{NH}_2}$ (NH_2 on C_α)<br>50. $\mathrm{pK}_{\mathrm{COOH}}$ (COOH on C_α)

Table S3 Table of amino-acid properties used for comparison with substitution matrices. Values associated with each amino acid are listed in ref. [39].

bioRxiv preprint doi: https://doi.org/10.1101/2024.07.09.602403; this version posted January 15, 2026. The copyright holder for this preprint (which was not certified by peer review) is the author/funder, who has granted bioRxiv a license to display the preprint in perpetuity. It is made available under a CC-BY 4.0 International license.

antigen PDB id	stabilized name	mutation	BLOSUM62 score ; rank	Rosetta	Protein MPNN 0.30	Thermo MPNN + Megascale	HERMES *-fixed* 0.50	HERMES *-amortized* 0.50	HERMES *-fixed* 0.50 + cDNA117k	HERMES *-fixed* 0.50 + Megascale
				$r_{wt} \to r_{mt}$	$r_{wt} \to r_{mt}$	$r_{wt} \to r_{mt}$	$r_{wt} \to r_{mt}$	$r_{wt} \to r_{mt}$	$r_{wt} \to r_{mt}$	$r_{wt} \to r_{mt}$
RSV-F 4JHW	Cav1 [9]	S190F	-2 ; 16	$19 \to 11$	$4 \to 7$	$14 \to 3$	$4 \to 8$	$11 \to 4$	$12 \to 3$	$13 \to 3$
		V207L	1 ; 3	$9 \to 4$	$2 \to 7$	$6 \to 5$	$2 \to 3$	$3 \to 2$	$3 \to 2$	$3 \to 2$
	Uncl. [43]	S215P	-1 ; 13	$13 \to 1$	$1 \to 14$	$10 \to 20$	$10 \to 15$	$6 \to 3$	$16 \to 19$	$4 \to 20$
	TriC [9]	D486H	-1 ; 7	$9 \to 2$	$1 \to 10$	$1 \to 8$	$5 \to 4$	$10 \to 4$	$20 \to 9$	$18 \to 9$
		E487Q	2 ; 3	$2 \to 9$	$1 \to 13$	$1 \to 12$	$2 \to 3$	$4 \to 5$	$11 \to 12$	$14 \to 12$
		F488W	1 ; 3	$15 \to 17$	$1 \to 14$	$3 \to 1$	$2 \to 6$	$1 \to 13$	$1 \to 3$	$1 \to 2$
		D489H	-1 ; 7	$5 \to 2$	$3 \to 14$	$5 \to 12$	$11 \to 8$	$18 \to 15$	$20 \to 11$	$17 \to 12$
HA 7VDF	Universal-HA [12]	H355W	-2 ; 12	$5 \to 1$	$5 \to 1$	$4 \to 2$	$4 \to 3$	$7 \to 2$	$5 \to 3$	$8 \to 3$
		K380I	-3 ; 19	$17 \to 1$	$9 \to 1$	$11 \to 1$	$8 \to 4$	$14 \to 3$	$13 \to 2$	$17 \to 2$
		E432I	-3 ; 19	$14 \to 9$	$12 \to 2$	$13 \to 2$	$9 \to 4$	$15 \to 5$	$12 \to 3$	$14 \to 3$
hMPV-F 5WB0	M104 [10]	L130D	-4 ; 19	$15 \to 3^\dagger$	$8 \to 9$	$4 \to 10$	$5 \to 9$	$12 \to 2$	$8 \to 2$	$10 \to 4$
		A159L	-1 ; 10	$6 \to 1^\dagger$	$1 \to 12$	$3 \to 5$	$1 \to 7$	$2 \to 4$	$7 \to 3$	$5 \to 3$
		V203I	3 ; 2	$4 \to 3^\dagger$	$1 \to 2$	$1 \to 2$	$1 \to 2$	$2 \to 1$	$3 \to 1$	$2 \to 1$
		V430Q	-2 ; 10	$12 \to 3^\dagger$	$5 \to 4$	$12 \to 6$	$8 \to 1$	$10 \to 5$	$5 \to 12$	$10 \to 8$
		V449D	-3 ; 16	$13 \to 8^\dagger$	$13 \to 3$	$16 \to 2$	$11 \to 2$	$15 \to 2$	$18 \to 2$	$19 \to 6$
	MPV-2cREKR [11]	V112R	-3 ; 19	$6 \to 13^\dagger$	$8 \to 1$	$2 \to 1$	$2 \to 9$	$8 \to 3$	$4 \to 11$	$5 \to 10$
		D209E	2 ; 2	$8 \to 1^\dagger$	$11 \to 1$	$12 \to 4$	$3 \to 1$	$14 \to 3$	$15 \to 6$	$16 \to 7$
		V231I	3 ; 2	$3 \to 1^\dagger$	$3 \to 1$	$3 \to 1$	$2 \to 1$	$2 \to 1$	$3 \to 1$	$3 \to 1$
		E453P	-1 ; 10	$10 \to 2^\dagger$	$1 \to 20$	$1 \to 2$	$8 \to 19$	$13 \to 1$	$17 \to 16$	$19 \to 6$
	Uncl. [43]	E80D	2 ; 2	$18 \to 13$	$3 \to 1$	$13 \to 18$	$1 \to 12$	$7 \to 17$	$15 \to 19$	$14 \to 18$
		V155P	-2 ; 13	$10 \to 20$	$4 \to 20$	$1 \to 20$	$1 \to 19$	$1 \to 20$	$2 \to 20$	$4 \to 20$
DENV-E 1OAN	SC12 [13]	S29K	0 ; 6	$4 \to 3^\dagger$	$3 \to 1$	$7 \to 2$	$1 \to 12$	$2 \to 5$	$6 \to 12$	$9 \to 14$
		T33V	0 ; 3	$9 \to 4^\dagger$	$3 \to 1$	$6 \to 1$	$3 \to 1$	$5 \to 1$	$10 \to 1$	$10 \to 1$
		A35M	-1 ; 13	$11 \to 4^\dagger$	$6 \to 1$	$10 \to 8$	$1 \to 12$	$1 \to 11$	$1 \to 7$	$6 \to 7$
		G106D	-1 ; 5	$18 \to 1^\dagger$	$11 \to 1$	$6 \to 11$	$8 \to 1$	$6 \to 2$	$20 \to 1$	$18 \to 1$
		A259W	-3 ; 20	$7 \to 1^\dagger$	$1 \to 3$	$4 \to 11$	$1 \to 20$	$1 \to 13$	$1 \to 2$	$2 \to 1$
		T262R	-1 ; 11	$15 \to 3^\dagger$	$1 \to 15$	$18 \to 7$	$3 \to 14$	$10 \to 3$	$16 \to 3$	$17 \to 4$
		F279W	1 ; 3	$3 \to 1^\dagger$	$4 \to 11$	$4 \to 1$	$1 \to 6$	$3 \to 7$	$1 \to 4$	$1 \to 2$
		T280P	-1 ; 13	$12 \to 2^\dagger$	$7 \to 10$	$9 \to 14$	$4 \to 19$	$6 \to 2$	$2 \to 20$	$8 \to 19$
SARS-Cov-2 6VSB	hexapro [44]	F817P	-4 ; 20	$3 \to 1$	$16 \to 1$	$1 \to 12$	$2 \to 16$	$3 \to 1$	$2 \to 18$	$2 \to 19$
		A892P	-1 ; 14	$16 \to 3$	$5 \to 1$	$2 \to 4$	$2 \to 1$	$4 \to 1$	$4 \to 1$	$5 \to 1$
		A899P	-1 ; 14	$8 \to 19$	$6 \to 8$	$7 \to 18$	$2 \to 6$	$4 \to 1$	$8 \to 15$	$5 \to 17$
		A942P	-1 ; 14	$5 \to 12$	$3 \to 1$	$2 \to 8$	$1 \to 4$	$2 \to 1$	$2 \to 1$	$2 \to 1$
	proportion of correctly and strongly suggested mutations			**20/33**	**15/33**	**11/33**	8/33	**19/33**	**15/33**	**13/33**
	proportion of correctly and at least moderately suggested mutations			**23/33**	**16/33**	14/33	11/33	**23/33**	**16/33**	**17/33**
	proportion of correctly and at least weakly suggested mutations			**27/33**	16/33	16/33	12/33	**24/33**	19/33	22/33

Table S4 Predicting antigen-stabilizing mutations with HERMES: extended results. Recall for different models (columns) is evaluated on 33 previously reported antigen-stabilizing mutations (rows) spanning five viral antigens. For each antigen, we list the PDB structure used for scoring and the publication(s) that originally reported the mutation. Mutations are specified as wild-type→mutant substitutions at the annotated site. Seven models are compared (columns). We additionally report the BLOSUM62 substitution score for each mutation and the mutant's rank among the 20 possible amino-acid substitutions for the wild-type residue (per BLOSUM62). For each model and mutation, predicted ranks of the wild-type and mutant amino acids are shown as $r_{wt} \to r_{mt}$. Dagger symbols (†) indicate mutations originally proposed as stabilizing by Rosetta-based pipelines in the source reference. When a model ranks the mutant better than the wild type ($r_{mt} < r_{wt}$), the cell is shaded by the prediction strength based on the value of r_{mt}: dark green, strongly suggested ($r_{mt} \leq 3$); light green, moderately suggested ($4 \leq r_{mt} \leq 6$); light yellow, weakly suggested ($r_{mt} \geq 6$). Column summaries report counts of strongly, at least moderately, and at least weakly suggested mutations (out of 33); **bold** indicates significance (p-value < 0.05) for the number of recalled mutations relative to a random null model (see Fig. S15 for p-values and Methods for details.) All structures were scored in their native multimeric states, generating symmetric partners when needed. ThermoMPNN's native mode predicts mutation effects only for monomers, ignoring multimeric assemblies even when present in the input structure. "Uncl." stands for uncleaved prefusion-closed state.

bioRxiv preprint doi: https://doi.org/10.1101/2024.07.09.602403; this version posted January 15, 2026. The copyright holder for this preprint (which was not certified by peer review) is the author/funder, who has granted bioRxiv a license to display the preprint in perpetuity. It is made available under a CC-BY 4.0 International license.

pdbID	# of monomer sites	HERMES GPU [seconds] all sites	HERMES CPU [seconds] all sites	Rosetta [CPU-hours] one site	Rosetta [CPU-hours] all sites
4JHW	449	57	112	150	67,350
7VDF	485	64	118	164	79,540
5WB0	442	43	154	151	66,742
1OAN	394	32	151	59	23,246
6VSB	968	69	265	156	151,008

Table S5 Execution times for saturation mutagenesis predictions on the viral antigens considered in this study. Executions times (in seconds) of HERMES apply to HERMES-*fixed* and HERMES-*amortized* models, regardless of whether zero-shot or fine-tuned. Times were computed when running the script `run_hermes_on_pdbfiles.py` providing as input the pdbfile as well as a single monomeric chain. A single CPU core with 64GB of memory was used, and a NVIDIA A40 GPU when applicable. For Rosetta, we computed times (in CPU-hours) for a single CPU core with 4 GBs of memory, and averaging 10 relaxation instances, which we consider the minimum number of instances for robust results. Times for all sites in the structure were extrapolated by multiplying the calculated average time for a single mutation by the number of monomeric sites.

antigen PDB id	stabilized name	mutation	mutation type	is synergistic	notes
RSV-F 4JHW	Cav1 [9]	S190F	cavity-filling	False	
		V207L	cavity-filling	False	
	Uncl. [43]	S215P	proline	False	
	TriC [9]	D486H	electrostatic	True	
		E487Q	electrostatic	True	
		F488W	cavity-filling	True	
		D489H	electrostatic	True	
HA 7VDF	Universal-HA [12]	H355W	cavity-filling	False	
		K380I	cavity-filling	False	
		E432I	cavity-filling	False	
hMPV-F 5WB0	M104 [10]	L130D	electrostatic	False	
		A159L	cavity-filling	False	
		V203I	cavity-filling	False	
		V430Q	electrostatic	False	
		V449D	electrostatic	False	
	MPV-2cREKR [11]	V112R	electrostatic	False	
		D209E		False	same charge, slightly different size: unclear
		V231I	cavity-filling	False	
		E453P	proline	False	
	Uncl. [43]	E80D		False	same charge, slightly different size: unclear
		V155P	proline	False	
DENV-E 1OAN	SC12 [13]	S29K	electrostatic	False	
		T33V	cavity-filling	False	
		A35M	cavity-filling	False	
		G106D	electrostatic	False	
		A259W	cavity-filling	True	
		T262R	electrostatic	True	
		F279W	cavity-filling	False	
		T280P	proline	False	
SARS-Cov-2 6VSB	hexapro [44]	F817P	proline	False	
		A892P	proline	False	
		A899P	proline	False	
		A942P	proline	False	

Table S6 Characteristics of antigen-stabilizing mutations. Hand-curated mutation types are listed for antigen-stabilizing mutations reported in Fig. 6 and Table S4. Cavity-filling mutations are defined as substitutions to hydrophobic residues that are larger than the wild-type when the wild-type is also hydrophobic. Electrostatic mutations are substitutions that change the residue's net charge. Proline mutations correspond to substitutions to proline. Synergistic mutations were identified through structural reasoning based on the spatial arrangement of mutations within the corresponding structure; see ref. [45] for a breakdown of mutation types considered in the structure-based vaccine design literature. "Uncl." stands for "Uncleaved Prefusion-Closed".

bioRxiv preprint doi: https://doi.org/10.1101/2024.07.09.602403; this version posted January 15, 2026. The copyright holder for this preprint (which was not certified by peer review) is the author/funder, who has granted bioRxiv a license to display the preprint in perpetuity. It is made available under a CC-BY 4.0 International license.

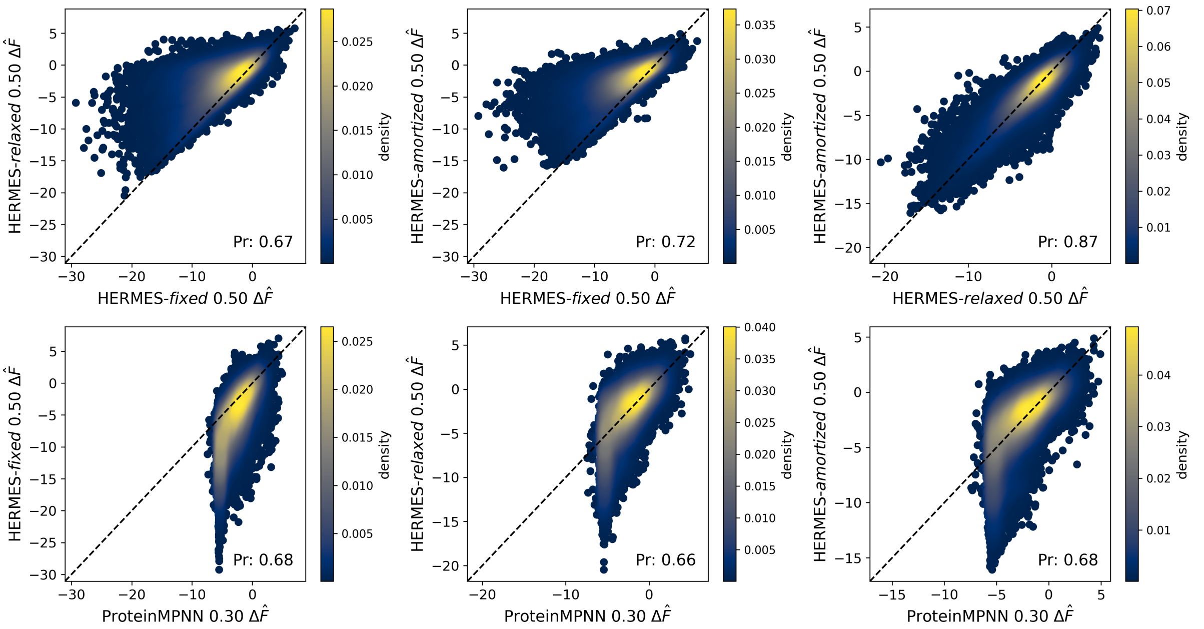

Figure S1 Comparison of zero-shot model predictions on the Megascale test set. For each substitution in the Megascale test set, the predicted change in amino acid propensity upon mutation ($\delta \log p$) is compared between two models in each panel. Color indicates local point density (blue denotes low density and yellow denotes high density). The reported "Pr" in each panel corresponds to the Pearson correlation coefficient between the predictions of the model pair. Model names indicate the architecture and the coordinate-noise amplitude used. $\Delta\hat{F}$ indicates the model's prediction, following Equations 1 and 2.

bioRxiv preprint doi: https://doi.org/10.1101/2024.07.09.602403; this version posted January 15, 2026. The copyright holder for this preprint (which was not certified by peer review) is the author/funder, who has granted bioRxiv a license to display the preprint in perpetuity. It is made available under a CC-BY 4.0 International license.

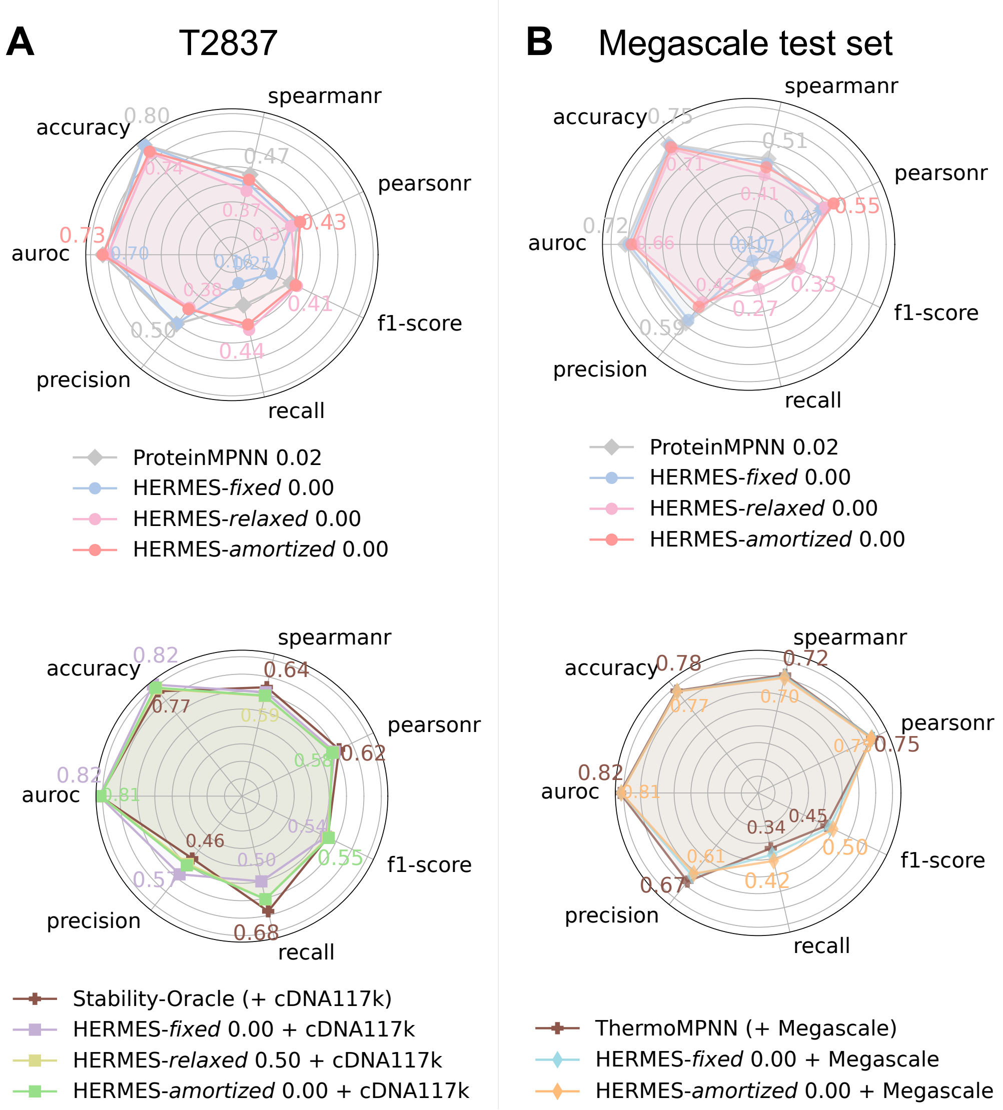

Figure S2 Predicting mutational effects on thermodynamic folding stability. Stabilizing-versus-destabilizing classification metrics are computed using $\Delta\Delta G < 0$ (experimental) and $\Delta \log p > 0$ (predicted) as cutoffs for stabilizing mutations. **(A)** Evaluation on the T2837 results: zero-shot models (top) and models fine-tuned on cDNA117k (bottom). **(B)** Evaluation on Megascale test set results: zero-shot models (top) and models fine-tuned on the Megascale training set (bottom). Model names indicate the architecture, the coordinate-noise amplitude used, and when applicable, the fine-tuning dataset (listed after "+"); *Untr.* is short for *Untrained*, indicating models that had no pre-training and were instead only trained on stability effects. Only models trained without coordinate noise are shown; the noise amplitude is indicated within each model name.

bioRxiv preprint doi: https://doi.org/10.1101/2024.07.09.602403; this version posted January 15, 2026. The copyright holder for this preprint (which was not certified by peer review) is the author/funder, who has granted bioRxiv a license to display the preprint in perpetuity. It is made available under a CC-BY 4.0 International license.

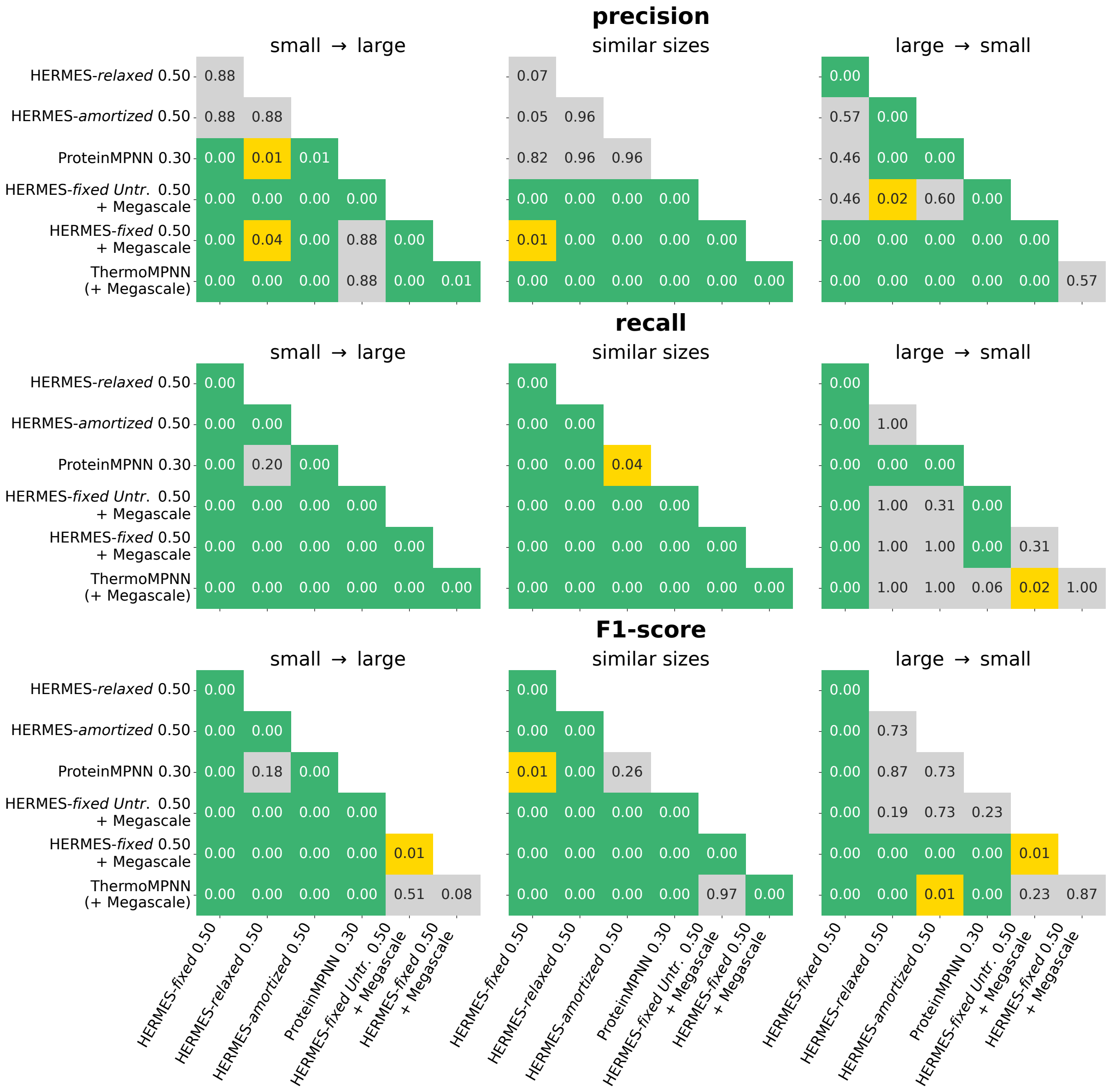

Figure S3 Statistical significance of model performance differences on stabilizing mutation identification. Shown are two-tailed p-values for differences in model performance on identifying stabilizing mutations across Megascale test set subsets. P-values correspond to the performance comparisons shown in Fig. 3. Green indicates strong statistical significance ($p < 0.01$), while yellow indicates weaker significance ($0.01 \leq p < 0.05$). P-values were computed using a permutation test and corrected for multiple comparisons using the Holm–Bonferroni procedure within each performance metric (see Methods for details).

bioRxiv preprint doi: https://doi.org/10.1101/2024.07.09.602403; this version posted January 15, 2026. The copyright holder for this preprint (which was not certified by peer review) is the author/funder, who has granted bioRxiv a license to display the preprint in perpetuity. It is made available under a CC-BY 4.0 International license.

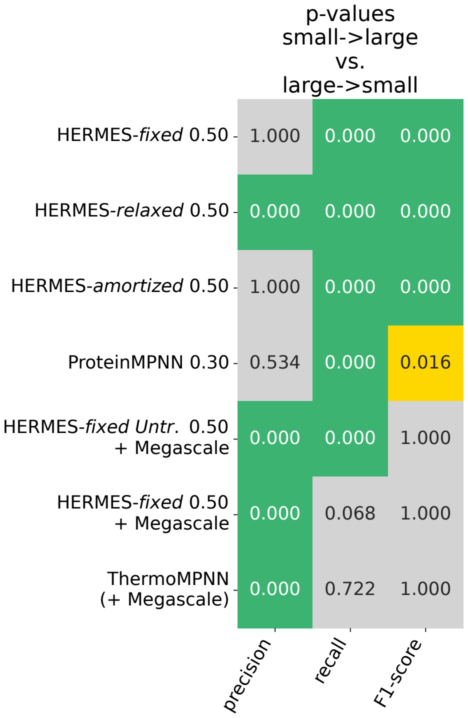

Figure S4 Statistical significance of within-model performance differences in identifying stabilizing mutations across mutational size classes. Shown are two-tailed P-values for within-model differences in performance when identifying stabilizing mutations from the small→large vs. large→small mutational subsets of the Megascale test set. P-values correspond to the performance comparisons shown in Fig. 3. Green indicates strong statistical significance ($p < 0.01$), while yellow indicates weaker significance ($0.01 \leq p < 0.05$). P-values were computed using a bootstrap test and corrected for multiple comparisons using the Holm–Bonferroni procedure within each performance metric (see Methods for details).

bioRxiv preprint doi: https://doi.org/10.1101/2024.07.09.602403; this version posted January 15, 2026. The copyright holder for this preprint (which was not certified by peer review) is the author/funder, who has granted bioRxiv a license to display the preprint in perpetuity. It is made available under a CC-BY 4.0 International license.

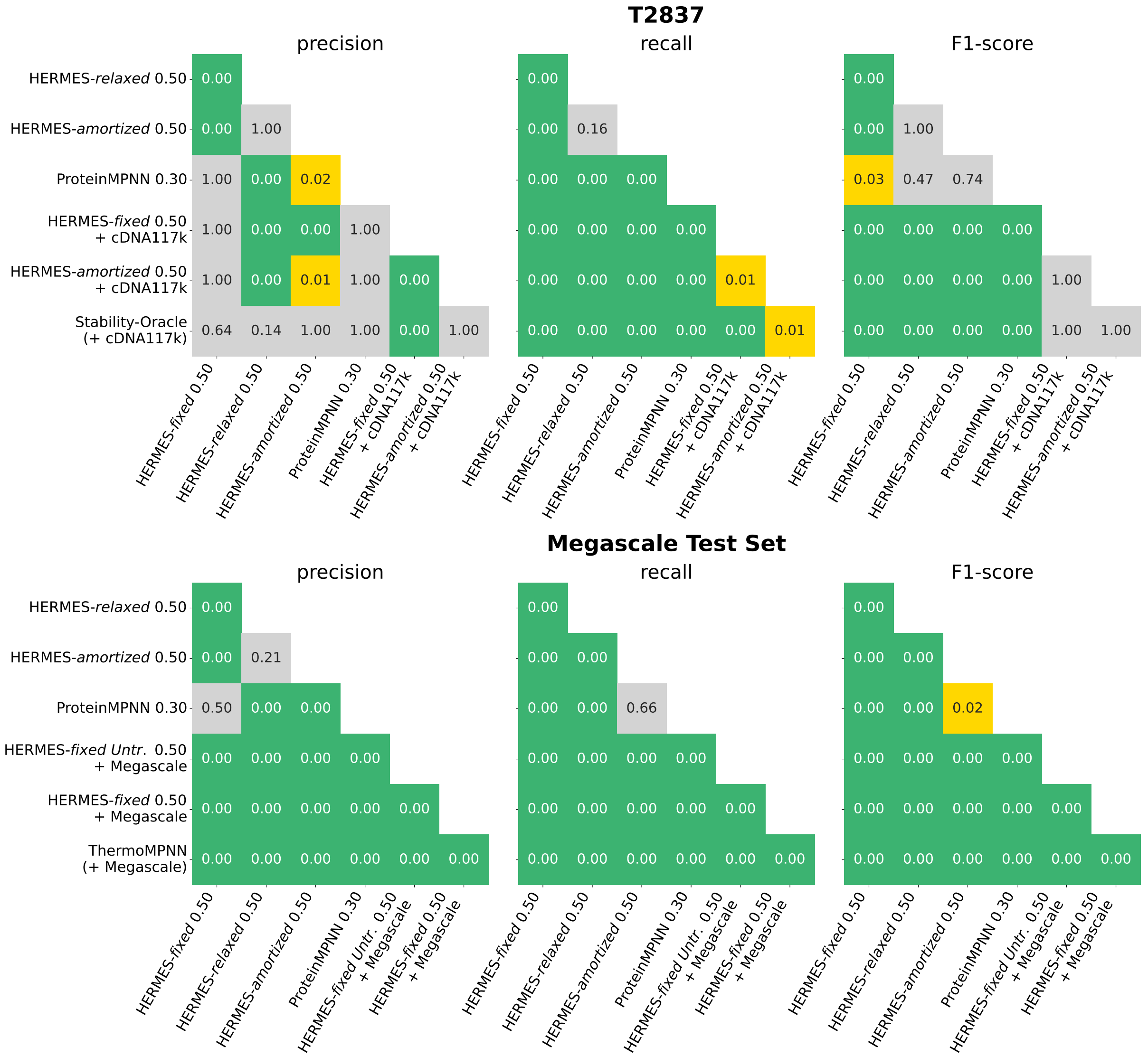

Figure S5 Statistical significance of model performance differences in identifying stabilizing mutations on the full test set. Shown are two-tailed p-values for differences in model performance when identifying stabilizing mutations on the full test set. P-values correspond to the performance comparisons shown in Fig. 2. Green indicates strong statistical significance ($p < 0.01$), while yellow indicates weaker significance ($0.01 \leq p < 0.05$). P-values were computed using a permutation test and corrected for multiple comparisons using the Holm–Bonferroni procedure within each performance metric (see Methods for details).

bioRxiv preprint doi: https://doi.org/10.1101/2024.07.09.602403; this version posted January 15, 2026. The copyright holder for this preprint (which was not certified by peer review) is the author/funder, who has granted bioRxiv a license to display the preprint in perpetuity. It is made available under a CC-BY 4.0 International license.

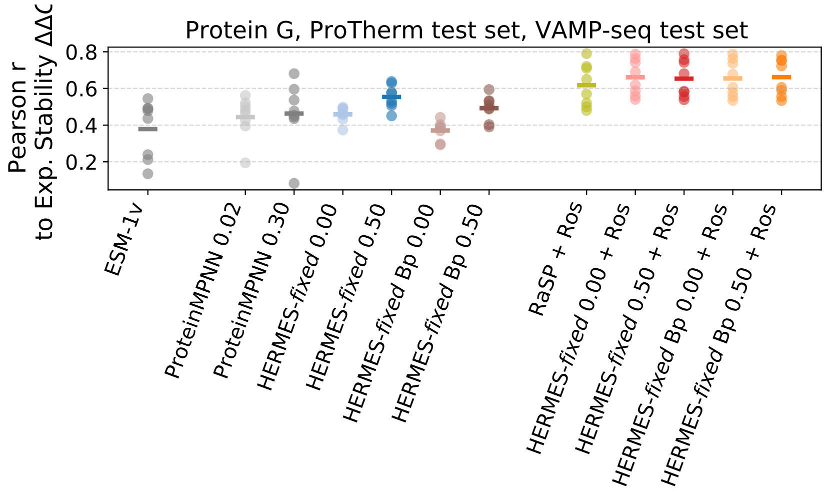

Figure S6 Pearson correlation between model predictions and experimental stability effects on the RaSP test set (8 proteins) [2]. Each dot represents one protein, and the horizontal bar indicates the mean correlation across proteins. Model labels specify the architecture, the coordinate-noise amplitude, and, when applicable, the fine-tuning dataset (denoted after "+"). "Bp" indicates the use of our open-source Biopython-based protein Pre-processing. See Methods for details on the RaSP dataset.

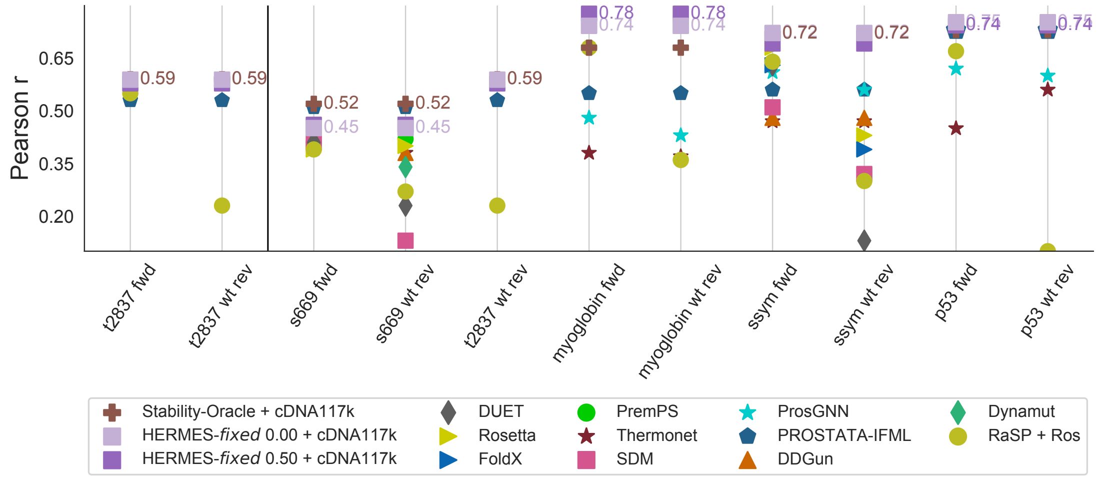

Figure S7 Pearson correlation between model predictions and experimental stability effects on the T2837 dataset and its subsets. Pearson correlation values for all models other than HERMES are taken from [27]. This figure closely replicates a figure from ref. [27], with the key difference that predictions for "reverse" mutations are computed here by conditioning on wild-type structures (denoted as "wt rev"). This distinction is made to avoid confusion with "reverse" mutation predictions computed on mutant structures in the Ssym dataset (Fig. 5). For each dataset (x-axis), we denote in text the performance of the HERMES models as well as that of the best-performing model overall.

bioRxiv preprint doi: https://doi.org/10.1101/2024.07.09.602403; this version posted January 15, 2026. The copyright holder for this preprint (which was not certified by peer review) is the author/funder, who has granted bioRxiv a license to display the preprint in perpetuity. It is made available under a CC-BY 4.0 International license.

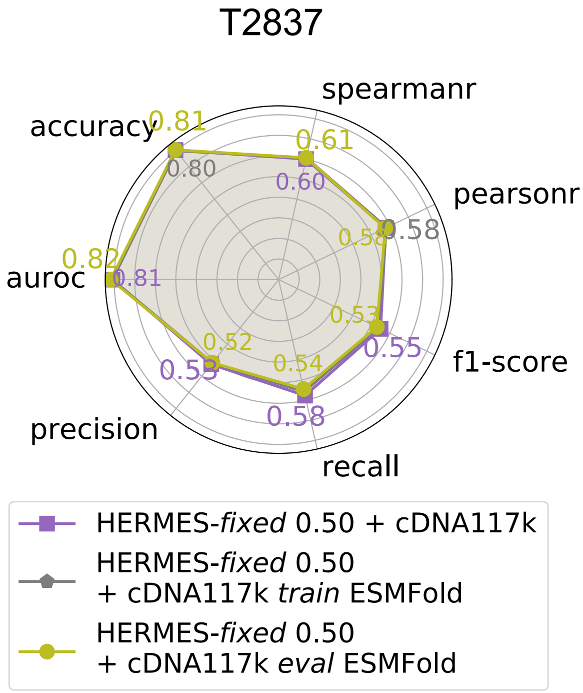

Figure S8 Predicting mutational effects on thermodynamic stability using ESMfold predicted structures for fine-tuning or testing. Stabilizing-versus-destabilizing classification metrics are computed using $\Delta\Delta G < 0$ (experimental) and $\Delta \log p > 0$ (predicted) as cutoffs for stabilizing mutations. We report results on the T2837 dataset, after fine-tuning models on cDNA117k. We consider models fine-tuned and evaluated on crystal structures (purple), models fine-tuned on ESMfold predicted structures and evaluated on crystal structures (grey, "*train* ESMfold" in the model name), and models fine-tuned on crystal structures and evaluated on ESMfold predicted structures (olive, "*eval* ESMfold" in the model name).

bioRxiv preprint doi: https://doi.org/10.1101/2024.07.09.602403; this version posted January 15, 2026. The copyright holder for this preprint (which was not certified by peer review) is the author/funder, who has granted bioRxiv a license to display the preprint in perpetuity. It is made available under a CC-BY 4.0 International license.

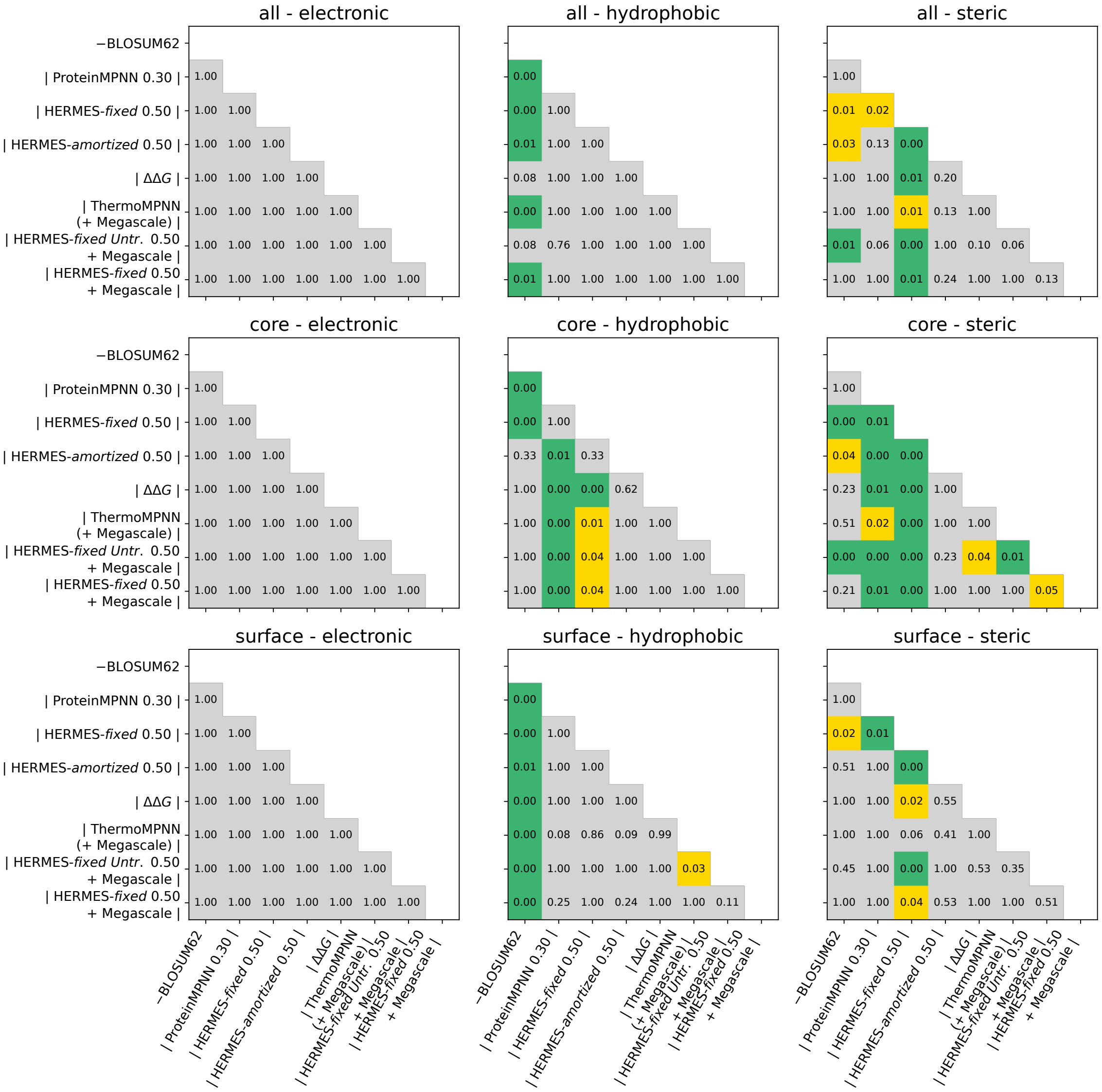

Figure S9 Significance testing results for Fig. 4B. Shown are p-values of two-tailed t-tests comparing the distributions of spearman correlations between each model's substitution matrix, and amino-acid properties of a particular class (electronic, hydrophobic, steric). The Holm-Bonferroni method was used to correct p-values for multiple testing error. Entries corresponding to pairs of distributions with p-value ≤ 0.01 are colored in green, p-value ≤ 0.05 are colored in yellow, and p-value > 0.05 are colored in gray.

bioRxiv preprint doi: https://doi.org/10.1101/2024.07.09.602403; this version posted January 15, 2026. The copyright holder for this preprint (which was not certified by peer review) is the author/funder, who has granted bioRxiv a license to display the preprint in perpetuity. It is made available under a CC-BY 4.0 International license.

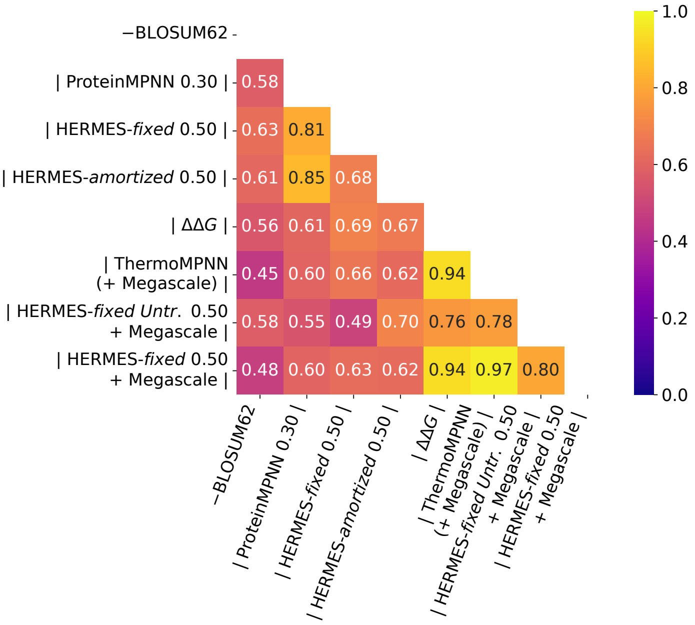

Figure S10 Spearman correlations between pairs of model-average substitution matrices M^{model}. The heatmaps for the underlying model-predicted substitution matrices are shown in Fig. 4.

bioRxiv preprint doi: https://doi.org/10.1101/2024.07.09.602403; this version posted January 15, 2026. The copyright holder for this preprint (which was not certified by peer review) is the author/funder, who has granted bioRxiv a license to display the preprint in perpetuity. It is made available under a CC-BY 4.0 International license.

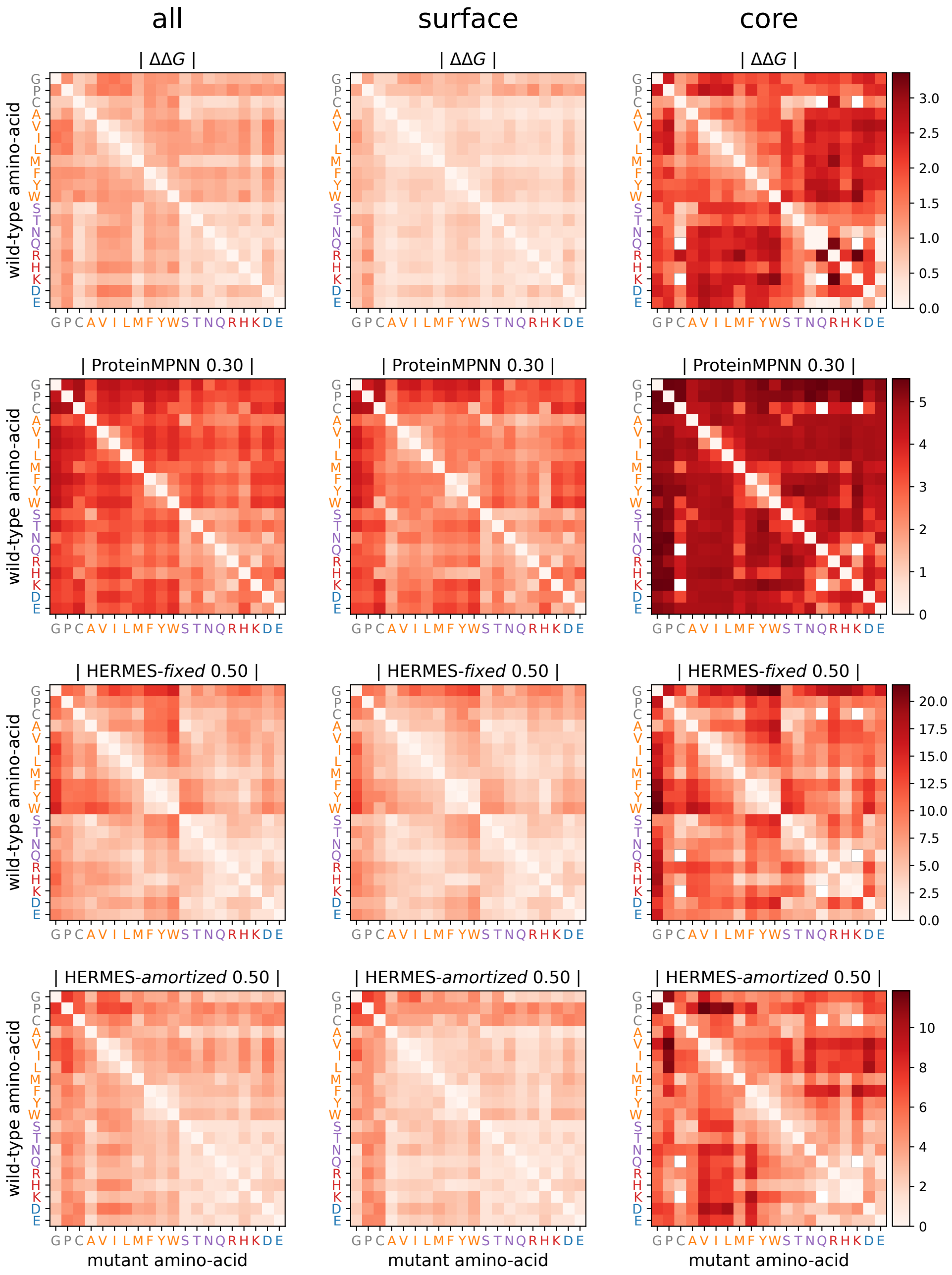

Figure S11 Model-averaged substitution matrices stratified by protein core and surface residues for zero-shot models. Shown are model-averaged substitution matrices M^{model} for different zero-shot models (rows 2-4), computed from subsets of sites in the Megascale test set. The first row shows the experimental matrices for mean $|\Delta\Delta G|$ values across mutation subsets. Columns correspond to all residues (left), core residues with solvent-accessible surface area SASA $< 1\mathring{A}^2$ (center), and surface residues with SASA $> 3\mathring{A}^2$ (right).

bioRxiv preprint doi: https://doi.org/10.1101/2024.07.09.602403; this version posted January 15, 2026. The copyright holder for this preprint (which was not certified by peer review) is the author/funder, who has granted bioRxiv a license to display the preprint in perpetuity. It is made available under a CC-BY 4.0 International license.

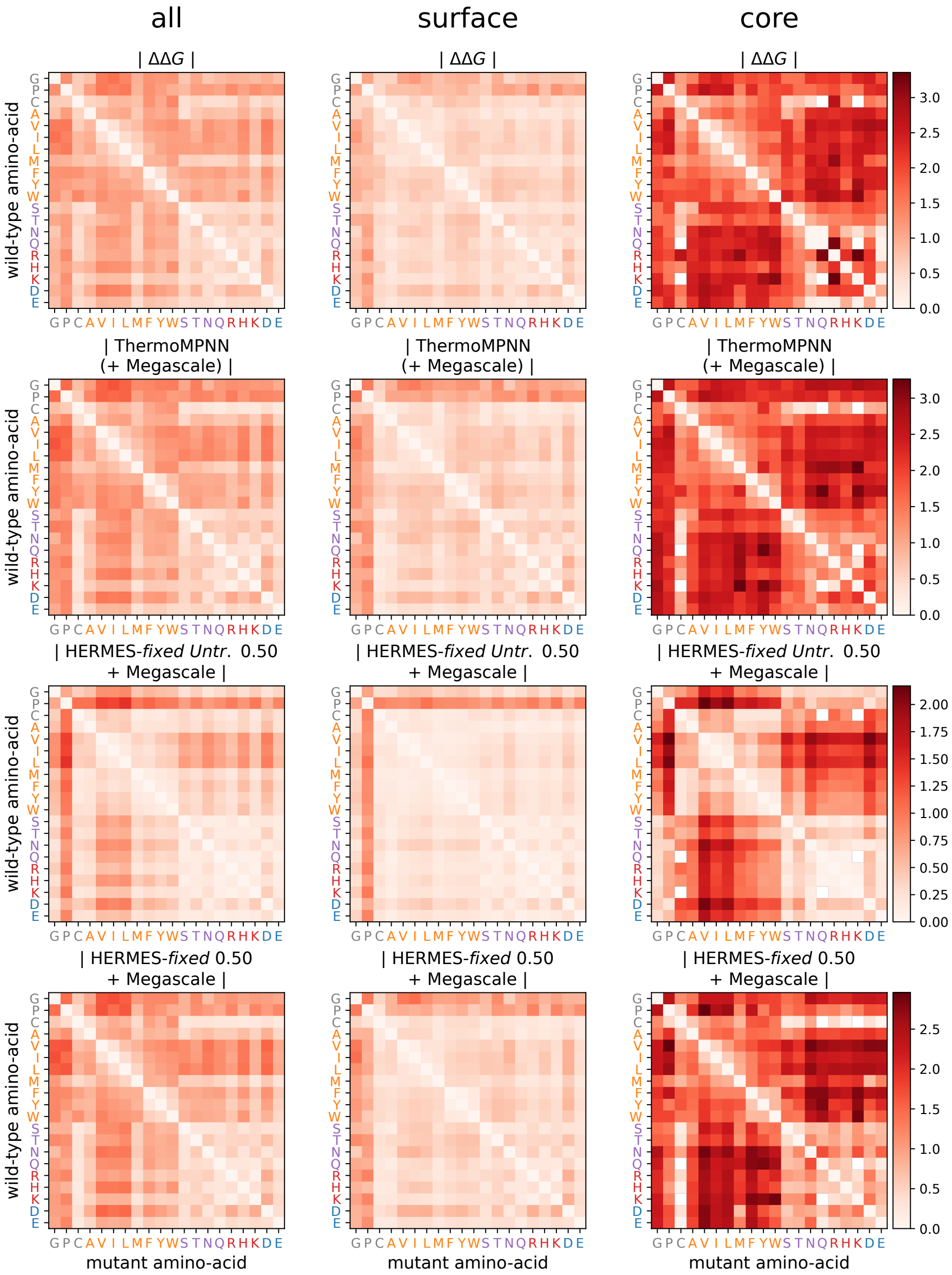

Figure S12 Model-averaged substitution matrices stratified by protein core and surface residues for stability fine-tuned models. Similar to Fig. S11 but for models fine-tuned on the Megascale dataset.

bioRxiv preprint doi: https://doi.org/10.1101/2024.07.09.602403; this version posted January 15, 2026. The copyright holder for this preprint (which was not certified by peer review) is the author/funder, who has granted bioRxiv a license to display the preprint in perpetuity. It is made available under a CC-BY 4.0 International license.

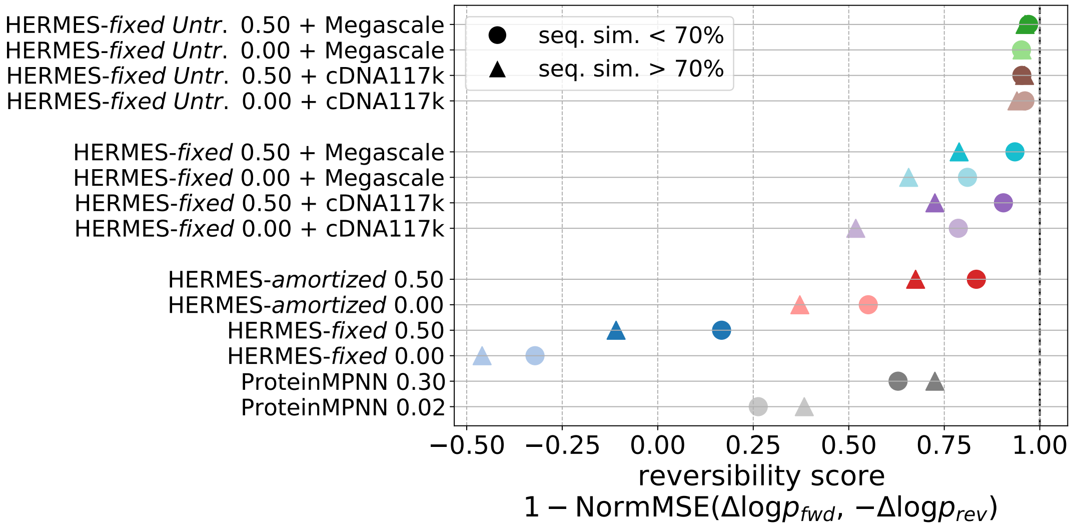

Figure S13 Model reversibility scores on the Ssym dataset. Reversibility is quantified as one minus the mean squared error between the forward mutational effect $\Delta \log p_{fwd}$ and the negated reverse effect $-\Delta \log p_{rev}$ for different models (rows), where model predictions are conditioned on the protein structure containing the outgoing amino acid; The resulting score is normalized to lie between -1 and 1, with higher values indicating a greater degree of reversibility: $1 - mean((\Delta \log p_{fwd} + \Delta \log p_{rev})^2)/(mean(\Delta \log p_{fwd}{}^2) + mean(\Delta \log p_{rev}{}^2))$.

bioRxiv preprint doi: https://doi.org/10.1101/2024.07.09.602403; this version posted January 15, 2026. The copyright holder for this preprint (which was not certified by peer review) is the author/funder, who has granted bioRxiv a license to display the preprint in perpetuity. It is made available under a CC-BY 4.0 International license.

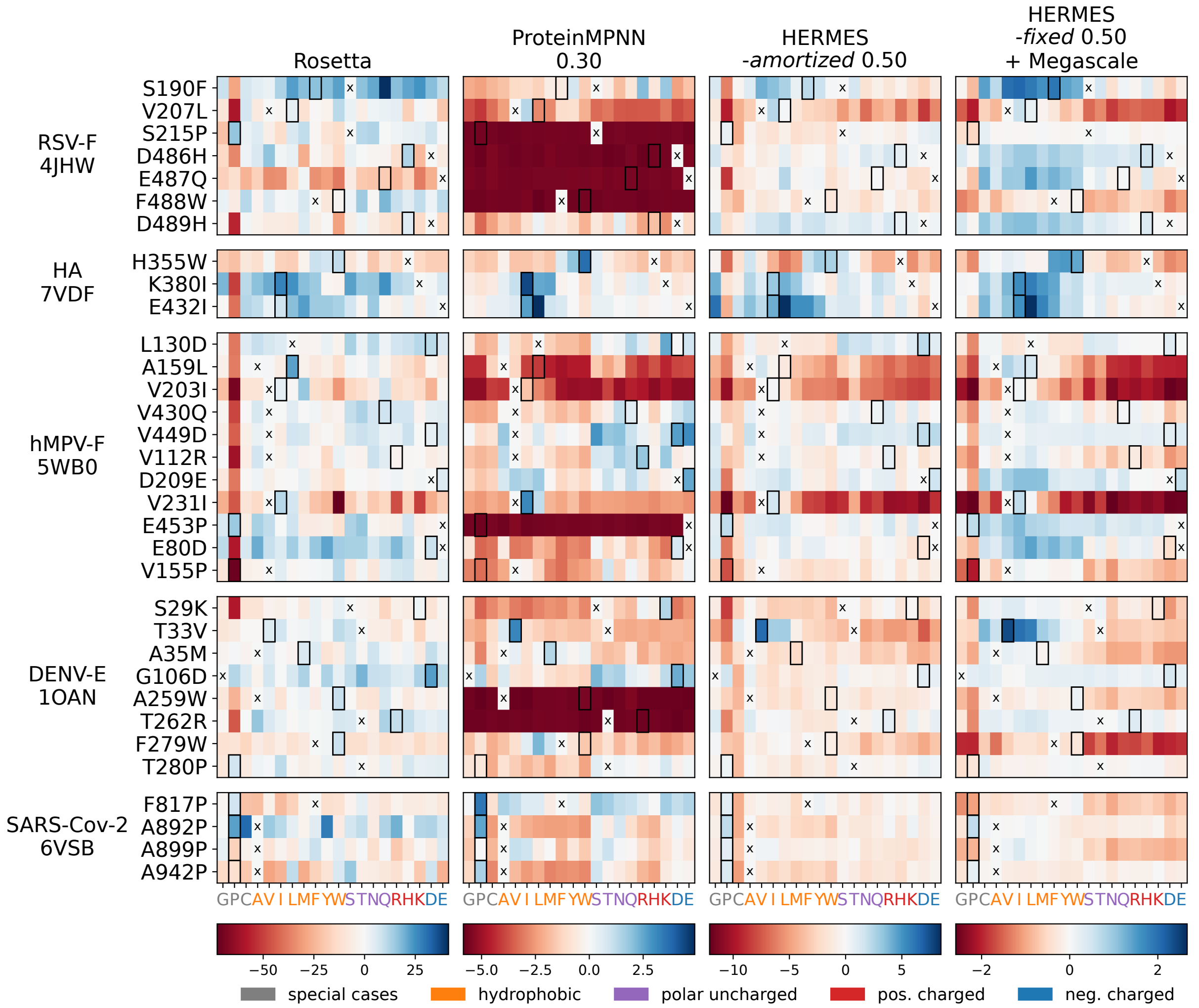

Figure S14 Model predictions for amino acid preferences at sites with known antigen-stabilizing mutations. Predictions from different models (columns) are shown for sites with known antigen-stabilizing mutations specified in Fig. 6 and Table S4. For each antigen (rows), predictions are computed using the protein structure corresponding to the PDB ID indicated on the left. Predictions are reported as changes in Rosetta Energy Units (REU) for Rosetta, and $\Delta \log p$ for ProteinMPNN and HERMES models. Wild-type amino acids are marked with centered crosses, while stabilizing mutant amino acids are indicated by dark borders. Amino acids are grouped by broad biochemical class (see legend at the bottom) and, within each class, ordered by increasing size (number of atoms).

bioRxiv preprint doi: https://doi.org/10.1101/2024.07.09.602403; this version posted January 15, 2026. The copyright holder for this preprint (which was not certified by peer review) is the author/funder, who has granted bioRxiv a license to display the preprint in perpetuity. It is made available under a CC-BY 4.0 International license.

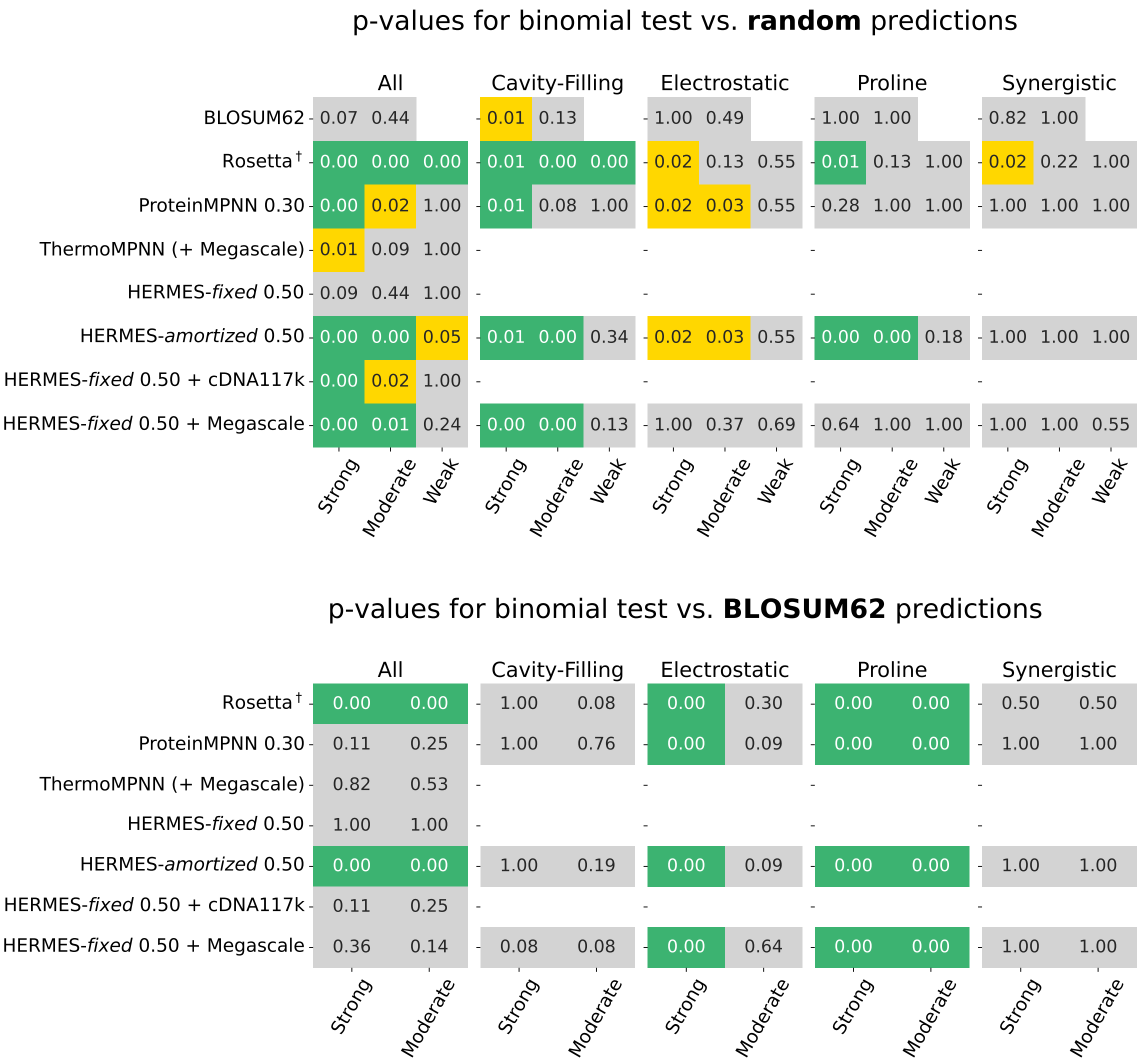

Figure S15 Statistical significance for the number of retrieved antigen-stabilizing mutations. Shown are p-values from binomial tests comparing the number of antigen-stabilizing mutations retrieved by each model (rows) against random expectation (top) and the BLOSUM62 predictions (bottom). These significance tests correspond to the results reported in Figs. 6, 7 and Table S4; see Methods for details of p-value computation.

bioRxiv preprint doi: https://doi.org/10.1101/2024.07.09.602403; this version posted January 15, 2026. The copyright holder for this preprint (which was not certified by peer review) is the author/funder, who has granted bioRxiv a license to display the preprint in perpetuity. It is made available under a CC-BY 4.0 International license.

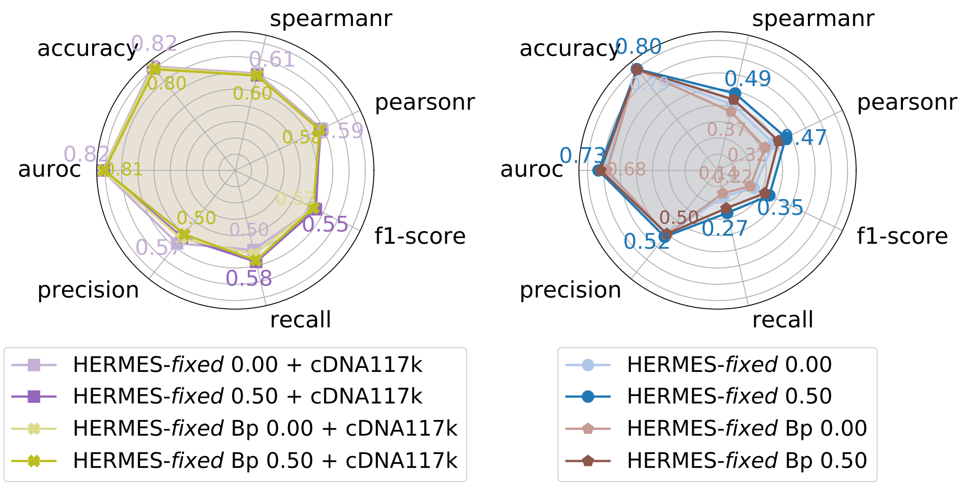

Figure S16 Comparison of PyRosetta and Biopython Pre-processing pipelines for predicting mutation stability effects on T2837. Classification accuracy metrics, analogous to those shown in Fig. 2, are reported for fine-tuned models (left) and zero-shot models (right). In each case, models trained using PyRosetta-based Pre-processing are compared with those using Biopython-based Pre-processing (denoted by "Bp" in the model name). Model labels specify the architecture, the coordinate-noise amplitude, and, when applicable, the fine-tuning dataset (listed after "+"). Consistent with results on the RaSP dataset (Fig. S6), Biopython-Pre-processed models show slightly reduced performance relative to PyRosetta-Pre-processed models; however, this difference becomes statistically insignificant after fine-tuning.

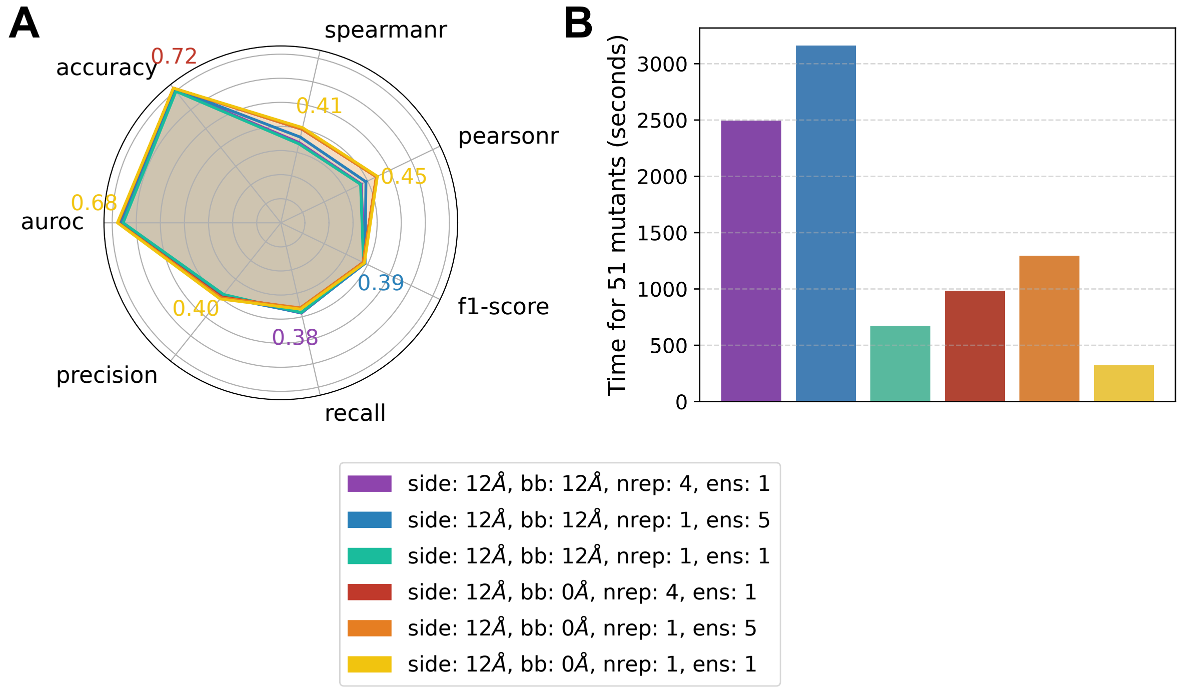

Figure S17 Ablation of PyRosetta fastrelax parameters for HERMES-*relaxed* 0.50 on the cDNA117k dataset. (A) Classification accuracy metrics, analogous to those shown in Fig. 2, are reported for HERMES-*relaxed* models evaluated on relaxed mutant structures using different PyRosetta fastrelax parameters (indicated by color). HERMES-*relaxed* scores a mutation as the log-probability difference between the mutant and wild-type amino acids. The wild-type log-probability is evaluated on the wild-type structure, while the mutant log-probability is evaluated on the wild-type structure after introducing the mutation and performing local relaxation. We use the PyRosetta fastrelax protocol and vary the following parameters, noting that the procedure is stochastic: (1) **side**, the distance cutoff for side-chain relaxation; (2) **bb**, the distance cutoff for backbone relaxation; (3) **nreps**, the number of protocol repetitions, with the lowest-energy conformation retained; (4) **ens**, the ensemble size, where predictions are averaged over relaxations obtained with different random seeds. **(B)** Inference speed for predicting mutational effects on 51 mutants across the same PyRosetta fastrelax parameters as in (A) (colors). A single NVIDIA A40 GPU and a single CPU with 64G of memory were used for all parameters.

bioRxiv preprint doi: https://doi.org/10.1101/2024.07.09.602403; this version posted January 15, 2026. The copyright holder for this preprint (which was not certified by peer review) is the author/funder, who has granted bioRxiv a license to display the preprint in perpetuity. It is made available under a CC-BY 4.0 International license.

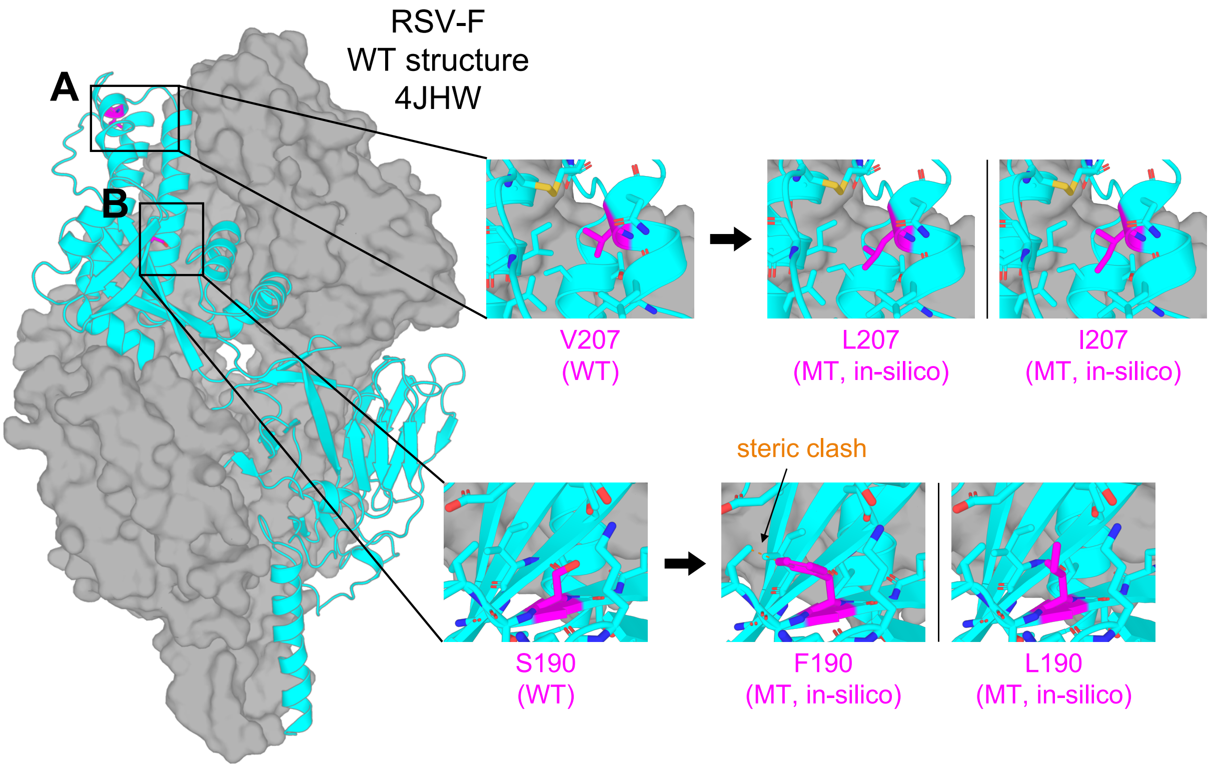

Figure S18 Structure of the wild-type pre-fusion RSV-F antigen highlighting observed and candidate antigen-stabilizing mutations. The wild-type structure is shown on the left (PDB ID: 4JHW). A single protomer of the trimer is displayed as a cyan cartoon, with the remaining protomers shown as a grey surface. WT denotes wild type and MT denotes mutant. Mutations labeled as "in silico" were introduced using PyMOL's Mutagenesis Wizard starting from the wild-type structure. Steric clashes (orange dashed lines) were identified using PyMOL's "find clashes" command, and polar contacts (yellow dashed lines) were identified using the corresponding PyMOL command. **(A)** The L207 mutant has been experimentally shown to stabilize the pre-fusion conformation [9] and appears to enhance intraprotomer packing. We speculate that the I207 mutant, which HERMES-*amortized* predicts to have a comparable ranking to L207 in Fig. S14, would pack similarly and may therefore represent an additional stabilizing mutation worth screening. **(B)** Mutant F190 is observed to be stabilizing [9], though it appears to slightly over-pack the region. We speculate that L190, which HERMES-*amortized* predicts to have a comparable ranking to F190 in Fig. S14, would provide a similarly stabilizing effect without over-packing the region.

bioRxiv preprint doi: https://doi.org/10.1101/2024.07.09.602403; this version posted January 15, 2026. The copyright holder for this preprint (which was not certified by peer review) is the author/funder, who has granted bioRxiv a license to display the preprint in perpetuity. It is made available under a CC-BY 4.0 International license.

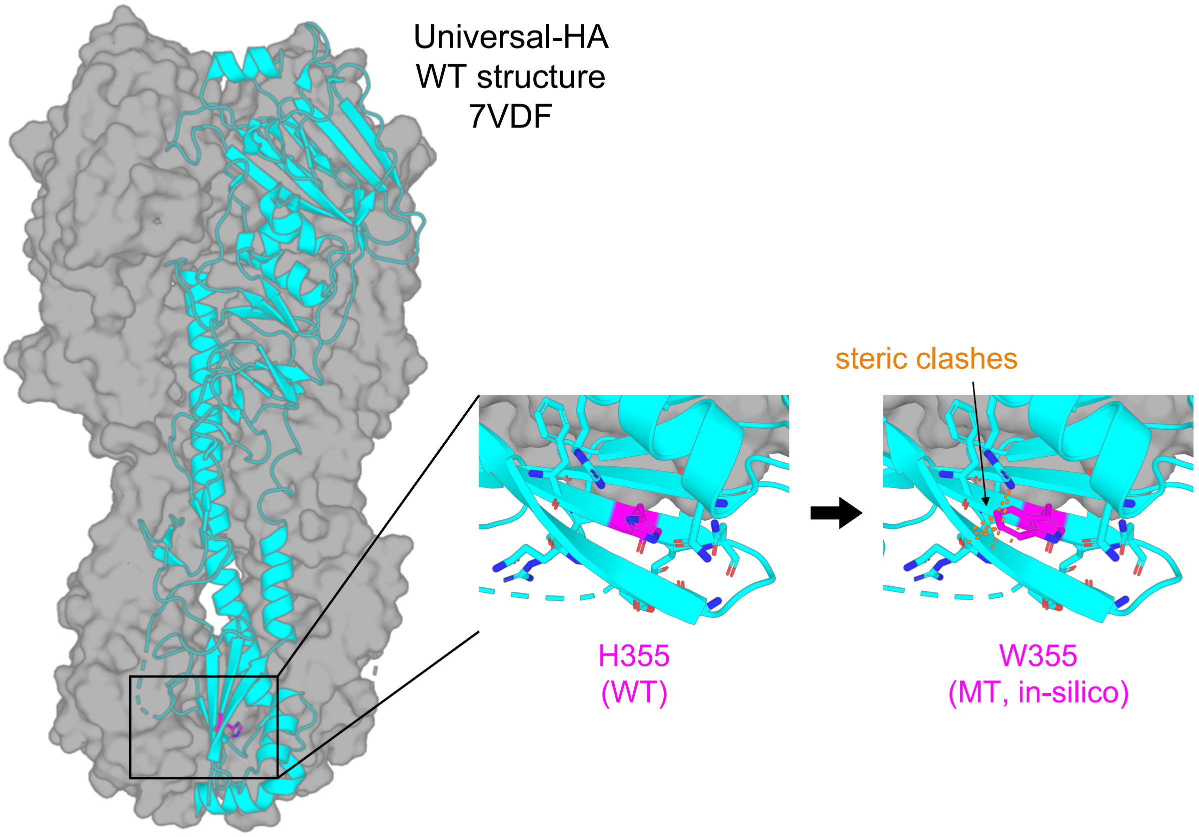

Figure S19 Structure of the wild-type pre-fusion Universal-HA antigen, with highlighted observed and candidate antigen-stabilizing mutations. The wild-type structure is shown on the left (PDB ID 7VDF). A single protomer of the trimer is shown as cyan cartoon; the other copies are shown as grey surface. WT denotes wild type and MT denotes mutant. Mutations labeled as "in silico" were introduced using PyMOL's Mutagenesis Wizard starting from the wild-type structure. Steric clashes (orange dashed lines) were identified using PyMOL's "find clashes" command, and polar contacts (yellow dashed lines) were identified using the corresponding PyMOL command. We highlight the H355W mutation which, despite stabilizing the pre-fusion conformation, exhibits substantial steric clashes in the wild-type structural context.

bioRxiv preprint doi: https://doi.org/10.1101/2024.07.09.602403; this version posted January 15, 2026. The copyright holder for this preprint (which was not certified by peer review) is the author/funder, who has granted bioRxiv a license to display the preprint in perpetuity. It is made available under a CC-BY 4.0 International license.

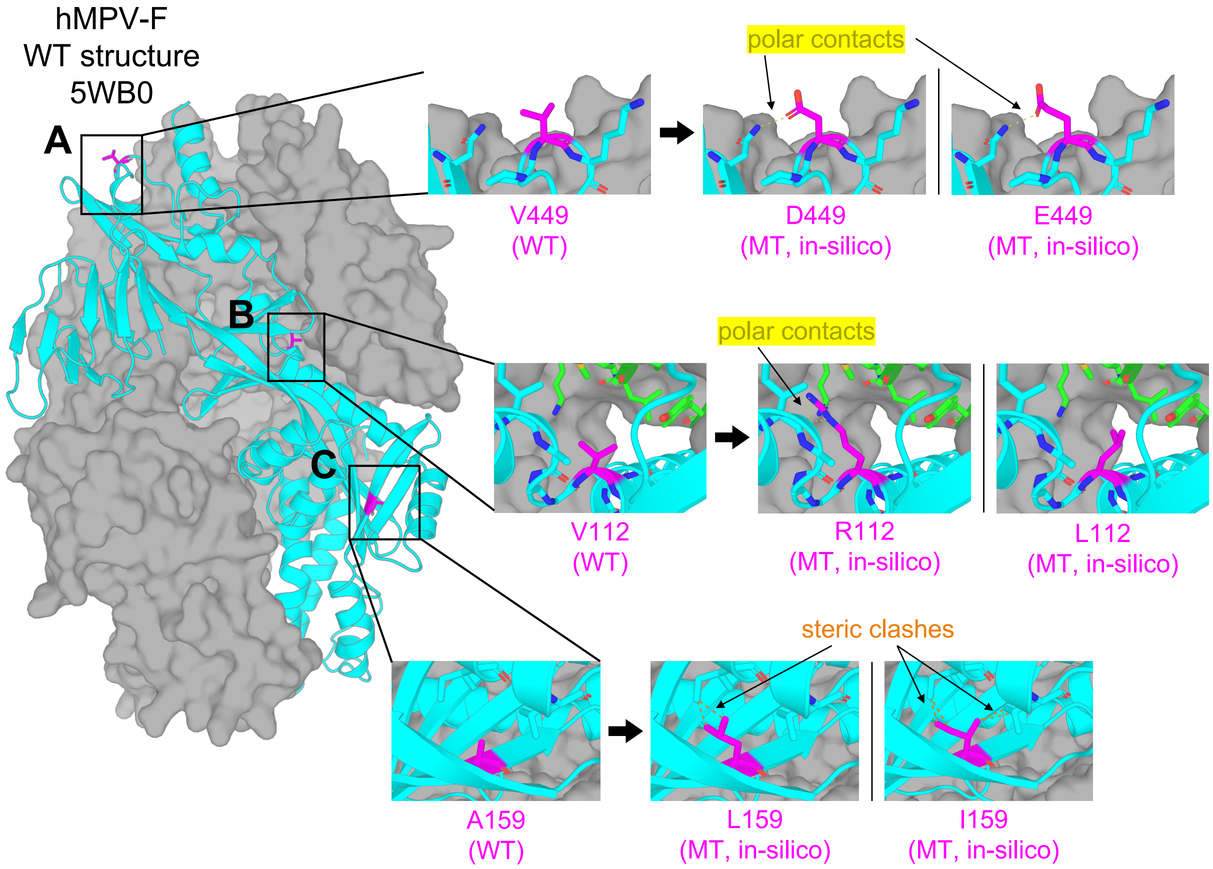

Figure S20 Structure of the wild-type pre-fusion hMPV-F antigen, with highlighted observed and candidate antigen-stabilizing mutations. The wild-type structure is shown on the left (PDB ID 5WB0). A single protomer of the trimer is shown as cyan cartoon; the other copies are shown as grey surface. WT denotes wild type and MT denotes mutant. Mutations labeled as "in silico" were introduced using PyMOL's Mutagenesis Wizard starting from the wild-type structure. Steric clashes (orange dashed lines) were identified using PyMOL's "find clashes" command, and polar contacts (yellow dashed lines) were identified using the corresponding PyMOL command. **(A)** The proposed V449D mutation is highlighted, which likely stabilizes the complex by removing a surface-exposed hydrophobic residue and introducing an intraprotomer polar contact. We speculate that substitution with glutamic acid, which HERMES-*amortized* predicts to have a comparable ranking to D449 in Fig. S14, would produce a similar stabilizing effect. **(B)** Introduction of an Arginine in place of a Valine at position 112 likely introduces an intraprotomer polar contact, as well as packing against the adjacent protomer (shown in green); we believe L112, which HERMES-*amortized* predicts to have a comparable ranking to R112 in Fig. S14, would also pack against the adjacent protomer better than Valine. **(C)** A159L stabilizes the complex likely due to a cavity-filling effect, albeit necessitating nearby I137 to adopt a different rotamer; we believe I159, which HERMES-*amortized* predicts to have a comparable ranking to L159 in Fig. S14, would also fulfill a similar role on the condition of L141 also adopting a different rotamer.

bioRxiv preprint doi: https://doi.org/10.1101/2024.07.09.602403; this version posted January 15, 2026. The copyright holder for this preprint (which was not certified by peer review) is the author/funder, who has granted bioRxiv a license to display the preprint in perpetuity. It is made available under a CC-BY 4.0 International license.

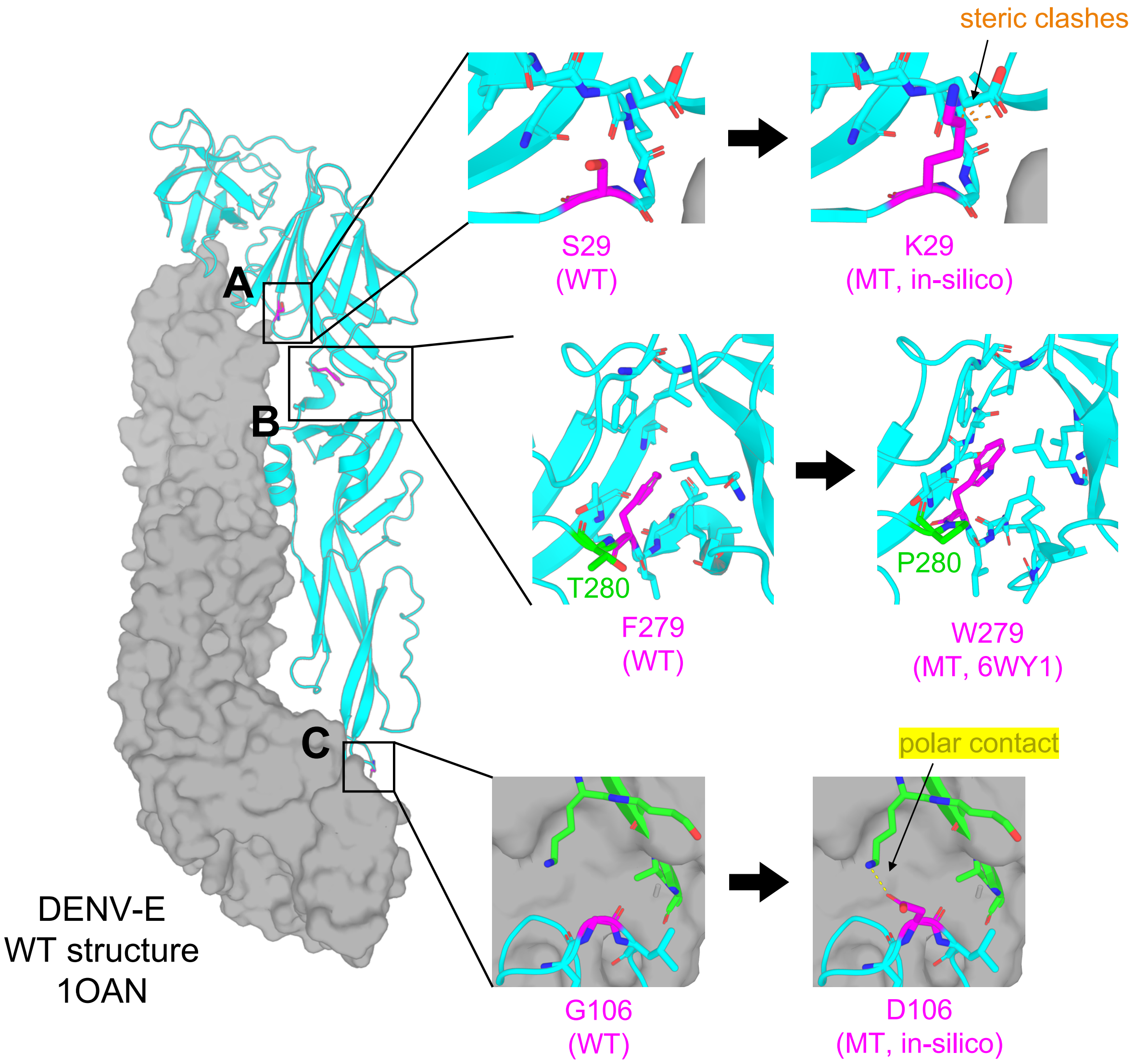

Figure S21 Structure of wild-type pre-fusion DENV-E antigen, with highlighted observed and candidate mutations. The wild-type structure is shown on the left (PDB ID 1OAN). A single protomer of the trimer is shown as cyan cartoon; the other copies are shown as grey surface. WT denotes wild type and MT denotes mutant. Mutations labeled as "in silico" were introduced using PyMOL's Mutagenesis Wizard starting from the wild-type structure. Steric clashes (orange dashed lines) were identified using PyMOL's "find clashes" command, and polar contacts (yellow dashed lines) were identified using the corresponding PyMOL command. **(A)** The S29K mutation is experimentally observed to be stabilizing [13], despite appearing to introduce substantial steric clashes when inspected in the wild-type structure, suggesting that stabilization requires a shift in backbone conformation. **(B)** The F279W mutation is experimentally observed to be stabilizing [13], likely by filling an under-packed cavity. A substantial backbone rearrangement is also observed in the mutant structure (residues 269–281), potentially facilitated by the T280P mutation. **(C)** The G106D mutation is experimentally observed to be stabilizing [13], likely through the introduction of a polar contact with the adjacent protomer (shown in green) and/or interactions with surrounding water molecules.
