# Supplementary material for: HERMES: Holographic Equivariant neuRal network model for Mutational Effect and Stability prediction
Source: bioRxiv. 2024 Oct 2:2024.07.09.602403. Originally published 2024 Jul 13. Preprint. [Version 2] doi: 10.1101/2024.07.09.602403 (PMC11257601; doi:10.1101/2024.07.09.602403)
Supplement: Supplementary file 1 [file NIHPP2024.07.09.602403V2-supplement-1.pdf]

bioRxiv preprint doi: https://doi.org/10.1101/2024.07.09.602403; this version posted October 2, 2024. The copyright holder for this preprint (which was not certified by peer review) is the author/funder, who has granted bioRxiv a license to display the preprint in perpetuity. It is made available under aCC-BY 4.0 International license.

A Appendix

A.1 Methods details

A.1.1 Pre-processing details

To generate our open source pre-processed training data, we use the following procedure: we use OpenMM [34] to fix the PDB files, add missing residues and substitute non-canonical residues for their canonical counterparts; we use the reduce program [35] to add hydrogens; we take partial charges from the AMBER99sb force field [36]; we use BioPython to compute SASA [28]. Both preprocessings procedures keep atoms belonging to non-protein residues and ions, unlike RaSP [2]. Notably, our PyRosetta preprocessings does *not* replace non-canonical residues.

A.1.2 Fine-tuning details

To greatly speed-up convergence, as a first step of fine-tuning we rescale the weight matrix and bias vector of the network's output layer so the mean and variance of the output logits become the same as that of the training scores. This step requires one initial pass through the training data to get the mean and variance, but it makes the model outputs immediately be in the same distribution as the scores, thus avoiding epochs of fine-tuning just devoted to rescaling the model outputs. We provide easy-to-use code to fine-tune our pre-trained models on arbitrary mutation effect data. Importantly, as we want to produce models using the convention that higher predicted mutation scores correspond to higher fitness, but we fine-tune on $\Delta\Delta G$ values which - since they are energy values - follow the reverse convention (lower $\Delta\Delta G$ means a more stable structure), our code fits the *negative* of Eq. 2 to the target values (wild-type score minus mutant score). In practice, to use the fine-tuning code, just make sure that lower means higher fitness, which can be done by simply flipping the sign of all the target values.

A.1.3 Use of ESMFold

We use the ESM Metagenomic Atlas API to fold each sequence individually (`https://esmatlas.com/resources?action=fold`).

A.1.4 Use of RaSP and Stability-Oracle datasets

RaSP. We use the RaSP data as provided on their github page (`https://github.com/KULL-Centre/_2022_ML-ddG-Blaabjerg`). The only difference we apply is in the Fermi transform. Since RaSP uses stability changes ($\Delta\Delta G$) computed with Rosetta, which are known to be accurate only in the [-7, 1] range, they pass them through a Fermi transform before training, which effectively "plateaus" outside the [-7, 1] range. We also use the Fermi transform, with the only difference that we *center* it so that 0 maps to zero. This is necessary since HERMES' utput space parameterization is such that the predicted stability change to the same amino-acid is zero ($\Delta\Delta G_{aa_i \rightarrow aa_i} = 0$, which is true of real $\Delta\Delta G$ also, but it is not true of the un-centered Fermi transform. Thus the equation we use is:

$$F(\Delta\Delta G) = \frac{1}{1 + e^{-\beta(\Delta\Delta G - \alpha)}} - \frac{1}{1 + e^{\beta\alpha}} \tag{S1}$$

Stability-Oracle. The main issue with the data provided by the authors in their github page (`https://github.com/danny305/StabilityOracle/tree/master`) is that the residue-numbers they provide do not align with the residue numbers in the original PDB files, but instead align with some post-processed representation of the structure which, at the time of writing this, is opaque and does not allow us to easily retrieve the original residue-numbers. Thus, we manually modified the datasets' csv files to have residue numbers match those found in the PDB files, and provide them in our repository.

bioRxiv preprint doi: https://doi.org/10.1101/2024.07.09.602403; this version posted October 2, 2024. The copyright holder for this preprint (which was not certified by peer review) is the author/funder, who has granted bioRxiv a license to display the preprint in perpetuity. It is made available under aCC-BY 4.0 International license.

A.1.5 SKEMPI

After filtering duplicate experiments, the dataset includes: 5,713 $\Delta\Delta G^{\text{binding}}$ values across 331 structures, of which 4,106 are single-point mutations across 308 structures. Further filtering for mutations that belong to structures with at least 10 mutations in the dataset, 116 structures remain with 5,025 total mutations; By restricting to only single-point mutations, we arrive at 93 structures and 3,485 mutations. We consider both “Per Structure" and “Overall" correlations. For multi-point mutations, we use an additive model and neglect epistasis.

The SKEMPI dataset conveniently provides information that helps in making train-test splits without data-leakage. Specifically, each mutation is provided with two pieces of information “hold-out type" and “hold-out proteins". Verbatim from their website (`https://life.bsc.es/pid/skempi2/info/faq_and_help`):

“5) The hold-out type. Some of the complexes are classified as protease-inhibitor (Pr/PI), antibody-antigen (AB/AG) or pMHC-TCR (TCR/pMHC). This classification was introduced to aid in the cross-validation of empirical models trained using the data in the SKEMPI database, so that proteins of a similar type can be simultaneously held out during a cross-validation.

6) The hold-out proteins. This column contains the PDB identifiers (in column 1) and/or hold-out types (column 5) for all the protein complexes which may be excluded from the training when cross-validating an empirical model trained on this data, so as to avoid contaminating the training set with information pertaining to the binding site being evaluated."

For the *Easy* split, we do not consider this information at all, and just split at random. For the *Medium* split, we simply make sure that, if a mutation is in a given split, then all of its “hold-out proteins" are in the same split as well, but not necessarily all of the proteins of the same “hold-out type"; these seem to mostly include closely-related proteins, or even the same exact protein bound to a different target. For the *Hard* split instead, we make sure that, if a mutation is in a given split, then all of the proteins of the same “hold-out type" are in the same split as well. This is overkill in practice, since for instance it precludes the use of any antibody-antigen data to predict on antibody-antigen complexes; it provides, however, a great test of generalization ability. We note that sometimes there are are proteins with multiple “hold-out types"; in these cases, we randomly chose one type for the protein.

A.2 Baselines

H-CNN [20]. We mention H-CNN because HERMES is effectively built on top of it, with HERMES 0.00and HERMES 0.50being directly comparable to it - except for the improved speed of HERMES' forward pass, which we tested by re-implementing the H-CNN architecture in our code. H-CNN is only trained on masked amino-acid prediction - our pre-training task. Its authors showed that H-CNN learned a model akin to a physical potential, and able to predict mutation effects of stability and binding via eq. 2, albeit only on two systems.

Stability-Oracle [22]. Similar to HERMES, Stability-Oracle is trained in two steps: first a graph attention model is pre-trained to predict masked amino-acids from their local atomic environment (i.e. “neighborhood"). The model regressing over mutation effects is then constructed and trained as follows. For a site on a structure, the masked neighborhood's embedding h is extracted from the pre-trained graph attention model. This embedding is concatenated with embeddings of the “from" and “to" amino-acids separately, and the two inputs are individually fed to a transformer network, yielding the two amino-acid specific embeddings $e_{aa_{\text{from}}}$ and $e_{aa_{\text{to}}}$. These are then substracted, and $(e_{aa_{\text{to}}} - e_{aa_{\text{from}}})$ is fed to a final 2-layer MLP that outputs a scalar representing $\Delta\Delta G_{aa_{\text{from}} \rightarrow \text{to}}$. Interesingly, up to right before the MLP, the output symmetries are not yet broken, because each e_{aa_i} is computed independently of any other amino-acid. The symmetries only get broken in the MLP: in fact, if the MLP were a linear layer with no bias, the symmetries would be respected. To make their model respect the symmetries, the authors train with data augmentation of reversibility and permutation.

RaSP [2]. Similar to HERMES, RaSP is trained in two steps: first, a neural network -

bioRxiv preprint doi: https://doi.org/10.1101/2024.07.09.602403; this version posted October 2, 2024. The copyright holder for this preprint (which was not certified by peer review) is the author/funder, who has granted bioRxiv a license to display the preprint in perpetuity. It is made available under aCC-BY 4.0 International license.

specifically a 3DCNN - is pre-trained to predict masked amino-acids from their local atomic environment (i.e. “neighborhood"). Then, a small fully-connected neural network with a single output is trained to regress over mutation effects, using as input neighborhoods' embeddings from the 3DCNN, the one-hot encodings of wildtype and mutant amino-acids, and the wildtype and mutant amino-acids' frequencies in the pre-training data. RaSP is fine-tuned on the stability effect of mutations $\Delta\Delta G$, computationally determined with Rosetta [27], which we also use to fine-tune HERMES. We do not reproduce results of RaSP in this work, and instead show the values reported in the paper.

ProteinMPNN [37]. ProteinMPNN is a tool for protein inverse-folding. The tool is most commonly used to sample amino-acid sequences conditioned on a protein's backbone structure, and optionally a partial sequence. As ProteinMPNN also outputs probability distributions of amino-acids for the sites that are to be designed, it can also be used to infer mutation effects by computing the log-likelihood ratio presented in eq. 1. Like for HERMES, we consider ProteinMPNN models trained with two noise levels: 0.02 Å(virtually no noise) and 0.30 Å. We provide scripts to infer mutation effects built upon a public fork of the ProteinMPNN repository.

ESM-1v [19]. This is the Protein Language Model (PLM) of the ESM family trained specifically for improved zero-shot predictions of mutation effects. As the training objective is predicting amino-acids that have been masked from the sequence, mutation effects are also predicted using the log-likelihood ratio (eq. 1). To our knowledge, this is the strongest representative of PLMs for inferring mutation effects. We show a mix of previously-reported scores, and scores computed using their codebase. For our in-house ESM-1V predictions, wildtype sequences were obtained from the corresponding PDB file and verified against the European Bioinformatics Institute's PDBe database via their REST API [38]. Mutation effect predictions were computed with ESM's built-in *wildtype marginal* method; we attempted using the *masked marginal* method but ran into several errors, so we stuck to *wildtype marginal* as it was more reliable, and also had very similar performances in the few instances in which both methods worked.

DeepSequence [18]. This is a state-of-the-art model for inferring mutation effects from sequence alone. It uses a variational auto-encoder of full protein sequences to and infers mutation effects via eq. 1. We only show previously-reported scores.

A.3 Extended Results

A.3.1 Wildtype amino-acid classification

In Table S1 we show Classification Accuracy of HERMES models, when predicting the amino-acid identity of the masked residue at the center of a neighborhood. Adding noise during training, as well as fine-tuning over stability effects, reduces the model's predictions of the wild-type. Models that were *not* pre-trained on amino-acid classification, and only trained on stability effects, predict the wildtype only barely more than random. As seen in Figure 3, the models' bias in predicting the wildtype most commonly found in nature.

A.3.2 Results on predicting Deep Mutational Scanning assays

We evaluate model performance on 27 out of the 41 Deep Mutational Scanning (DMS) studies collected by [18] and considered by [19]. To simplify the analysis, we consider only the 37 studies containing single-point mutations only. For these, only the proteins' sequences were available to us a priori. Starting from the sequences, we augmented the dataset with both experimental structures that we identified in the RCSB website [1] and AlphaFold2 structures, either from the AlphaFold database [2], or folded using the AlphaFold2 [39] google colab with default parameters. Keeping only studies with at least one high-quality structure, we were left with 25 studies, many of which with only the AlphaFold-generated structure. Some proteins have multiple experimental structures, as in each structure they are bound to different a different and it was not obvious from the study of origin which ligand was more appropriate. We provide structures and detailed notes for each study on our github repository.

[1] `https://www.rcsb.org/`

[2] `https://alphafold.ebi.ac.uk/`

bioRxiv preprint doi: https://doi.org/10.1101/2024.07.09.602403; this version posted October 2, 2024. The copyright holder for this preprint (which was not certified by peer review) is the author/funder, who has granted bioRxiv a license to display the preprint in perpetuity. It is made available under aCC-BY 4.0 International license.

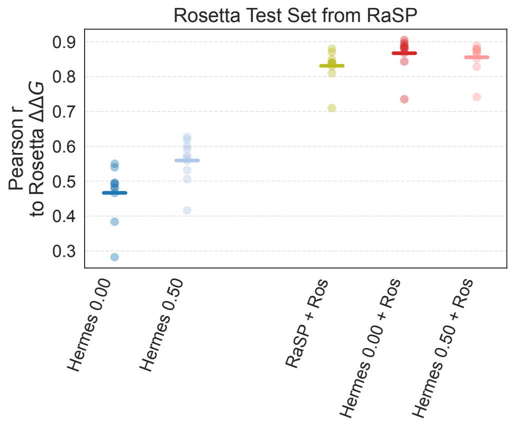

Figure S1: **Pearson correlation of predictions against RaSP's test set of Rosetta-computed stability effects for 10 proteins [2]** Each dot is a protein; the horizontal bar is the mean. HERMES models achieve better Pearson correlation using the same training data. We observe that centering the Fermi transform (Eq. S1) provided a slight boost in performance.

In Figures S7 and S8 we show absolute Pearson and Spearman correlations between model predictions and expreiments for the 27 studies, selected as described above. We use absolute values for simplicity, as assays may have either positive or negative sign associated with higher fitness. Patterns are similar to those we found for the stability effect of mutations $\Delta\Delta G$: training with noise improves pre-trained-only models, and so does pre-processing with PyRosetta. Models fine-tuned on stability effects see their performance improved. However, the best structure-based model (HERMES 0.00 + Ros 0.50 + FT with mean Pearson r of 0.40) still performs significantly worse, on average, compared to the state-of-the-art sequence-based models (DeepSequence [18] with 0.50, and ESM-1v [19] with 0.47).

Table S1: **Performance of HERMES models on wildtype amino-acid classification on 40 CASP12 test proteins.** As expected, models trained with noise have worse Accuracy. Interestingly, models fine-tuned on stability $\Delta\Delta G$ values retain part of their accuracy, whereas models that are *only trained* for stability prediction have almost no predictive power of the wildtype amino-acid. Differences between using the Pyrosetta and Biopython pipelines are negligible.

**Model**	**Pyrosetta Accuracy**	**Biopython Accuracy**
HERMES 0.00	0.73	0.75
HERMES 0.50	0.64	0.65
HERMES 0.00 + Ros	0.41	0.40
HERMES 0.50 + Ros	0.38	0.37
HERMES 0.00 + cDNA117k	0.47	0.45
HERMES 0.50 + cDNA117k	0.39	0.38
HERMES 0.00 + cDNA117k train ESMFold	0.46	0.49
HERMES 0.50 + cDNA117k train ESMFold	0.40	0.40
HERMES Untr. 0.00 + cDNA117k	0.09	-
HERMES Untr. 0.50 + cDNA117k	0.08	-

bioRxiv preprint doi: https://doi.org/10.1101/2024.07.09.602403; this version posted October 2, 2024. The copyright holder for this preprint (which was not certified by peer review) is the author/funder, who has granted bioRxiv a license to display the preprint in perpetuity. It is made available under aCC-BY 4.0 International license.

Table S2: **Results on predicting single-point mutation effects on protein-protein binding in SKEMPI.** Results above the double-line are taken from [21]; see their paper for a detail introduction of each model being compared ([40, 41, 42, 43, 44, 45]). The HERMES models most comparable - in terms of training procedure - to the models reported by [21] are the models trained on the *Easy* split: for it, we use 3-fold cross-validation on datasets split by PDB structure without further restrictions. However, we do not know which exact PDBs are in the splits for [21] and could not recover them from their codebase.

**Method**	**Per-Struct. Pearson**	**Per-Struct. Spearman**	**Overall Pearson**	**Overall Spearman**
ESM-1v	0.0422	0.0273	0.1914	0.1572
PSSM	0.1215	0.1229	0.1224	0.0997
MSA Transf.	0.1415	0.1293	0.1755	0.1749
Tranception	0.1912	0.1816	0.1871	0.1987
Rosetta	0.3284	0.2988	0.3113	0.3468
FoldX	0.3908	0.3640	0.3560	0.3511
DDGPred	0.3711	0.3427	0.6515	0.4390
End-to-End	0.3818	0.3426	0.6605	0.4594
B-factor	0.1884	0.1661	0.1748	0.2054
ESM-IF	0.2308	0.2090	0.2957	0.2866
MIF-Δlogit	0.1616	0.1231	0.2548	0.1927
MIF-Net.	0.3952	0.3479	**0.6667**	0.4802
RDE-Linear	0.3192	0.2837	0.3796	0.3394
RDE-Net.	0.4687	**0.4333**	0.6421	**0.5271**
ProteinMPNN 0.02	0.2813	0.2824	0.3307	0.3153
ProteinMPNN 0.30	0.2702	0.2549	0.3344	0.2893
HERMES 0.00	0.3064	0.2866	0.2854	0.2721
HERMES 0.50	0.3168	0.3075	0.2910	0.2863
HERMES 0.00 + Ros	0.3453	0.3072	0.4011	0.3522
HERMES 0.50 + Ros	0.3357	0.3069	0.3713	0.3276
HERMES 0.00 + cDNA117k	0.3467	0.3307	0.3802	0.3419
HERMES 0.50 + cDNA117k	0.3046	0.2943	0.3443	0.2881
HERMES 0.00 + cDNA117k train ESMFold	0.3405	0.3350	0.3957	0.3375
HERMES 0.50 + cDNA117k train ESMFold	0.3093	0.2939	0.3643	0.3079
HERMES 0.00 + Skempi Easy	0.4707	0.4331	0.5781	0.4761
HERMES 0.50 + Skempi Easy	0.4296	0.3892	0.5120	0.4203
HERMES 0.00 + Skempi Medium	**0.4716**	0.4302	0.5762	0.4655
HERMES 0.50 + Skempi Medium	0.4074	0.3676	0.4966	0.4029
HERMES 0.00 + Skempi Hard	0.4353	0.3979	0.3954	0.3802
HERMES 0.50 + Skempi Hard	0.3988	0.3592	0.3280	0.3216

bioRxiv preprint doi: https://doi.org/10.1101/2024.07.09.602403; this version posted October 2, 2024. The copyright holder for this preprint (which was not certified by peer review) is the author/funder, who has granted bioRxiv a license to display the preprint in perpetuity. It is made available under aCC-BY 4.0 International license.

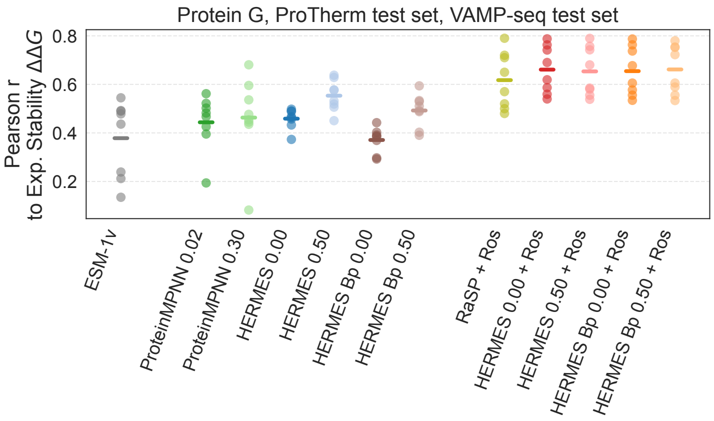

Figure S2: **Pearson correlation of predictions against RaSP's test set of experimental stability effects for 8 proteins [2].** Each dot is a protein; the horizontal bar is the mean. Zero-shot HERMES models perform similarly to ProteinMPNN models, with noise consistently improving performance. Zero-shot HERMES models using the Biopython pipeline are slightly worse. Differences between noise level and pre-processing pipeline become insignificant after fine-tuning. Notably, HERMES models achieve better Pearson correlation than RaSP using the same training data.

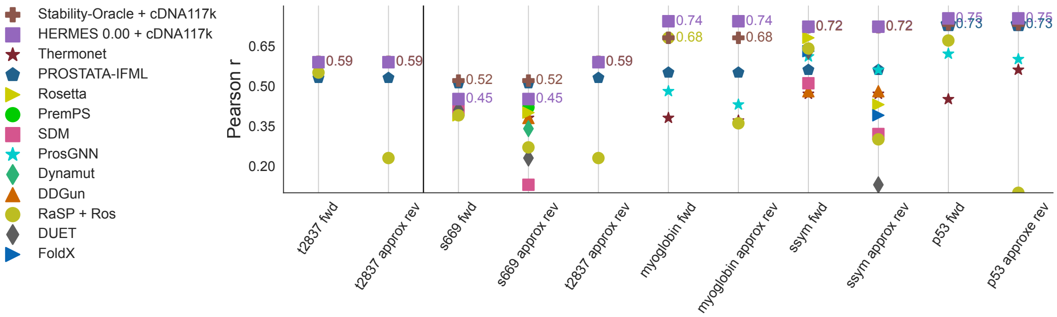

Figure S3: **Pearsonr correlation of several models' predictions and experimental stability effects, from the T2837 dataset and its subsets.** This is effectively a replica of a figure in [22]. Results of all models other than HERMES were taken from [22]. We label the correlations on "reverse" mutations as *approximate* because predictions were made with conditioning only on the wildtype structures. As discussed in the Methods section, HERMES respects approximate permutational anti-symmetry (i.e. "forward" and "reverse" mutations are anti-symmetric) by design, without the need for data augmentation.

bioRxiv preprint doi: https://doi.org/10.1101/2024.07.09.602403; this version posted October 2, 2024. The copyright holder for this preprint (which was not certified by peer review) is the author/funder, who has granted bioRxiv a license to display the preprint in perpetuity. It is made available under aCC-BY 4.0 International license.

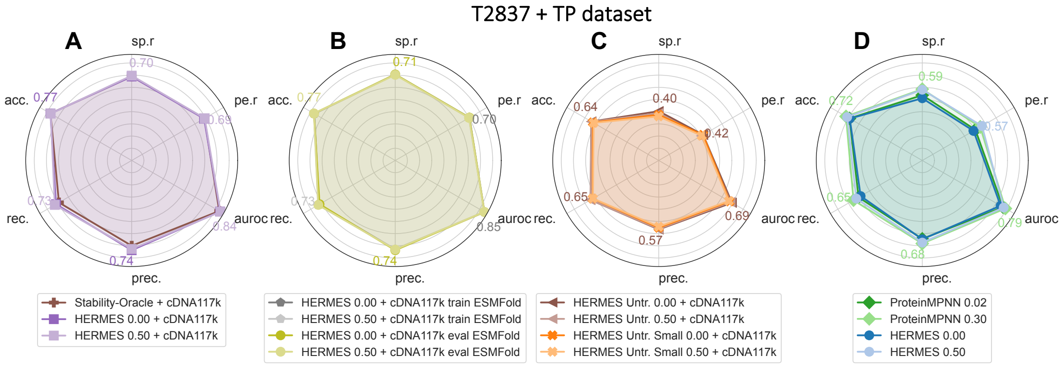

Figure S4: **Predicting stability effect of mutations in T2837 + TP dataset.** The Pearson correlation (pe.r), Spearmann correlation (sp.r), accuracy (acc.), recall (rec.), precision (prec.), and AUROC are shown for different models. “TP" is short for “Thermodynamic Permutations", i.e. the data augmentation technique of permutational anti-symmetry devised by [22]. Similar trends as in Figure 2 are observed.

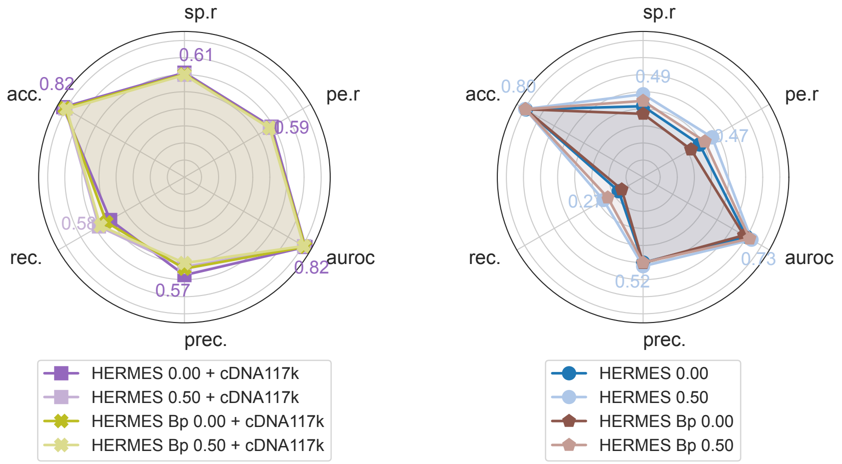

Figure S5: **Predicting stability effect of mutations in T2837: comparison between Pyrosetta and Biopython pipelines.** Similar to the results on RaSP data (Figure S2) models using Biopython pre-processing perform a bit worse than those using Pyrosetta, but the difference is rendered insignificant after fine-tuning.

bioRxiv preprint doi: https://doi.org/10.1101/2024.07.09.602403; this version posted October 2, 2024. The copyright holder for this preprint (which was not certified by peer review) is the author/funder, who has granted bioRxiv a license to display the preprint in perpetuity. It is made available under aCC-BY 4.0 International license.

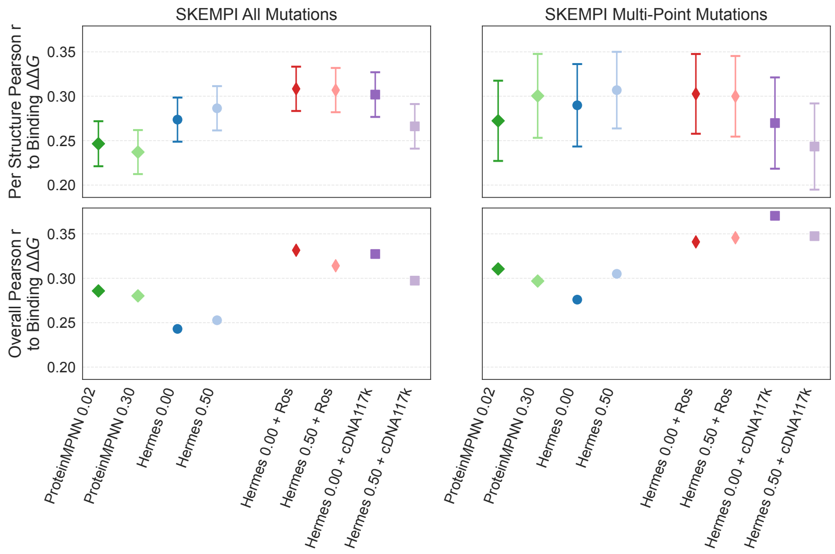

Figure S6: **Pearson correlation on SKEMPI multi-point mutations.**.

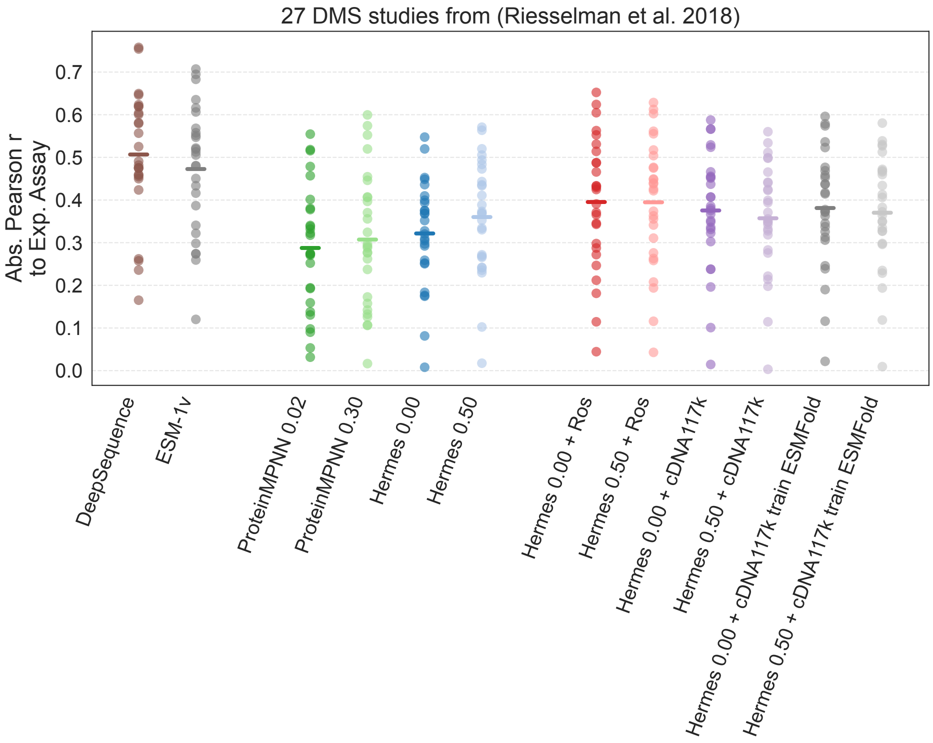

Figure S7: **Pearson correlation of models' predictions against DMS experimental assays from [18].** Each point is a study (single protein), and horizontal bars are mean values. Fine-tuning HERMES models on stability $\Delta\Delta G$ values improves performance, but it does not enable them to reach the levels of state-of-the-art sequence-based models DeepSequence and ESM-1v.

bioRxiv preprint doi: https://doi.org/10.1101/2024.07.09.602403; this version posted October 2, 2024. The copyright holder for this preprint (which was not certified by peer review) is the author/funder, who has granted bioRxiv a license to display the preprint in perpetuity. It is made available under aCC-BY 4.0 International license.

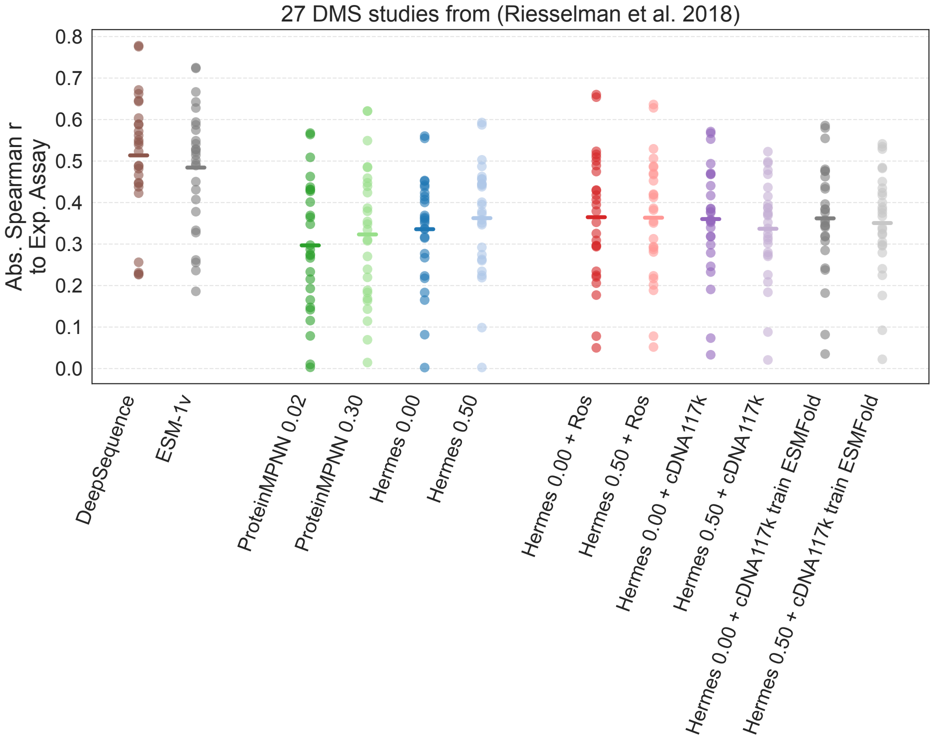

Figure S8: **Spearman correlation of models' predictions against DMS experimental assays from [18].** Each point is a study (single protein), and horizontal bars are mean values. Fine-tuning HERMES models on stability $\Delta\Delta G$ values improves performance, but it does not enable them to reach the levels of state-of-the-art sequence-based models DeepSequence and ESM-1v.
